# A Novel, Scalable Social Media–Based Intervention (“Warna-Warni Waktu”) to Reduce Body Dissatisfaction Among Young Indonesian Women: Protocol for a Parallel Randomized Controlled Trial

**DOI:** 10.2196/33596

**Published:** 2022-01-28

**Authors:** Kirsty May Garbett, Nadia Craddock, Sharon Haywood, Kholisah Nasution, Paul White, L Ayu Saraswati, Bernie Endyarni Medise, Girl Effect, Percolate Galactic, Phillippa C Diedrichs, Heidi Williamson

**Affiliations:** 1 Centre for Appearance Research University of the West of England Bristol United Kingdom; 2 Faculty of Medicine Universitas Indonesia Jakarta Indonesia; 3 Applied Statistics Group University of the West of England Bristol United Kingdom; 4 Department of Women, Gender, and Sexuality Studies University of Hawaii Manoa, HI United States

**Keywords:** body image, body dissatisfaction, Indonesia, adolescent, mental health, randomized controlled trial, study protocol, eHealth intervention, Southeast Asia, young adult, teenager, women, social media, intervention, image, protocol, mood, satisfaction

## Abstract

**Background:**

Despite the prevalence of body dissatisfaction among young Indonesian women and its consequential negative impacts, there are currently no evidence-based, culturally appropriate interventions to tackle this issue. Therefore, there is a need to develop scalable, cost-effective, and accessible interventions to improve body image among this population.

**Objective:**

This paper describes the study protocol of a parallel randomized controlled trial to evaluate the effectiveness of *Warna-Warni Waktu*, a social media–based intervention that aims to reduce state and trait body dissatisfaction and improve mood among young Indonesian women aged 15-19 years.

**Methods:**

The trial will take place online. Approximately 1800 young women from 10 cities in Indonesia, evenly split across the ages of 15-19 years, will be recruited via a local research agency’s established research panel. Participants will be randomly allocated to the intervention condition or a waitlist control condition. The intervention consists of six 5-minute videos, with each video supplemented with up to five brief interactive activities. The videos (and associated activities) will be delivered at a rate of one per day across 6 days. All participants will complete three self-report assessments: at baseline (Day 1), 1 day following the intervention (Day 9), and 1 month following the intervention (Day 36). The primary outcome will be change in trait body dissatisfaction. Secondary outcomes include change in internalization of appearance ideals, trait mood, and skin shade satisfaction. Intervention effectiveness on these outcomes will be analyzed using linear mixed models by a statistician blinded to the randomized condition. Intervention participants will also complete state measures of body satisfaction and mood before and after watching each video to assess the immediate impact of each video. This secondary analysis of state measures will be conducted at the within-group level.

**Results:**

Recruitment began in October 2021, with baseline assessments underway shortly thereafter. The results of the study will be submitted for publication in 2022.

**Conclusions:**

This is the first study to evaluate an eHealth intervention aimed at reducing body dissatisfaction among young Indonesian women. If effective, the intervention will be disseminated to over half a million young women in Indonesia via Facebook, Instagram, and YouTube.

**Trial Registration:**

ClinicalTrials.gov NCT05023213; https://clinicaltrials.gov/ct2/show/NCT05023213

**International Registered Report Identifier (IRRID):**

PRR1-10.2196/33596

## Introduction

Body dissatisfaction, defined as the subjective experience of negative thoughts toward one’s own body [1], is a growing concern among young people globally [2]. Young women are disproportionately affected compared to young men [3-5]. Although research in Asia is currently limited, cross-cultural research suggests that the prevalence of body dissatisfaction among young people in some Asian countries such as Malaysia, China, and Japan is similar to, if not greater than, that of English-speaking countries [6-8].

Body dissatisfaction is not benign. Extensive research has established that body dissatisfaction is associated with numerous adverse health outcomes. Longitudinal research has found body dissatisfaction to predict eating disorders [9,10], depressive mood and low self-esteem [11], less engagement in exercise [10], and increased risky health behaviors such as drug use and smoking [12]. Research also indicates associations between body dissatisfaction and the desire for cosmetic surgery [13,14], and the avoidance of everyday activities such as participating in school activities or attending classes [15,16]. Although the bulk of this research has been concentrated within English-speaking populations, similar associations are emerging globally, including across Asia [6,17-20].

Indonesia is an upper-middle-income country in Southeast Asia and is the world’s fourth most populous country [21]. In the Indonesian context, research shows that more than half of young women experience at least some dissatisfaction with their appearance (unpublished data compiled by the authors KG, NC, LS, and PD, 2021) [22]. Body dissatisfaction among young Indonesian women is linked with disordered eating behaviors [23], including food restriction and avoidance, emotional eating, and excessive exercise (unpublished data by authors KG, NC, LS, and PD, 2021). Further, young Indonesian women report body dissatisfaction specifically with regard to their skin shade [24,25], due in part to the dominant pan-Asian ideal prevalent across Asia, which emphasizes light skin shades [26]. In other populations (eg, India), skin shade dissatisfaction has been associated with the use of potentially harmful skin fairness products [27], lower self-esteem [28], and, unsurprisingly, general body image concerns [28]. As such, ameliorating body dissatisfaction, including skin shade dissatisfaction, among young women in Indonesia is required. Yet, no published evaluations of interventions targeting body dissatisfaction in Indonesia exist.

The creation and dissemination of mental health prevention efforts and interventions across low- and middle-income countries (LMICs), including Indonesia, face cultural-specific obstacles, primarily social stigma connected to mental health concerns, coupled with a lack of mental health professionals and the limited capacity of general health professionals in providing effective mental health treatment [29,30]. Although Indonesia has seen a modest increase in mental health interventions for adolescents in school settings in recent years [31-33], evidence suggests that implementation is not commonplace [34], thus highlighting the need for the dissemination of educational mental health content outside the school system. Digital interventions present a solution to circumvent barriers to dissemination, and have been shown to have similar effectiveness as face-to-face psychotherapeutic interventions [35]. Digital interventions are relatively low in cost, easily accessible, and universally available, thereby meeting three key criteria for overcoming LMIC-specific challenges [36,37]. Further, private, remotely accessible mental health interventions have been shown to increase the likelihood of engagement in help-seeking due to the reduced fear of stigma [38].

eHealth interventions have the potential to reach many young people in Indonesia; according to the Indonesian Internet Providers Association, over 90% of adolescents aged 15-19 years have access to the internet across the country [39]. Moreover, research indicates that young people are already using the internet to seek information [40,41]. Thus, it is perhaps unsurprising that eHealth interventions for mental health have shown preliminary acceptability among young people in Indonesia. For example, eHealth interventions for depression prevention and/or treatment have been well received, with young people showing a willingness to engage with such interventions [42,43].

Social media offer unprecedented capabilities to disseminate mental health interventions cost-effectively and at scale [44], and may be particularly popular with young people. Social media interventions afford unique opportunities to overcome barriers such as cost, geographic distance, and stigma, as they allow for a certain degree of privacy and anonymity. Emerging research suggests that using social media in Indonesia as a vehicle for eHealth interventions shows promise among young people [45,46]. Thus, the combination of the rise in eHealth initiatives for adolescents in LMICs [47] and the notable uptake in social media usage among young people in Indonesia in recent years [48,49] have created a ripe environment for the development of a social media–based intervention to address body dissatisfaction among young Indonesian women. Furthermore, research consistently shows the social media environment in general to be problematic for young people’s body image [50,51], due in large part to the objectification and idealization of women’s bodies. As such, hosting an intervention to reduce body dissatisfaction on social media may be additionally beneficial in disrupting the harmful effects these sites have been shown to have. Increasingly, the potential for positive content on social media is being explored, which has highlighted the benefits of some types of content on mood and body satisfaction [52,53].

This paper outlines the development and protocol for the evaluation of the first social media–based intervention to target body dissatisfaction among young women in Indonesia, named *Warna-Warni Waktu* (English translation: Colorful Time Travel). The intervention was developed by the academic authors of this paper in collaboration with Girl Effect, an international nonprofit organization that builds media content aiming to arm girls with the skills to make positive choices and changes in their lives during the critical years of adolescence; the Dove Self-Esteem Project, the social mission for Unilever’s personal care brand, Dove; Percolate Galactic, an Indonesian-based creative agency that specializes in marketing for youth; and young Indonesian women. The intervention consists of a series of six videos, each approximately 5 minutes long. To disseminate the intervention, the videos will be sequentially delivered to young women in Indonesia through targeted social media marketing on Facebook and Instagram, in addition to being made freely available on YouTube. On Facebook and Instagram, the videos will appear on young Indonesian women’s feeds, consistent with other social media advertisements. The series tells the fictitious story of a young woman named Putri who learns strategies to resist appearance pressures across adolescence and young adulthood through the help of animated time travelers, who are on a quest to save the world from appearance-related pressures. The intervention is based upon mounting evidence that psychoeducation, in particular discussing the nature, causes, and consequences of body dissatisfaction, is an effective change technique to reduce body dissatisfaction [54]. Further, the intervention’s videos model behaviors to reduce appearance pressure and teach media literacy skills, two further change techniques that have shown efficacy in reducing body dissatisfaction in previous studies [55-57].

The videos target three sociocultural influences of body dissatisfaction, based on the Tripartite Influence Model of body dissatisfaction [58], namely, the media, friends, and family. The Tripartite Influence Model postulates that body dissatisfaction increases via the impact that these sociocultural influences have on two psychological processes: internalization of appearance ideals and social comparisons. These two psychological processes are also addressed directly in the videos by providing media literacy education and examining the consequences of making appearance-based social comparisons. As such, we anticipate that the intervention will reduce body dissatisfaction through diminishing an individual’s perceived appearance pressure from the media (including social media), friends, and family members, which in turn will reduce their internalization of appearance ideals and the likelihood of making social comparisons.

The potential impact of the videos is further bolstered through supplementary activities, which again will be disseminated to young Indonesian women via sequential social media marketing. Each video is accompanied by interactive activities, which aim to reinforce the lessons learned in each video. Research consistently shows that elements of active learning (ie, taking control of one’s own learning through metacognitive sense-making, self-assessment, and reflection [59]) results in deeper learning [60] and higher engagement levels [61]. Further, eliciting cognitive dissonance has been shown to be a key change technique in reducing body dissatisfaction [54]. As such, active learning activities based upon cognitive dissonance were built into the intervention to be delivered between each video. These include activities such as story completion, self-reflection, writing challenges, and word searches. Details of each activity, along with a breakdown of each video’s content, are outlined in Textbox 1.

**Intervention summary.**

**Video One: Time to Turn Back Time**

*Key messages:*

- People face pressure to look a certain way.
- Pressure comes from the media, as well as from those around us.
- Appearance pressures are associated with body dissatisfaction and can hold us back from living a fulfilling life.
- Strategies can be learned to resist and challenge appearance pressures.

*Reinforcer activities:*

- Review definitions of key terms in five short videos. Using some of the key terms, explain why the time travelers want Indonesian girls to feel confident about their bodies.

**Video Two: That’s Fake! (targeting social media)**

*Key Messages:*

- The media often portray just one narrow appearance ideal.
- Images of people in the media are often edited to make the person look more like the appearance ideal.
- The media set an unrealistic appearance standard in order to sell us products.
- We can curate our own media environment to reduce the appearance pressures that we face.

*Reinforcer activities:*

- Describe your experience of coming across advertisements that promote unrealistic beauty products to help others become more aware.
- Share the transformation video clip seen in this episode on social media, and explain why it is important your friends and followers watch it.
- An edited and unedited image is provided. Identify and list all the edits made to the image.
- Watch the video Putri saw advertising skin-lightening cream, and critically examine its messages about the lifestyle the advert is trying to sell. Share your experience buying (or thinking about buying) a product that you thought would improve your popularity or lifestyle.
- Your Own Words activity: Write about why skin-lightening products are problematic.

**Video Three: C’mon, Break the Chain of Comparisons (targeting appearance-based comparisons)**

*Key messages:*

- Appearance-based comparisons are common.
- Engaging in appearance-based comparisons is unhelpful and damaging to body image.
- Focusing on what your body can do, rather than how it looks, is a more helpful way to think about your body.

*Reinforcer activities:*

- Write a sentence or two on why you appreciate your friends, describing things that have nothing to do with appearance.
- Complete the comic strip: In the context of appearance-based comparisons among friends, explain why appearance-based comparisons are not helpful or necessary.
- Your Own Words activity: Write about all the things you love about your friends that have nothing to do with appearance.

**Video Four: Stand up to Appearance-Based Comments (targeting appearance-based teasing)**

*Key messages:*

- Comments from friends and family about our appearance can be hurtful, even if well-meaning.
- Challenging such comments in a nonconfrontational way can prevent future comments from family members, alleviating appearance pressures.

*Reinforcer activities:*

- Write a short response to a negative appearance-based comment received from a friend or family member.
- Word search: Find 10 hidden example phrases of how to respond to appearance-based comments.
- Complete the comic strip: How to respond to boys teasing girls about their appearance.
- Your Own Words activity: Write about how to stand up to appearance-based comments.

**Video Five: Be Your Own Best Friend! (targeting body talk)**

*Key messages:*

- Negative body talk is harmful to our body image.
- Creating a mantra to repeat to oneself instead of getting caught up in negative self-talk is an effective strategy to break the cycle of negative body talk.

*Reinforcer activities:*

- Create a mantra that can boost your confidence.
- List things that your body allows you to do.
- Your Own Words activity: Write about your mantra, sharing why you created it and what it means to you.

**Video Six: The Color of the Future**

*Key messages:*

- By challenging appearance pressures in everyday life, we can reach our full potential.
- The additive impact of resisting and challenging appearance pressures is large, not only for the individual but also for wider society.

*Reinforcer activities:*

- Watch a short video of Putri sharing the four key lessons she learned throughout her journey. Identify which lesson is most important to you and why.
- Commit to sharing an unedited photo on social media doing something you love.

In addition to detailing the intervention and its development, this paper describes the evaluation protocol for assessing the effectiveness of the intervention among young Indonesian women aged 15 to 19 years through a parallel, two-arm (intervention vs waitlist control) web-based randomized controlled trial (RCT). The decision to utilize a waitlist control condition was informed by recommendations from a National Institutes of Health expert panel [62]. Specifically, it is recommended that the rationale for a comparator group should rest on the primary purpose of the trial. Thus, given that the primary interest of this study was the absolute impact of the intervention rather than the relative impact, and that usual care for body dissatisfaction in Indonesia is no care at all, a waitlist control condition was deemed most appropriate for this purpose. Our hypotheses for this research are: (1) participants randomized to the intervention condition will experience reduced body dissatisfaction, internalization of appearance ideals, and skin shade dissatisfaction, as well as improvements in mood, at postintervention and 1-month follow-up, relative to the waitlist control condition; (2) each video in the *Warna-Warni Waktu* series will elicit immediate state-based improvements in body satisfaction and mood; and (3) greater engagement and adherence in the *Warna-Warni Waktu* intervention will result in greater reductions in body dissatisfaction, internalization of appearance ideals, and skin shade dissatisfaction, as well as greater improvements in mood. This analysis will be exploratory in nature, depending on the participants’ engagement and adherence in the intervention during the trial.

## Methods

### Study Design

The study is a two-arm parallel RCT with an intervention group and a waitlist control group. Randomization will be performed as block randomization with a 1:1 allocation. Participants in the intervention condition will receive the *Warna-Warni Waktu* intervention over a period of 6 days. Participants in the waitlist control condition will receive a link to the *Warna-Warni Waktu* series at the end of the trial. The protocol was designed in accordance with the SPIRIT (Standard Protocol Items: Recommendations for Interventional Trials) guidelines (Multimedia Appendix 1).

For the trial, the videos will not be distributed to recruited participants via social media. Rather, the intervention will be recreated for distribution via Qualtrics software. Six Qualtrics links will be developed, one for each video and its corresponding supplementary activities, and sent to participants daily over a 6-day period. The trial has been designed in this way so that there is an accurate log of intervention adherence at the individual level, an important consideration when evaluating web-based interventions [63]. Careful consideration was given to ensure that the intervention was accurately produced on Qualtrics to appear as closely as possible to how it will look when it is disseminated on social media in the future (see Multimedia Appendix 2).

### The Intervention

**Figure 1:**
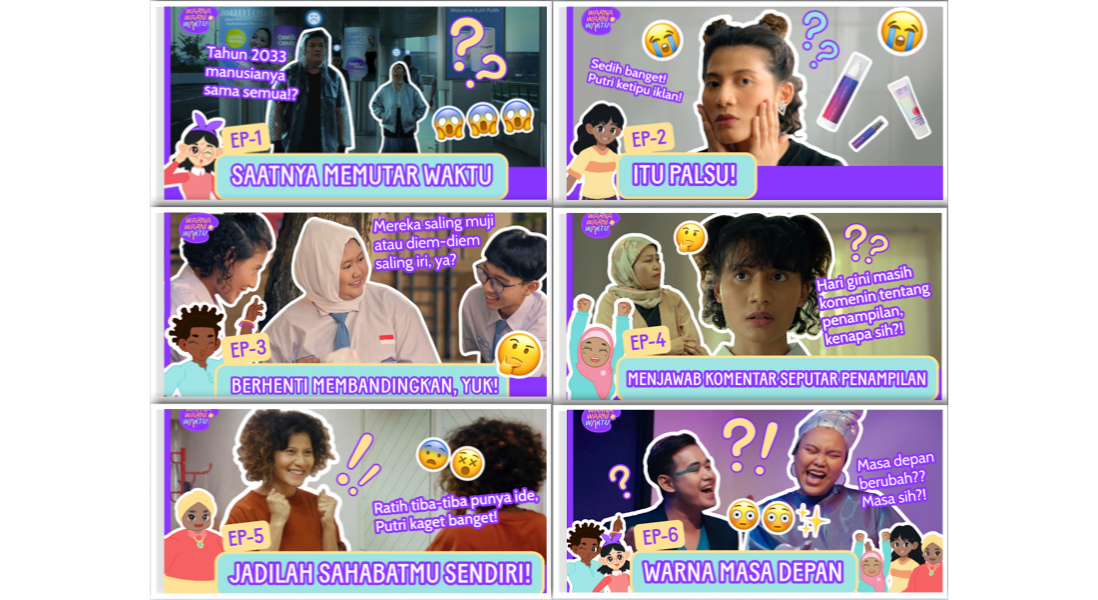
The video thumbnails for each of the 6 episodes in *Warna-Warni Waktu*.

**Figure 2:**
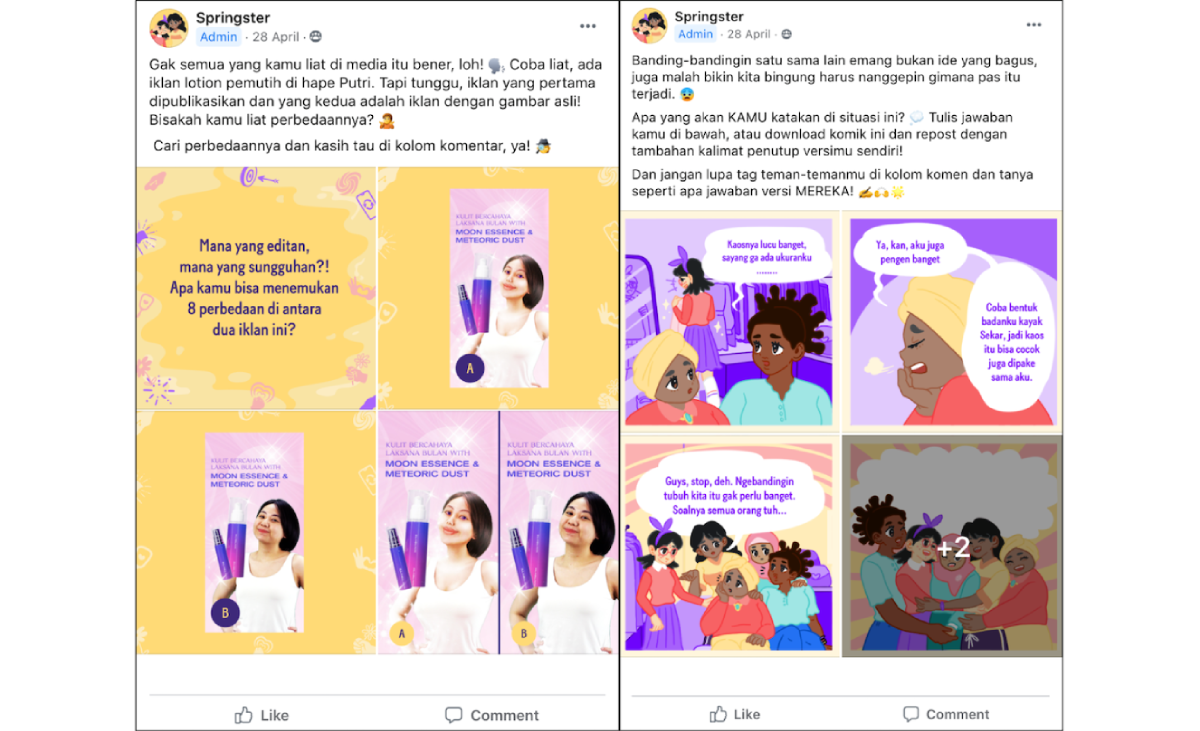
Example activities. The activity on the left asks users to identify the differences between an edited and unedited photo (Video 2, Activity 3). The activity on the right asks users to complete the comic strip with a comment about why appearance-based comparisons are unnecessary (Video 3, Activity 2).

The intervention *Warna-Warni Waktu* was developed over a 20-month period, from October 2019 to May 2021. Textbox 2 presents the intervention development steps taken, which involved close collaboration among body image academics, a creative agency, social media specialists, a nonprofit organization, as well as an industry funder. The intervention development team consisted of numerous women with lived experience of Indonesian culture; namely, five of the six core team members from the creative agency (Percolate Galactic) were young Indonesian women between the ages of 25 and 30. The wider team also consisted of two female Indonesian pediatricians (authors KN and BM), who have lived experience of the culture as well as daily contact with young Indonesian women through their work. Finally, the team included an Indonesian Professor of Women’s Studies (LS). Further, the process involved three rounds of feedback from young Indonesian women at various stages of development to create an intervention that is both for, and created with, young Indonesian women. The final intervention comprises 6 videos between 4 and 5 minutes each (see Figure 1) and 18 interactive reinforcer activities, ranging from approximately 2 to 10 minutes each in length (see Figure 2 for examples). All content is delivered to young women in Bahasa Indonesia, the official language of Indonesia. The activities encourage the target audience to reflect and apply the learnings from the videos to their own lives (Textbox 1).

**Intervention development stages.**

**Literature review: October 2019-January 2020**

- Common appearance concerns among young Indonesian women include feeling pressure about their weight [64,65], skin shade [25], and skin complexion [66].
- Prominent sources of appearance pressure contributing to negative body image among young Indonesian women were identified as cyberbullying [67,68], appearance comparisons [66], and social media [66].
- eHealth interventions in low- and middle-income countries are increasing in number [47] and are acceptable among young people in Asia [69-72].

**Secondary data analysis: December 2019-April 2020**

- Secondary data analysis was conducted on data collected from 318 Indonesian girls and young women who participated in The Dove Global Girls Beauty and Confidence Report [73], corroborating the literature review results and further identifying internalization of appearance ideals and self-esteem as important influences of body image concerns (unpublished data of the authors KG, NC, LS, and PD, 2021).

**U-Report poll on appearance-related concerns: February 2020**

- In collaboration with UNICEF Indonesia, a U-Report poll was conducted among 1441 young women from all 34 Indonesian provinces for an up-to-date assessment of the role body image plays in the lives of young people.
- More than three-quarters of young women wanted to change something about their appearance, and nearly half reported that worrying about their appearance prevented them from doing things they would like to do. Nearly all young women reported that they would like to learn ways to improve how they feel about their appearance [74].

**Focus groups: March 2020**

- Each of the six focus groups (one face-to-face and five online due to the COVID-19 pandemic) consisted of five or six girls aged 13-18 years from Jakarta province.
- Appearance-based teasing and comments as well as pressure from social media (particularly influencers) were identified as prominent sources of appearance pressure.
- Positive body image traits (such as valuing body functionality and defining beauty broadly, beyond physical appearance) were identified.

**Intervention’s key messages: April-August 2020**

- Four risk factors for the development of body image concerns were identified: (1) social media and influencers, (2) appearance-based comparisons, (3) appearance-based teasing, and (4) body talk.
- It was decided to reinforce the positive body image traits throughout the intervention identified during the aforementioned focus groups.

**Mode and format of delivery: April-August 2020**

- Storytelling was chosen as the mode of information delivery, as narrative health education is proven to be effective [75] and engaging, even among those with low health literacy [76]. (See Multimedia Appendix 3 for a synopsis of the intervention’s narrative).
- Videos were chosen as the intervention format to deliver the core messages owing to their efficiency in delivering content information (see Media Richness Theory [77]) and ability to pique an audience’s attention and interest [78].
- Interactive activities to reinforce the key messages learned during the videos were added to elicit cognitive dissonance, an effective change technique seen throughout the body image intervention literature [79,80].

**Concept creation: August 2020-January 2021**

- Two storyboard concepts were considered: time travel and a detective-based storyline. Time travel was selected given the ability of this concept to convey the additive impact of body image concerns to young people.
- A combination of animated characters and real people was used, as cartoon characters in health intervention studies have been shown to be acceptable to younger and older adolescents [81-83], as is the combined use of cartoon characters and real people [84].
- The plot and story details were drafted and refined over several collaborative discussions among the various project stakeholders.
- Body image change techniques (including those based on psychoeducation and media literacy [54]) were embedded within the video narrative.
- Six time travel–based videos (ie, one for each target risk factor, plus introductory and concluding videos) were drafted using storyboards and basic animation.

**U-Report poll on appearance-based teasing: January 2021**

- A second U-Report poll with 240 young women was conducted to provide clarity as to how appearance-based teasing presents among young people in Indonesia.
- Poll results provided direction on how to address appearance-based teasing, showing that it is prevalent both online and face-to-face, and that teasing toward young women usually comes from other women, either from friends or family members [85].

**Cocreation of videos with the target audience: February 2021**

- Findings on rough animated versions of the videos from four online focus groups with young women aged 15-19 years (N=16) showed strong comprehension, acceptability, and enjoyment of the videos.
- The young women provided direction regarding appropriate terminology to aid comprehension within their age group.

**Casting, scripts, props, sets, filming: February-March 2021**

- Detailed scripts were written, casting auditions held, sets and props sourced, and decisions on futuristic details such as makeup and style were decided.
- Accurate representation of the diverse Indonesian population through the actors and animated characters involved ensuring that various ethnicities, skin shades, hair textures, distinct types of religious dress, body sizes, and regional accents were included.
- The videos were filmed. Numerous alternative versions were shot for scenes considered potentially problematic for comprehension so that options were available to explore with young women, if necessary.

**Development of activities: February-April 2021**

- Activities were developed to allow users to practice the information learned and/or to reflect on their own cognitions and behaviors in light of the video content.
- The team was guided by Girl Effect and Percolate’s prior experience of disseminating similar interactive content for other health campaigns, as well as the body image scholars’ expertise in effective change techniques.
- Eliciting cognitive dissonance throughout the activities was prioritized, for example, by asking users to speak out against appearance ideals, challenge body talk, and stand up to those bullying based on appearance.

**Acceptability testing of the complete intervention: April 2021**

- Six online focus groups of young women aged 15-19 years (N=36) were conducted to assess intervention acceptability and comprehension, revealing strong acceptability, comprehension, and enjoyment of the videos and activities (see the Intervention Acceptability section).
- No issues with comprehension were identified, and thus no alternative scenes were required.

**Final intervention edits: May 2021**

- Based on focus group feedback, minor edits were made to the instructions for the activities, which focused on making the tone less formal and enhancing comprehension.

Although the majority of the activities are short, four activities are more in-depth, which consist of writing up to 250 words expressively and/or self-compassionately about what they learned in the video, titled “Your Own Words.” These activities were deemed important for participants to complete given the emerging evidence that expressive and compassion-based writing are effective at reducing body dissatisfaction across a range of settings [86,87]. As such, all participants in the trial that complete these activities will be entered into a competition to win Rp50,000 (approximately US $3.50) in mobile phone credit. The responses to this activity will be judged by two authors (KN and BM). Judging will be based upon the best response being (1) relevant, (2) of adequate length, (3) original, (4) potentially inspirational to other young women, and (4) sounding sincere/honest. This approach reflects how the intervention will be disseminated on social media, should the trial find it to be effective (ie, girls who engage with the intervention once disseminated will have the opportunity to submit their response to the writing task for the chance to win Rp50,000 in phone credit). This is a tried-and-tested method to improve engagement in activities that Girl Effect has used in previous health campaigns on social media.

### Intervention Acceptability

In April 2021, the intervention underwent acceptability testing with 36 young women in six focus groups. All groups watched the entire series; however, to minimize participant burden, three groups completed the activities associated with Videos 1-3 and three groups completed the activities for Videos 4-6, providing acceptability feedback on each. The videos scored high on likeability: scores across the videos ranged from 4.44 to 5 (SD 0-0.98) on a 5-point Likert-type scale ranging from 1 (hated the video) to 5 (loved the video). Only one respondent reported not fully understanding one of the videos (Video Five: Be Your Own Best Friend); all other respondents reported full understanding of each video. This finding was corroborated with qualitative feedback from young women, whereby many successfully restated the key messages from each video.

Similar positive findings were found for each of the activities. Across all activities, likeability scores ranged from 4.42 to 4.89 (SD 0.32-0.90) on a 5-point Likert-type scale ranging from 1 (hated the activity) to 5 (loved the activity). The activities were understood by almost all the young women; 94.44%-100% of respondents reported fully understanding each activity. Again, comprehension was further identified through examination of the young women’s qualitative responses to the activities, with the majority of participants responding in a way that exemplified the key messages learned. The authors were particularly keen to understand the likelihood of the target audience to complete the longer Your Own Words tasks. Findings showed that 95% of respondents reported that if they had the time, they would be interested in participating in these tasks.

Based on the acceptability findings, minor edits were made to the reinforcer activity instructions for greater clarity and comprehension. In addition, upon suggestion from the young women, less formal language was adopted to improve engagement. No changes were made to the design or format of the activities.

### Study Setting

The web-based trial will be coordinated by a research agency based in Jakarta, Indonesia. The aim is to recruit young women from across 10 of the largest cities in Indonesia: Balikpapan, Bandung, Jakarta, Makassar, Manado, Medan, Palembang, Pontianak, Semarang, and Surabaya.

### Eligibility Criteria

Inclusion criteria for participation include identifying as a young woman aged between 15 and 19 years (the target age group for the intervention); having their own mobile phone (to ensure participants receive the WhatsApp notifications regarding the study); and accessing Facebook or Instagram daily (so that the sample consists of those who are most likely to access and engage with the intervention when it is disseminated via social media channels in the future). Exclusion criteria include already following the Girl Effect brand (Springster) on any social media site or having ever accessed the Springster website prior to enrolment (to avoid contamination effects); and, if under 18 years of age, not having written consent from a parent or guardian.

### Participant Recruitment and Procedure

**Figure 3:**
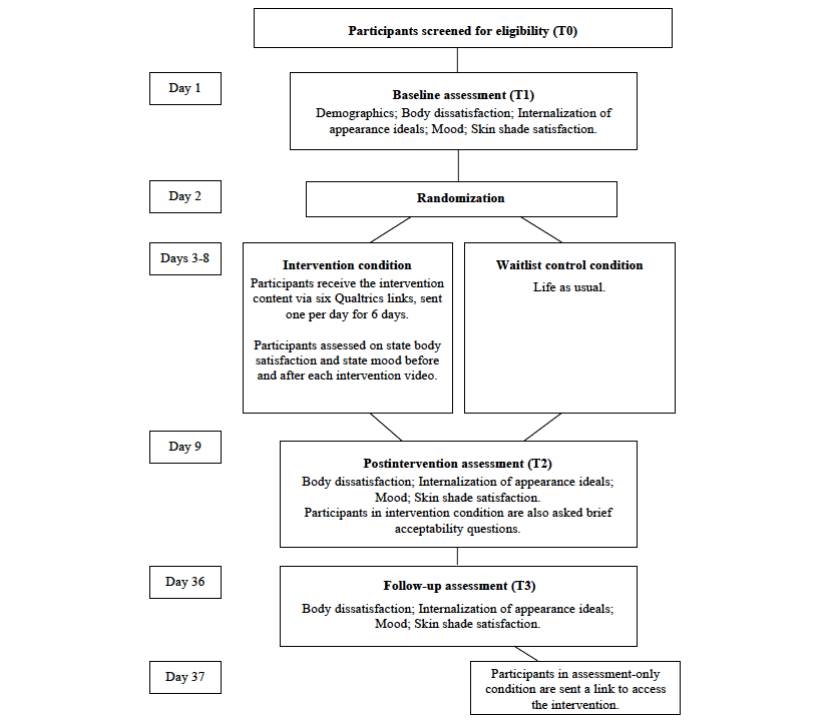
Participant flowchart.

The aim is to recruit 1800 young women aged 15-19 years via a local research agency’s recruitment panel (ie, a database containing contact details of those who have previously taken part in research conducted by the agency and have agreed to be contacted with regard to future research), with the aim to recruit an equal number of participants from each age (ie, 15, 16, 17, 18, and 19 years of age). The CONSORT (Consolidated Standards of Reporting Trials) flowchart is provided in Figure 3. Women and men over the age of 40 years will be contacted via telephone and screened for whether they have a daughter within the eligible age range. If the respondent has more than one daughter in the age range, only one will be eligible to avoid possible contagion effects. The daughter who is the best fit in terms of reaching the age quota will be selected. If this does not distinguish which daughter is selected, the daughter with the birth date closest to the date of contact will be selected. If the respondent does not have a daughter between 15 and 19 years of age, the recruiter will enquire if they know another family with a daughter of this age. If so, the recruiter will request the telephone number of that family and contact them. Only one phone number will be requested per call.

Should an eligible daughter be 15-17 years old, the recruiter will read the parental information sheet to the parent. Parents will then be requested to provide verbal consent for their daughter’s participation and verify their identity and daughter’s age. Parents will then answer questions relating to their socioeconomic status on behalf of their daughter before the recruiter requests to speak to the daughter. If the daughter is not present, the recruiter will request a call back. The daughter will then be screened for eligibility and informed verbal assent obtained. Following the call, and provided the daughter is eligible and gives verbal assent, the parental information sheet will be sent to the parent via WhatsApp, with informed parental consent obtained once more, this time written, over WhatsApp. WhatsApp was chosen as it is the most used communication app in Indonesia [88].

Should an eligible daughter be aged 18 or 19 years, a similar pattern of communication will occur. Parents will verify their own and their daughter’s identity and respond to questions regarding the family’s socioeconomic status. Rather than parents providing informed verbal and written consent, this will be completed by the daughters themselves, in the same manner with which it will be completed by parents of those aged 15-17 years. Verification of identities and ages will be achieved through video calls via the presentation of official documentation (eg, National ID card, family registration card, driving license, student ID). Recruitment is anticipated to take 15 days (inclusive of weekends).

All participants will enter the study (ie, complete the baseline assessment survey) on the same day (Day 1). Participants will receive a data package a day prior to this to ensure they have ample mobile phone data to allow them to participate in the study. A link to the baseline assessment hosted on Qualtrics (Provo, UT) will be sent to participants via WhatsApp at 8 AM (UTC +7) on Day 1, along with a unique participant identification number (PIN). Participants will be requested to enter their PIN on the first page of the baseline survey, in order to match participant responses over time. Participants will have 24 hours to complete the baseline assessment; those who have not completed the baseline assessment within the first 8 hours will be sent a reminder message during the early evening of Day 1. Following the 24-hour window, participants who have completed the baseline assessment will be randomized into one of two conditions: the intervention condition or a waitlist control condition. Participants will be alerted on Day 2 to what happens next, depending on which condition they have been randomized. Those randomized to the intervention condition will be informed that they can expect a series of sequential links to be sent to them daily over the following 6 days. Each link will contain one episode of *Warna-Warni Waktu* and its associated activities. The links will again direct participants to Qualtrics, where the intervention has been embedded (see Multimedia Appendix 2). These participants will be requested to engage with the content of those links daily. Those in the control condition will be told that they will be recontacted in 1 week to complete a second assessment.

On the morning of the third day (Day 3), participants in the intervention condition will be sent their PIN and a link to the first video (and associated activity). Again, participants will be requested to enter their PIN on the first page of the link. Participants will complete state measures of body satisfaction and mood before watching the first video in the *Warna-Warni Waktu* series. State measures of body satisfaction and mood will be asked again immediately after the first video. Next, participants will be presented with the activity for the first video. Before exiting the link, participants will be asked to report on the strength of their internet connectivity while watching the video (see the Measures section below). This process is repeated on Days 4-8 for the remaining five videos (and associated activities). Participants will not be sent reminders to view or engage with the content in these links.

On Day 9 of the study (again, in the morning), participants in both conditions will be sent a link to complete the second assessment. As with the baseline assessment, participants will be given 24 hours to complete this assessment, with reminder messages sent to noncompleters after the first 8 hours. The same process will be executed for the third and final assessment, 1 month later on Day 36. Following the third assessment, all participants will be debriefed on the study aims and provided with additional sources of mental health support, as well as a certificate of participation. Participants who complete all three assessments will be rewarded with Rp125,000 (approximately US $8.75) to encourage participant retention. By this point, the responses to the four 250-word writing activities will have been judged, and those who have won will be contacted and will receive their prize. Those in the waitlist control condition will be provided with a link to the *Warna-Warni Waktu* video series on Day 37. Participants in the waitlist control condition will not be assessed for their engagement with the content. Information sheets and the participant debrief document can be found in Multimedia Appendix 4.

### Randomization and Blinding

Participants will be randomized following completion of the baseline survey. Participants will not be blinded due to the nature of the trial design. They will not be told explicitly of their condition but will be made aware on Day 2 (ie, the day after completing the baseline survey) when they will receive the intervention. Randomization and allocation will be performed on an individual level using an automated, web-based randomizer to sequentially allocate participants based on a block design to ensure a balance of participants across conditions. A researcher external to the project will generate the allocation sequence and assign participants to conditions. They will be blinded to participant information and conditions (ie, they will be given a list of participant identification numbers and asked to assign them to Group A or B using the web-based randomizer). Data analysts will be blinded to condition throughout the trial and analysis of the primary and secondary trait-based measures. Blinding of data analysts during state-based analyses is not possible due to the within-group design of this aspect of the trial. Two separate data files will be provided to the statistician to ensure that blinding during analyses of trait measures is retained. Due to the trial design, the research agency and participants will not be blinded to condition.

### Measures

Self-report measures selected for use are presented in Table 1. For hypothesis one, the primary outcome measure, the Body Esteem Scale for Adults and Adolescents (BESAA) [89], was selected as it is the only body dissatisfaction measure to have been validated among young people in Indonesia (unpublished data of the authors KG, NC, SH, KN, BM, LS, and PD, along with statistical consultants Chloe Hayes and Silia Vitoratou, 2021) and evidence that this scale is amendable to change following interventions of similar duration and content [90,91]. Two secondary outcome measures assessing internalization of appearance ideals [92] and trait mood [93] (unpublished data of the authors KG, NC, SH, KN, BM, LS, and PD, along with statistical consultants Chloe Hayes and Silia Vitoratou, 2021) were again selected for similar reasoning. Owing to the lack of a validated assessment tool to assess skin shade satisfaction, a purpose-built measure will be used to assess this. For hypothesis two, single-item measures of body satisfaction and mood will be utilized. Full questionnaires are presented in Multimedia Appendix 5.

Intervention adherence will be measured objectively through dwell time spent on the Qualtrics page that contains the video and via participant responses to the activities. Intervention acceptability will be assessed through six self-report items; these were informed by established acceptability frameworks [96].

**Table 1:** Measures.

Measure		Description	Time assessed
Demographics		Age, country of birth, ethnicity, religion, socioeconomic status, social media usage	T1^a^
Primary outcome: Trait body dissatisfaction		Body Esteem Scale for Adolescents & Adults (BESAA) [89] adapted and validated among adolescents in Indonesia (17 items). Example item: *I like how I look like in photos.* Response options range from strongly disagree to strongly agree. Mean scores range between 1 and 5, with higher scores reflecting higher body esteem.	T1, T2^b^, T3^c^
**Secondary outcomes**			
	Internalization of appearance ideals	Internalization-General subscale of the Sociocultural Attitudes Towards Appearance Scale-3 [92] adapted and validated among adolescents in Indonesia (12 items). Example item: *I compare my body to the bodies of people who are on TV*. Response options range from *strongly disagree* to *strongly agree*. Mean scores range between 1 and 5, with higher scores reflecting higher internalization of appearance ideals.	T1, T2, T3
	Trait mood	The Positive and Negative Affect Schedule for Children [93] adapted for Indonesia. The validation of this scale among Indonesian adolescents is underway. The factor structure will be determined prior to data analysis and reported in the main effectiveness paper.	T1, T2, T3
	Skin shade satisfaction	A purpose-built, single-item, skin shade discrepancy measure will assess skin shade satisfaction. The chart comprises nine skin colors from dark (1) to light (9). Participants are asked to select the shade that most accurately matches their “current skin shade” and the shade that most accurately reflects the skin shade they would prefer (their “ideal skin shade”). The discrepancy between the two will be used as an indicator of skin shade satisfaction, with higher absolute values indicating less skin shade satisfaction. The score ranges from 0 (*satisfied with skin shade*) to 9 (*very dissatisfied with skin shade*). The measure uses colors from The Pantone Skin Tone Guide [94].	T1, T2, T3
	State body satisfaction	A single 101-point visual analog scale [95] will assess the immediate impact of each video on participants’ state body satisfaction (ie, *How satisfied are you with your appearance, right now, in this moment?*). The total score ranges from 0 (*not at all*) to 101 (*very much*). Higher scores reflect greater satisfaction.	Before and after each video
	State mood	A single 101-point visual analog scale [95] will assess the immediate impact of each video on participants’ state mood (ie, *How happy are you, right now, in this moment?*). The total score ranges from 0 (*not at all*) to 101 (*very much*). Higher scores reflect greater positive mood.	Before and after each video
	Intervention adherence	Digital metrics will assess participants’ engagement with the videos and activities, including: (1) percentage of participants who watch each video (determined by the number of participants whose dwell time on the Qualtrics page hosting each video is equal to or longer than the length of the video); (2) percentage of participants who watch all 6 videos (determined by the number of participants whose dwell time on all 6 Qualtrics pages hosting videos is equal to or longer than the length of each video); (3) average number of videos watched; (4) percentage of participants who complete each activity; (5) average number of activities completed; and (6) average length of time spent engaging with the intervention (ie, watching videos and completing activities) over the 6-day period.	Six-day intervention period
	Intervention acceptability	Six items will assess participants’ acceptability of the intervention. Factors include emotive response to the interventions (eg, enjoyment, likability of characters); relevance (eg, age appropriateness, helpfulness, ease of understanding); ease of use (eg, speed and accuracy of responses); and willingness to recommend (eg, how likely the user would recommend the intervention to a friend). Item scores range between 1 (*strongly disagree*) and 5 (*strongly agree*). Higher scores reflect greater acceptability. Only those randomized to the intervention condition will complete these measures.	T2

^a^T1: baseline assessment.

^b^T2: postintervention assessment.

^c^T3: follow-up assessment.

### Sample Size

The predefined primary outcome measure is the BESAA at postintervention assessment (T2) and follow-up assessment (T3). Similar RCTs assessing body dissatisfaction evaluating the same outcome measure reported a range of small to medium standardized effects sizes with Hedges *g* ranging from 0.25 to 0.4 (eg, [89]) with a standardized minimum important clinical difference (MICD) of 0.2 exceeding the minimum detectable difference of the measures. To detect the MICD or larger, our proposed sample size of n=900 per group would provide in excess of 90% power (two-sided α=.05) for between-group differences at either T2 or T3 with or without a Bonferroni correction for multiple time points. This assumes that dropout does not exceed 20% in any one arm, and is valid for any positive correlation between commensurate measures at baseline (T1) with T2 or T1 and T3. The oversampling includes a COVID-19 contingency plan permitting the sample size to drop to 650 per group should external challenges arise, and further downward revisions in sample size with a tradeoff in power under worst-case scenarios.

### Analysis Plan

The intention-to-treat set of participants will form the primary analysis set. An assessment of the impact of missing data on statistical conclusions will be undertaken using sensitivity analyses.

For the first hypothesis, the data will be analyzed on an intention-to-treat basis using a linear mixed model with baseline measures at T1 as a covariate, randomized group as a two-level between-subjects factor, and study phase (T2, T3) as two-level repeated-measures factor. The statistical model will be hierarchically balanced with the three-way interaction between covariate, phase, and randomized group as the generating class. This structure permits an analysis of covariance prior reasoned comparison between randomized arms at T2 and T3, assessing the parallel lines of assumption, homogeneity of variance assumption, and the use of robust estimates, if needed. Changes within the randomized arm between T1 and T2 and between T1 and T3 will be assessed using the paired-samples *t-*test and effect size quantified with 95% CIs. The reliable change index will be used to determine the percentage of participants reliably improving within each arm.

For the second hypothesis, the nested intervention study comprises state evaluation of body satisfaction and mood immediately before and after each daily video. A component impact analysis will consist of a 2 (pre, post) by 6 (Day 3 to Day 8) fully repeated-measures analysis including linear and quadratic trends for time sequence effects, and a main-effects comparison between components on pre and post change scores. It is not inconceivable that relative effects between days are likely to be small. For an assumed standardized effect of Cohen *d*=0.1, a sample size of n=800 would be needed for 80% power. These data have further value in evaluating adherence and would permit a dose-response effect to be included in a planned subset analysis.

For the third hypothesis, the relationship between adherence (count of daily completion in each of Day 3 to Day 8) and outcome (primary and secondary) at each of T2 and T3 will be assessed using linear regression controlling for baseline covariates. The cardinal nature of adherence permits Helmert effect coding (difference effect coding) to be used to estimate cumulative dosage effects. The relationship between daily engagement and outcome (primary and secondary) at T2 and T3 will be assessed using linear regression controlling for baseline covariates. These latter models will code each daily engagement as a dummy variable, and will permit a comparison between engagement in each activity. A full statistical analysis plan will be written and approved by the Trial Management Group prior to study closure.

### Ethics

This study received ethical approval from the Faculty of Medicine Universitas Indonesia (588/UN2.F1/ETIK/PPM.00.002/2021) and the University of the West of England (HAS.21.04.138). Participation in the study will be completely voluntary. For participants under 18 years of age, parents will be approached first and will be required to give their consent for their daughter’s participation. Prior to consent, parents will be provided with a detailed information sheet outlining the requirements of participation, withdrawal procedures, as well as potential risks. When parents provide their consent, participant assent will be obtained, with the details of the study outlined again in a participant information sheet. The same participant information sheet will be given to those participants aged 18 or 19 (without prior parental consent being sought). Participants will be informed that they can withdraw their consent at any point in the research process without needing to give justification. Parental consent and participant assent (or consent if 18 or 19 years old) will be obtained by the research agency.

All information sheets will contain details of two counseling services available to young women in Indonesia if they are experiencing any mental health concerns and require additional support. Further, information sheets will contain the contact details of study author (BM) should they have any concerns relating to the execution of the study. The study is registered with ClinicalTrials.Gov (NCT05023213).

Special ethical consideration was given in light of conducting this research during the COVID-19 pandemic. Although at the time of writing, legal regulations would allow for face-to-face contact between the research agency and participants, it was decided that the research should take place entirely online. Although such a strategy is befitting for the evaluation of a web-based intervention, assurances regarding identity during the recruitment phase required careful consideration. Video calls where parents of potential participants will be required to show official photographic identification will allow for confirmation of participant identities, including age. Owing to the web-based nature of the study, the decision to only include participants who had access to their own mobile phone was made, ensuring that only the recruited participants receive, and respond to, the notifications sent about the study. Anecdotally, the Indonesian-based authors believe that many young women in the target age group own their own mobile phone; therefore, we do not anticipate this impacting the representativeness of the sample obtained. For transparency, participants ineligible due to not owning their own mobile phone will be reported in the main trial.

### Data Monitoring and Management

Data from recruited participants will be downloaded directly from Qualtrics; thus, there is no data entry process to consider. Once downloaded, the data will be confirmed correct by checking that the data values are as expected. Participant responses will be matched over time, and any duplication of responses will be examined, and if found, deleted. No personal details will be requested from participants via Qualtrics, and as such, these data files should be anonymous. However, the files will be screened at the soonest available opportunity to assess for any inadvertent disclosure of personal information in the form of qualitative responses. If identified, this information will be immediately deleted. Downloaded data will be stored on secure university-approved secure cloud storage (ie, OneDrive). When initial screening described above has been completed by two authors (KG and SH), the data file will then be shared with all study authors.

Consent data, containing personal information, will be collected by the research agency. It will be securely stored for 5 years, as stipulated by the ethical committee of Universitas Indonesia. The personal details of consenting parents or participants will not be shared beyond the research agency.

Based on extensive input from young Indonesian women throughout the development of the intervention and the authors’ experience of utilizing similar surveys among young women in Indonesia with no harm identified, no serious adverse effects are expected for this trial. As such, a data monitoring committee was not deemed appropriate or necessary. Should a participant contact any member of the research team regarding any concerns as a consequence of taking part in the research, this will be dealt with promptly. Such incidences will be reported at the earliest opportunity to the first author (KG) by the research agency, discussed in an internal audit, and reported with the study findings.

### Research Dissemination

For the purposes of disseminating to academic audiences, research findings will be published in peer-reviewed journals and presented at international conferences. Further, the findings will be shared via social media, and websites of the study authors and associated affiliations such as the Centre for Appearance Research, Girl Effect, and Percolate Galactic. The findings may also be shared via communication channels of the funder, the Dove Self-Esteem Project. If the intervention is effective, the intervention content will be disseminated to young women aged 15-19 years via social media marketing campaigns on Facebook, Instagram*,* and YouTube.

## Results

The above protocol will undergo an internal pilot with 150 participants to identify and make any final adjustments to the procedure prior to full execution of the trial. Any deviations from the protocol documented in this paper will be explicitly acknowledged in the publication of the trial findings.

Data collected during the internal pilot from beginning recruitment to the T2 survey will constitute interim analyses. Although T3 data will be collected from pilot participants, this will not form the basis of the interim analyses due to time constraints imposed on the project, along with the minimal additional information this follow-up time point would have on the decision to proceed to the main trial. Progression to the main trial, including decisions regarding any modifications to the protocol, will be based on participant retention, intervention adherence, data quality, and preliminary assessment of harm. Predefined criteria for each of these parameters are outlined in Table 2, using a traffic light system (red: major modifications or termination of the trial required; amber: minor modifications to be considered; green: proceed with protocol as is).

The pilot study was completed in September 2021. Following positive results from the pilot trial, recruitment for the main trial began in October 2021. The results from the trial are anticipated to be published by mid-2022.

**Table 2:** Progression criteria.

Criteria	Description	Green	Amber	Red
Participant retention (%)	Participant completion of T1^a^ and T2^b^ surveys	70 or above: continue with main trial	50-69: consult research team to advise on changes to survey administration protocol	Below 50: the main trial will need to reconsider how surveys are administered
Intervention adherence, n (%)	Participants viewing all 6 intervention videos	80 or above: continue with main trial	60-79: consult research team to advise on changes to intervention delivery	Below 60: the main trial will need to reconsider how intervention is delivered
Data quality, n (%)	Accurate completion of survey attention checks	80 or above: continue with main trial	60-79: consult research team on possible changes to survey instructions	Below 60: reconsider survey completion protocol
Assessment of harm	Assessment of change in primary outcome measure in the intervention condition compared to the control condition, between T1 and T2	Relative improvements in intervention condition: continue with main trial	No difference between conditions: continue with main trial	Relative deterioration in intervention condition: consider trial termination

^a^T1: baseline assessment.

^b^T2: postintervention assessment.

## Discussion

eHealth interventions offer an unparalleled opportunity to reach young people with mental health education and support at scale. The acceptability and effectiveness of delivering mental health content digitally among young people in LMICs is increasingly being explored, showing positive results [69,97]. Although interventions specifically designed for dissemination via social media are in their infancy, the widespread reach and popularity of social media among young people, including in Indonesia [48,49], offer an ideal platform for such an endeavor. To our knowledge, this is the first study to evaluate an eHealth intervention aimed to reduce body dissatisfaction among young people in Indonesia. Specifically, *Warna-Warni Waktu* is a video- and activity-based intervention for young women in Indonesia, designed for dissemination via Facebook, Instagram, and YouTube to reduce body dissatisfaction in this demographic.

This protocol outlines an RCT to evaluate the effectiveness of *Warna-Warni Waktu*. The study has a number of strengths, including an adequate sample size to account for attrition (a recurring issue across web-based intervention trials [98]); an objective assessment of adherence (a crucially important consideration in web-based intervention trials [63]); outcome measures validated among young Indonesian women (with the exception of the skin shade satisfaction measure, of which there is no validated measure available and a key future direction for the field); a 4-week follow-up assessment point to detect any continued or delayed effects; and a pilot study.

However, these noteworthy strengths come at a cost. Most notably, the intervention will be accurately created for use on a single-user software platform (Qualtrics) to objectively measure adherence for the trial. Consideration was given to delivering the trial on social media; however, this would have significantly limited individual-level adherence data, even if the research was conducted via a private Facebook group (eg, this would have allowed tracking of still images at the individual level, but not individual-level adherence to watching the videos from start to finish). As such, the approach described in this paper was deemed an appropriate first step in ensuring intervention effectiveness under a somewhat controlled environment where adherence could be tracked. This decision reduces the study’s ecological validity in that it will not be displayed to participants on the same platform as it will be when disseminated. When delivered on social media, there may be the additive effect of collaborative and cooperative learning [99], with young women posting responses publicly, as well as potentially liking and commenting on others’ responses, in contrast to the didactic fashion to be used in the trial. Note that when disseminated, all public responses will be monitored, with any inappropriate or unhelpful comments removed by moderators. This additional element of having the intervention housed on social media will not be evaluated in the trial described here, but future work could look to explore the impact of this additional element.

If found to be effective, *Warna-Warni Waktu* is a relatively inexpensive, scalable public health intervention to reduce body dissatisfaction among young Indonesian women. It could provide a blueprint for the adoption of this intervention format and modality for other countries and cultural contexts. The intervention’s novelty, accessibility, and acceptability among young women are key strengths of the intervention to date; the effectiveness results from this RCT will be invaluable to guide dissemination efforts across social media platforms in Indonesia.

## Appendix

Multimedia Appendix 1 SPIRIT checklist.

Multimedia Appendix 2 Presentation of activities in Qualtrics and Facebook.

Multimedia Appendix 3 Synopsis of the intervention's narrative.

Multimedia Appendix 4 Participant information sheets.

Multimedia Appendix 5 Questionnaires.

## Abbreviations

BESAA: Body Esteem Scale for Adults and Adolescents
CONSORT: Consolidated Standards of Reporting Trials
LMIC: low- and middle-income country
MICD: minimum important clinical differences
PIN: participant identification number
RCT: randomized controlled trial
SPIRIT: Standard Protocol Items: Recommendations for Interventional Trials
T1: baseline assessment
T2: postintervention assessment
T3: follow-up assessment
