# Supplementary material for: A Novel, Scalable Social Media–Based Intervention (“Warna-Warni Waktu”) to Reduce Body Dissatisfaction Among Young Indonesian Women: Protocol for a Parallel Randomized Controlled Trial
Source: JMIR Res Protoc. 2022 Jan 28;11(1):e33596. doi: 10.2196/33596 (PMC8838567; doi:10.2196/33596)
Supplement: Multimedia Appendix 2 [file resprot_v11i1e33596_app2.pdf]

Supplementary File 2: Presentation of Activities in Qualtrics and Facebook

The following illustrates how the activities will be presented in Qualtrics (i.e., during the trial) and on Facebook (i.e., during future dissemination). The activity instructions in Qualtrics have been edited to remove any references to social media, such as "like this post" or "comment below".

Qualtrics	Facebook
Episode Four, Activity One: Write a short response to a negative appearance-based comment received from a friend or family member.	
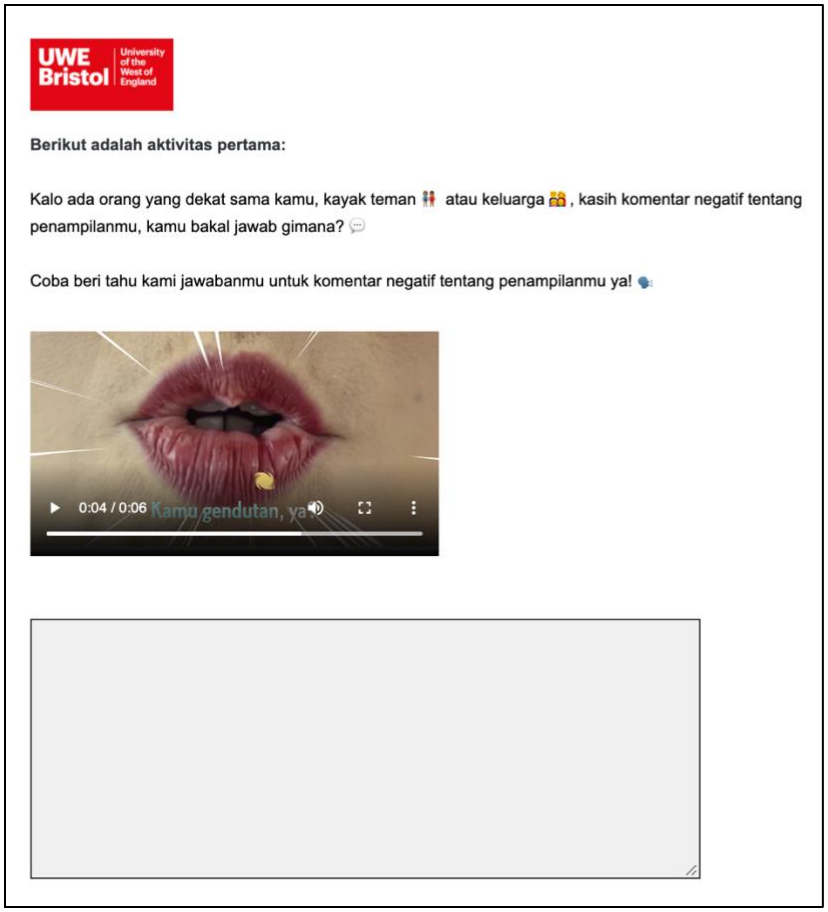	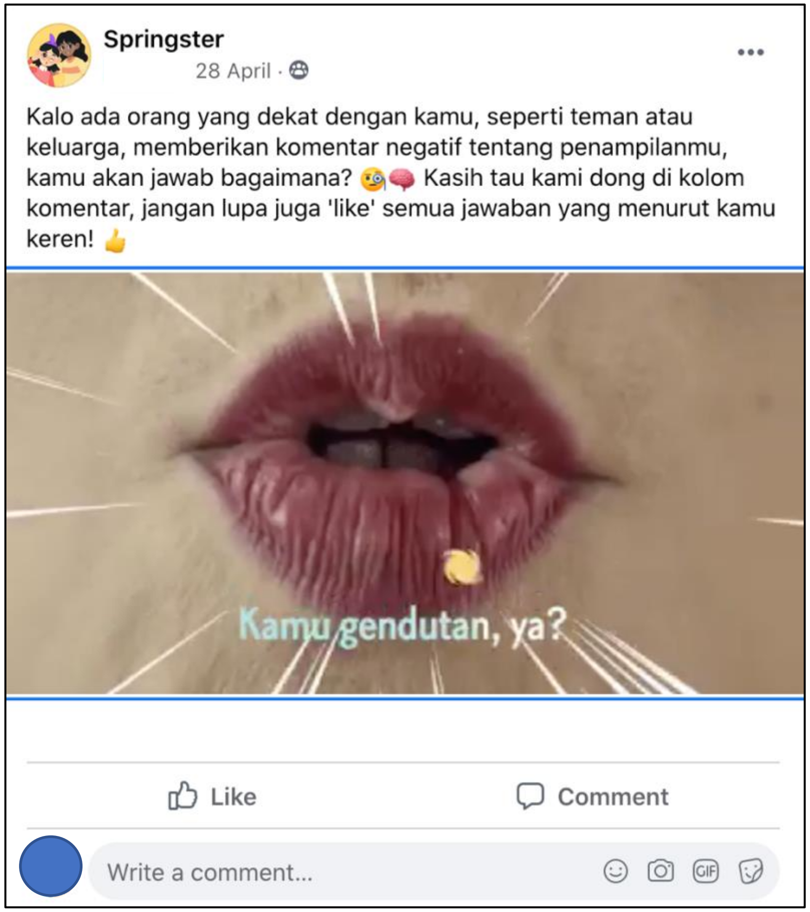

Qualtrics	Facebook
Episode Four, Activity Two: Find 10 hidden example phrases of how to respond to appearance-based comments.	
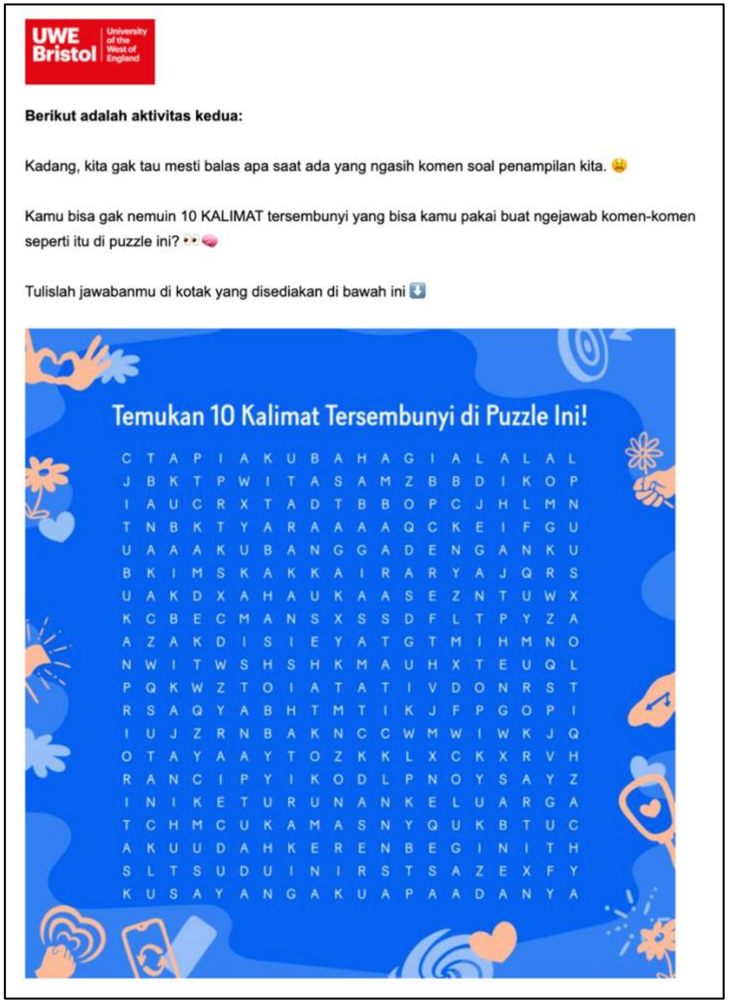	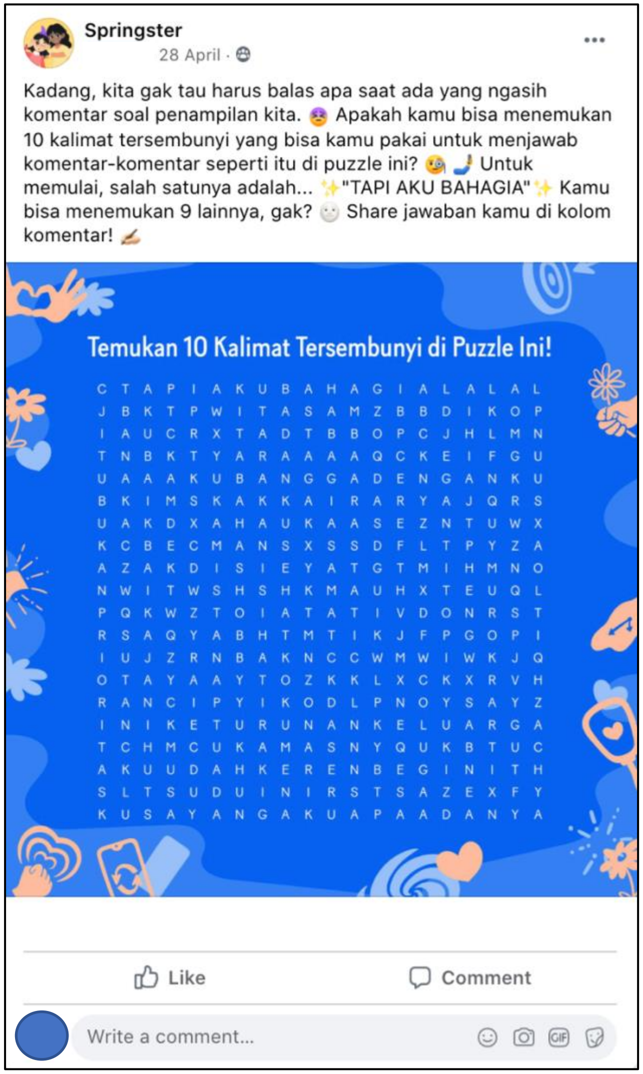

Qualtrics	Facebook
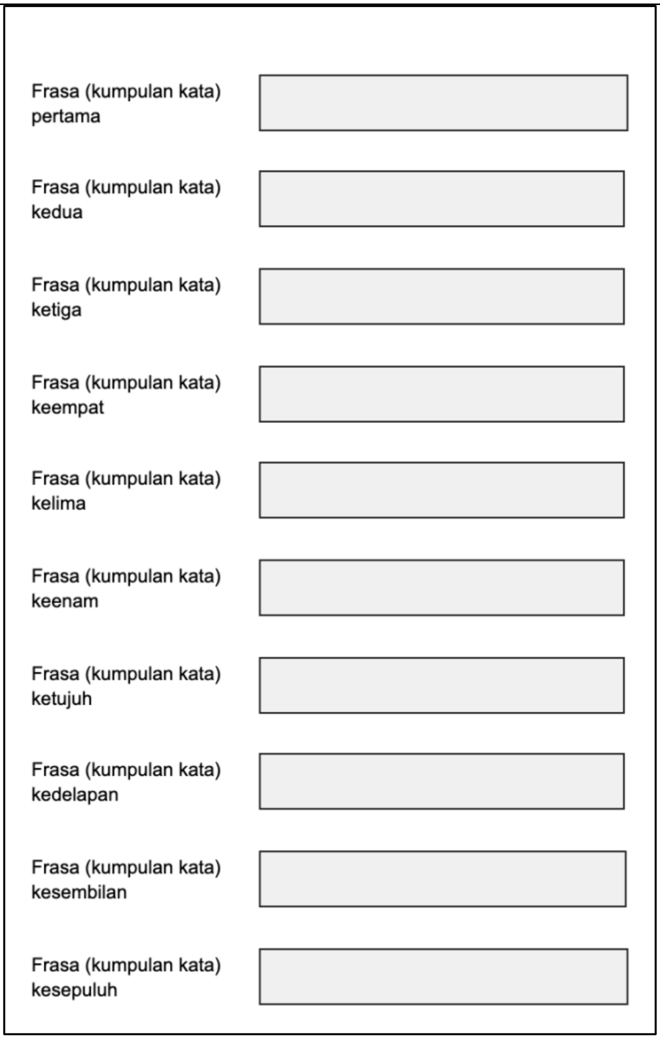<br>Frasa (kumpulan kata) pertama<br>Frasa (kumpulan kata) kedua<br>Frasa (kumpulan kata) ketiga<br>Frasa (kumpulan kata) keempat<br>Frasa (kumpulan kata) kelima<br>Frasa (kumpulan kata) keenam<br>Frasa (kumpulan kata) ketujuh<br>Frasa (kumpulan kata) kedelapan<br>Frasa (kumpulan kata) kesembilan<br>Frasa (kumpulan kata) kesepuluh	

<table>
<tr><th>Qualtrics</th><th>Facebook</th></tr>
<tr><td colspan="2">Episode Four, Activity Three: Complete the comic strip with an imagined response from girls being teased by boys.</td></tr>
<tr><td>

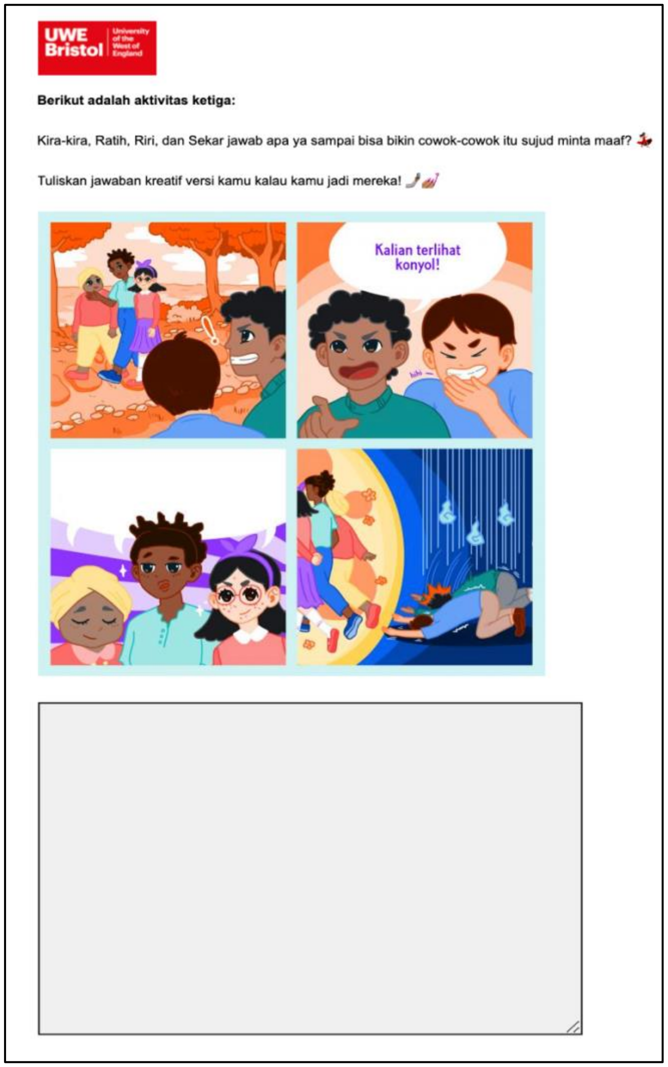

</td><td>

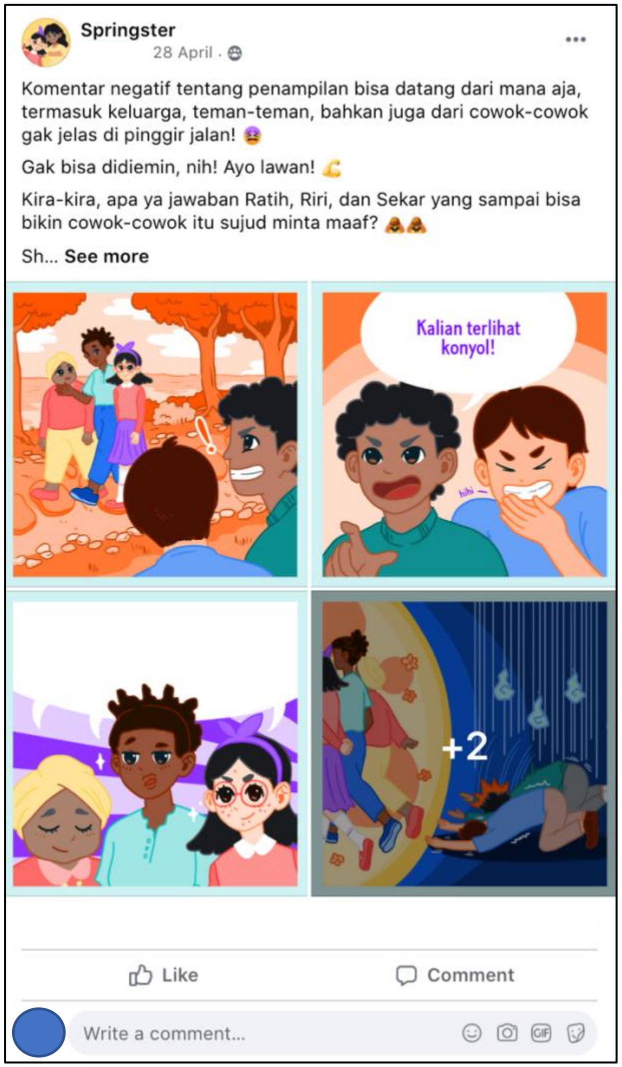

</td></tr>
</table>

Qualtrics	Facebook
Episode Four, Activity Four: Write 250 words about how to stand up to appearance-based comments (Your Own Words competition).	

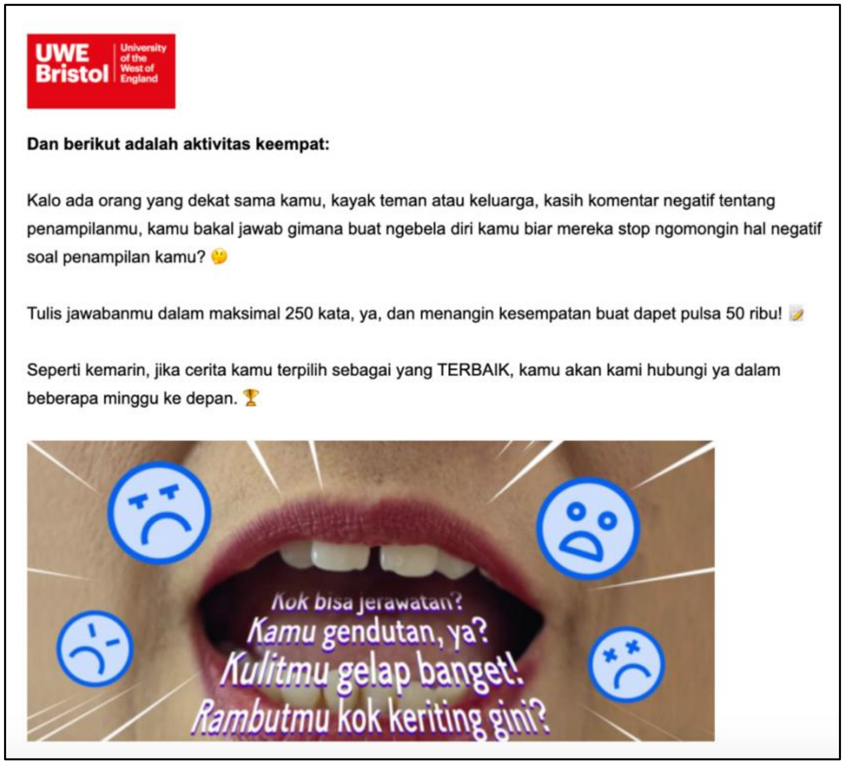

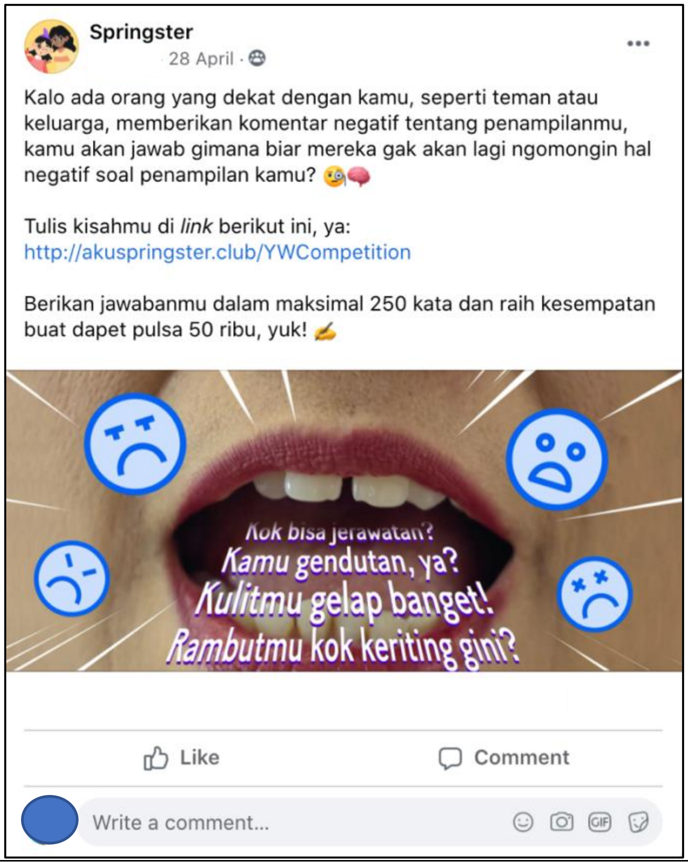

Qualtrics	Facebook
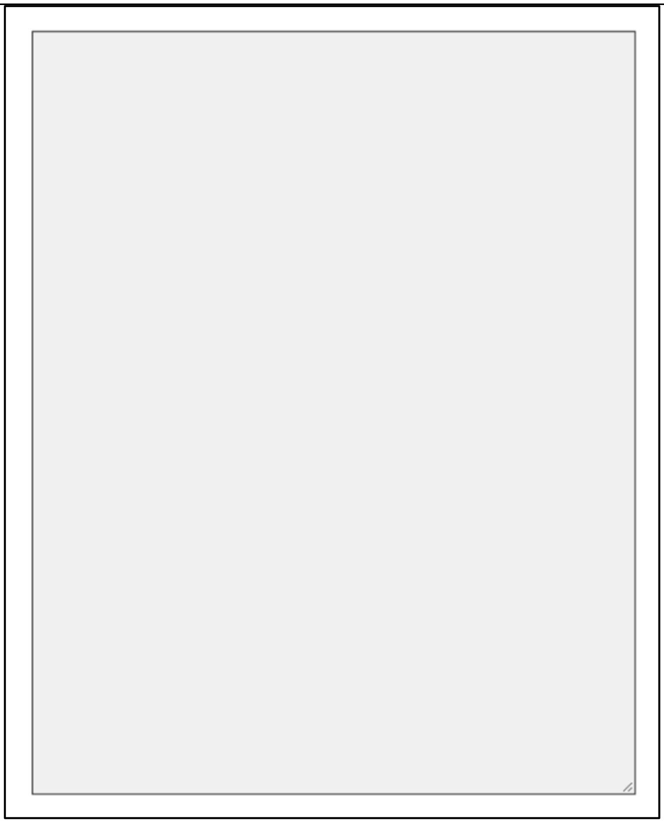	
