# Supplementary material for: A Novel, Scalable Social Media–Based Intervention (“Warna-Warni Waktu”) to Reduce Body Dissatisfaction Among Young Indonesian Women: Protocol for a Parallel Randomized Controlled Trial
Source: JMIR Res Protoc. 2022 Jan 28;11(1):e33596. doi: 10.2196/33596 (PMC8838567; doi:10.2196/33596)
Supplement: Multimedia Appendix 3 [file resprot_v11i1e33596_app3.pdf]

Supplementary File 3: Synopsis of the Intervention's Narrative

Episode	Video synopsis
One: "Time to Turn Back Time"	It's 2033 in Jakarta, and people are faced with intense appearance pressure. Everyone in the video is unhappy. Putri, an everyday Indonesian woman, is working at a powerful company, Cantik Corp (*cantik* means 'beautiful'), which perpetuates narrow and extreme appearance ideals. Five animated time traveler characters, superimposed on the screen, identify Putri as someone who can change the future for the better. The time travelers are able to go back to four moments in Putri's life to help her learn how to challenge appearance pressures, and in turn, save the world from oppressive appearance ideals.
Two: "That's Fake!"	It's 2024, and Putri is a young woman working in an office. She's at her desk, scrolling through social media on her phone. She views a skin lightening product ad that promises instantaneous fairer skin. She purchases, receives, and tries the product. Putri realizes it doesn't work and throws the product into a drawer-full of other beauty products she's wasted her money on. The time travelers intervene by having a transformational before-and-after video appear in her social media feed that teaches her to think critically about what she sees on social media. From this, Putri decides to unfollow harmful beauty brands and takes down and disposes of a poster of a social media influencer.
Three: "C'mon, Break the Chain of Comparisons"	It's 2020, and Putri and her two best friends, Queenza and Kenzo, are at high school together, engaging in appearance comparisons with each other. The time travelers intervene with a cat that is stuck in a tree that needs saving. By working together, they save the cat and recognize that it's because of their specific characteristics, such as Queenza's strength and Kenzo's sharp eyesight, they were able to do something good. The experience helps them recognize and appreciate what their body can do for them, as well as break the circle of comparisons.
Four: "Stand up to Appearance-Based Comments"	It's 2016, and Putri is around 14 years old at an Eid celebration with her family. Putri wants to play with her cousins outside, but her Aunty tells her she needs to stay out of the sun so her skin doesn't get any darker. The time travelers intervene by having a picture of Putri's Aunty playing badminton as a girl crash to the floor. It opens up a discussion between Putri and her Aunty. The conversation sets the scene for Putri to gently help Aunty realize that having fun is more important than one's appearance. Aunty agrees and encourages Putri to play outside with her cousins.

Five:<br>"Be Your Own Best Friend!"	It's 2022, and Putri is a college student. Putri is engaging in negative body talk in front of her bedroom mirror. She wants to skip class because she feels horrible about her appearance. The time travelers intervene by one of them transforming into a person that looks identical to Putri, who speaks to Putri through the mirror. The time traveler helps Putri see that she is bullying herself about her appearance. She has Putri engage in positive self-talk that focuses on body appreciation. Putri feels better about herself and decides to go to class.
Six:<br>"The Color of the Future"	It's 2033 again, but the Jakarta landscape featured in the first episode looks much brighter. Putri is now running Cantik Corp, but it has been renamed Warna Warni Corp, which promotes appearance diversity and self-acceptance. The time travelers reinforce the message that by building body confidence, we can live up to our full potential.
