# Supplementary material for: A Novel, Scalable Social Media–Based Intervention (“Warna-Warni Waktu”) to Reduce Body Dissatisfaction Among Young Indonesian Women: Protocol for a Parallel Randomized Controlled Trial
Source: JMIR Res Protoc. 2022 Jan 28;11(1):e33596. doi: 10.2196/33596 (PMC8838567; doi:10.2196/33596)
Supplement: Multimedia Appendix 4 [file resprot_v11i1e33596_app4.docx]

# **Supplementary File 4. Participant information sheets**

*The following includes the Parental Information Sheet, the Participant Information Sheet, and the Participant Debrief document, all of which will be shared in Bahasa Indonesia.

# **“Warna-Warni Waktu”: Testing an online video series designed to improve Indonesian young women’s body confidence | Parental Information Sheet**

We request your permission to involve your daughter in a research study investigating a series of six short videos titled ‘Warna-Warni Waktu’, an online tool that aims to improve the body confidence of young women in Indonesia. The research study will involve around 2000 young women in Indonesia between the ages of 15 and 19 years. If your daughter is 15, 16, or 17 years of age, it is important for you to understand why the research is being conducted and what it will involve, before you agree to her participation.

The research is being carried out by:

1. **University of Indonesia**
2. **The University of the West of England (The Centre for Appearance Research)**
3. **University of Hawaii**

This project is funded by the **Dove Self-Esteem Project (Unilever)** and is part of a collaboration with **Girl Effect,** a non-profit organisation that seeks to empower girls globally, and **Percolate Galactic**, an Indonesian multi-media company.

The research team is evaluating the effectiveness of a resource to improve the body confidence of young women in Indonesia: ‘Warna-Warni Waktu’, a series of six short videos that tell an entertaining yet educational story of a fictional Indonesian young woman. In addition to the six videos, each video is supplemented with a few short activities designed to help girls think critically about the messages in the videos. All your daughter needs to participate in the research is an internet connection and her own phone.

If you agree for your daughter to take part, she will be asked to:

1. Complete an online survey three times over a six-week period that will ask questions related to body confidence, well-being, and mood. The survey will take approximately 30 minutes to complete.
2. After she has completed the first of the three surveys, she **might** be invited to watch the six videos in the ‘Warna-Warni Waktu’ series, which are approximately 4 minutes long each. If she is invited to watch the video series, she will not watch all six videos at once; they will be shared with her once per day, over six days. After each video, she will be asked to complete a series of short activities and record her in-the-moment feelings before and after watching each video. *If your daughter is not* *selected to receive the videos after the first survey, we will share the videos with her following the third and final survey for her to watch at her leisure.*

By taking part in this study, your daughter will have the opportunity to learn about some important issues concerning the media, friendship, communication, and other influences that can affect young women’s body confidence. Also, the research team will gain a better understanding of how young women in Indonesia feel about themselves and their appearance, which will help us develop resources to support young women in Indonesia improve their body confidence.

Your daughter will be provided with an internet data package to cover the internet usage necessary for participation. Upon completion, your daughter will be issued with a certificate of participation and a money transfer of Rp. 125,000 as a thank-you.

Similar questionnaires to the one that will be used in this study have been widely used by researchers within the Centre for Appearance Research with other girls in this age group in Indonesia. To date, there have been no participants who have exhibited distress after completing these measures.

In the unlikely event that your daughter experiences distress, we provide the contact information of two counselling services (SobatASK and TePSA) at the bottom of the Participant Information Sheet, as well as in the debrief document at the completion of her participation.

The information your daughter gives us will be treated with the highest level of confidentiality. Your daughter will be assigned a unique participation code and her name and identity will never be connected to her responses or included in any sort of report. The information your daughter provides will be anonymised upon completion of the study and stored on a restricted online university repository.

Responses may be written up and the data may be published in an academic journal or elsewhere. Although direct quotes from your daughter may be used in a paper or report, her name and identifying information will be kept anonymous. The data will only be accessible to those working on the project.

The results of the study will be analysed by the research team in the UK and used in reports for the Dove Self-Esteem Project and Girl Effect. The anonymised results may also be used in conference presentations and peer-reviewed academic papers, and in communications by Dove/Unilever and Girl Effect.

The project has been reviewed and approved by the Universitas Indonesia Fakultas Kedokteran (approval number 588/UN2.F1/ETIK/PPM.00.002/2021) and the University of the West of England (approval number HAS.21.04.138). If you have any comments, questions or complaints about the conduct of this study, these can be addressed to:

Dr dr Bernie Medise, Sp.A(K), MPH

Phone: +62 815-8024-517

Email: bernie.medise@yahoo.com

**Contact for Further Information**

If you would like further information about this study or have any other questions, please contact Alexander William Kapawan Azis at Infinity CXT at 08121971766.

**Full details of the research team:** Dr dr Bernie Medise (Indonesian), Pediatrician and Dr Kholisah Nasution (Indonesian), Pediatric Resident at the University of Indonesia; Kirsty Garbett (British), Dr Nadia Craddock (mixed heritage – British & Indian-Malay), Sharon Haywood (Canadian-Irish), and Dr Heidi Williamson (British), Associate Professor at the University of the West of England; and Dr Ayu Saraswati (Indonesian), Associate Professor of Gender Studies at the University of Hawai’i.

**“Warna-Warni Waktu”: Testing an online video series designed to improve Indonesian young women’s body image | Participant Information Sheet**

You are invited to take part in a research study investigating a series of short videos titled ‘Warna-Warni Waktu’, a resource for Indonesian young women that aims to improve body confidence. The research study will involve around 2000 Indonesian young women between the ages of 15 and 19. Before you decide whether to take part, it is important you understand why the research is being conducted and what it will involve.

The research is being carried out by:

1. **University of Indonesia**
2. **The University of the West of England (The Centre for Appearance Research)**
3. **University of Hawaii**

This project is funded by the **Dove Self-Esteem Project (Unilever)** and is part of a collaboration with **Girl Effect,** a global non-profit organisation that seeks to empower girls globally, and **Percolate Galactic**, an Indonesian multi-media company.

The research team is evaluating the effectiveness of ‘Warna-Warni Waktu’, a series of six fictional educational videos. In addition to the six videos, each video is supplemented with a few short activities designed to help girls think critically about the messages in the videos. All you need to participate is an internet connection and your own phone.

If you agree to take part, you will be asked to:

1. Complete an online survey three times over a six-week period that will ask questions related to body confidence, well-being, and mood. The survey will take approximately 30 minutes to complete.
2. After completing the first of the three surveys, you **might** be invited to watch the six videos in the ‘Warna-Warni Waktu’ series, which are approximately 4 minutes long each. If you are invited to watch the video series, they will be shared with you once per day, over six days. After each video, you will be asked to complete a series of short activities and record your in-the-moment feelings before and after watching each video. *If you are not selected to receive the videos after the first survey, we will share the videos with you after you complete the third and final survey. You will then be able to watch the video series whenever you choose.*

It is up to you to decide if you want to be involved. If you decide to take part, ***you are able to withdraw from the research without giving a reason, at any time during the research study period***. You will not be in trouble with the research team if you decide not to take part or withdraw from the study.

If you wish for us to delete your responses during the research study period, you may write to us to request this via WhatsApp. Once your responses have been deleted, we will confirm this via WhatsApp.

By taking part in this research, you will have the opportunity to have your opinions heard in the development of educational resources that the research team hopes will improve the lives of thousands of Indonesian girls.

If you choose to participate, we will provide you with an internet data package to cover the internet usage necessary for your participation. Upon completion, we will issue you with a certificate of participation and a money transfer of Rp 125,000 Rp as a thank-you.

The research team does not anticipate any significant risk to you in taking part in this study; however, we have provided support resources at the bottom of this sheet, should you need them.

The information you give us will be treated with the highest level of confidentiality. You will be assigned a unique participation code, so your name and identity will never be connected to your responses. All the information you provide will be anonymised upon completion of the study and stored on a restricted online university repository.

The research team will write reports and academic papers based on this research. Although direct quotes from you may be used in a report or paper, your name and identifying information will be kept anonymous.

The project has been reviewed and approved by the Universitas Indonesia Fakultas Kedokteran (approval number 588/UN2.F1/ETIK/PPM.00.002/2021) and the University of the West of England (approval number HAS.21.04.138). If you have any comments, questions or complaints about the conduct of this study, these can be addressed to:

Dr dr Bernie Medise, Sp.A(K), MPH

Phone: +62 815-8024-517

Email: [bernie.medise@yahoo.com](mailto:bernie.medise@yahoo.com)

**Contact for Further Information**

If you would like further information about this study or have any other questions, please contact Alexander William Kapawan Azis at Infinity CXT at 08121971766.

# We hope you will be willing to help us with this important research so the resources for Indonesian girls can be the best they can possibly be!

**Related sources available in Indonesia**

SobatASK is an online counselling service, focused on sexual and reproductive health. It is available 24/7: <https://sobatask.net/>.

TePSA is a government run counselling service for all child protection concerns, including sexual abuse and depression or suicidal thoughts. It has nationwide availability, 24 hours a day, 7 days a week. The number to access the services is 021-1500771.

**Full details of the research team:** Dr dr Bernie Medise (Indonesian), Pediatrician and Dr Kholisah Nasution (Indonesian), Pediatric Resident at the University of Indonesia; Kirsty Garbett (British), Dr Nadia Craddock (mixed heritage – British & Indian-Malay), Sharon Haywood (Canadian-Irish), and Dr Heidi Williamson (British), Associate Professor at the University of the West of England; and Dr Ayu Saraswati, Associate Professor of Gender Studies (Indonesian) at the University of Hawai’i.

Thank you for taking part in our research!

You have helped create social media content that will improve the lives of *over half a million* young women in Indonesia!

Below we answer some commonly asked questions about the research project.

**What was this study about?**

Over the past two years a team of like-minded scientists, creative experts and industry leaders have been developing a series of social media posts with the aim of improving body confidence among young Indonesian women. Research shows that many young Indonesian women feel unhappy about their appearance, and we wanted to develop positive content to change that. Pretty cool, huh?

YOU have been involved in a research project that evaluates how effective this social media content is. Has it worked? We hope so! We’ll be examining all the responses we’ve had from our participants over the next couple of months.

**When will this content be available for Indonesian women?**

We will be launching the series early 2022. It might show up on your social media news feed!

**Where can I find out more?**

You can access all the social media content (the Warna-Warni Waktu video series) we’ve been evaluating in this research project here: **[QUALTRICS LINK OF INTERVENTION ONLY]**

For more life and wellbeing tips from the same creators, visit: <https://id.heyspringster.com/>

**Help! I need more support with my body confidence and self-esteem.**

We all need a little extra support sometimes. If you find yourself struggling with your well-being or self-esteem, here are some resources you might find useful.

**SobatASK** is an online counselling service, focused on sexual and reproductive health. It is available 24/7: <https://sobatask.net/>.

**TePSA** is a government run counselling service for all child protection concerns, including sexual abuse and depression or suicidal thoughts. It has nationwide availability, 24 hours a day, 7 days a week. The number to access the services is 021-1500771.
