# Supplementary material for: A Novel, Scalable Social Media–Based Intervention (“Warna-Warni Waktu”) to Reduce Body Dissatisfaction Among Young Indonesian Women: Protocol for a Parallel Randomized Controlled Trial
Source: JMIR Res Protoc. 2022 Jan 28;11(1):e33596. doi: 10.2196/33596 (PMC8838567; doi:10.2196/33596)
Supplement: Multimedia Appendix 5 [file resprot_v11i1e33596_app5.pdf]

Supplementary File 5: Questionnaires
All of the following questionnaires will be shared in Bahasa Indonesia

Time One (T1) Questionnaire

Start of Block: Participant Number

Please enter the Participant Number that the researcher gave you.

____

End of Block: Participant Number / Page Break

Start of Block: Introduction

Welcome to our study, we are so pleased you are here! 👋 If you are under 18 years of age, your parents have provided us with permission for you to participate in our research project. Even if your parents have agreed for you to participate, you can still choose not to – although we really hope that you will choose to take part. 😊

Your participation is required to help us develop resources to improve the self-esteem and confidence of Indonesian girls.

To help you decide if you would like to participate, here is some key information about our research project:

- The research study will occur over a period of **six weeks**.
- During that time, you will be asked to complete **three similar surveys on three different days** about your everyday experiences and feelings about yourself and your body.
- Each survey will take around **20 minutes** to complete.
- We would like you to **complete each of these surveys in one sitting**, but if you need to, you can complete each survey over a period of 24 hours.
- A few days after completing the first survey, you **might** be asked to watch a video of around four minutes in length and complete a few brief activities, **every day for six days in a row**.

If we don't ask you watch videos and complete activities, don't worry! Your opportunity will come to see this content, you'll just have to wait a little bit longer!

----Page Break----

Consent 1

Next, here is some additional important information we want you to know:

1. This is **not a test.** Everyone will have different answers and there are no right or wrong answers.

2. Although it is not a test, it is really important that you **complete your survey on your own, in a quiet space, if you can**. If you have any questions, please ask the researcher who will be happy to help you.

3. All your answers are **completely confidential –** your parents, family and friends won't see them.

4. If you do not feel comfortable answering a particular question, **just skip it** and go on to the next question.

5. **If you do not want to do the survey**, that's OK. Just let the researcher know. You can stop completing the survey at any point, too. Again, just let the researcher know via WhatsApp.

6. If you understand everything we've just told you, **please press "next" to begin**.

----Page break----

Consent 2
Given everything we've told you about this research, do you agree to complete this survey today?

- ○ Yes, let me get started!
- ○ No, thank you for asking.

Skip To: End of Block If Given everything we've told you about this research, do you agree to complete this survey today? = Yes, let me get started!

----Page Break----

Consent check
Are you sure?

- ○ I am sure. I do not want to take part in this study.
- ○ Whoops! I hit the wrong button. I would like to take part in this study today.

Then Branch If:
If Are you sure? = I am sure. I do not want to take part in this study.
Then: End of Survey

End of Block: Introduction / Page Break

Start of Block: Unique Participant Code (UPC)

UPC

Thank you for agreeing to complete the survey!
In order for us to link your response today with the responses you give in future surveys, we need you to create a unique code. This will allow us to match the surveys you complete.

Remember, we won’t ask you to write your name in this survey.

To create your unique code, all you need to do is answer the following two questions:

UPC - First name

1. Write the **first two letters** of your **first name.**
(Example: if your name is ARIF, you would enter AR).

____

UPC - Month of birth
2. Select the **month** you were born in.

○ January
○ February
○ March
○ April
○ May
○ June
○ July
○ August
○ September
○ October
○ November
○ December

End of Block: Unique Participant Code (UPC) / Page Break

Start of Block: Demographics

Age
Before we get started with the survey, please answer the following questions to let us know a little bit about who you are.

What is your age?

- ◯ 15
- ◯ 16
- ◯ 17
- ◯ 18
- ◯ 19

----Page Break----

Indonesian-born?
Were you born in Indonesia?

- ◯ Yes
- ◯ No

----Page Break----

Display This Question:
If Were you born in Indonesia? = No

Country of birth?
In what country were you born?

____

----Page Break----

Ethnicity
What is your ethnicity?

- ◯ Please describe. For example: Javanese, Padang, Sudanese, etc.
____
- ◯ Don't know
- ◯ Prefer not to say

----Page Break----

Religion
What is your religion?

- ◯ Buddhist
- ◯ Hindu
- ◯ Islam
- ◯ Confucianism
- ◯ Christianity – Catholicism
- ◯ Christianity – Protestantism
- ◯ Other (Please describe) ________________________
- ◯ Don't know
- ◯ Prefer not to say

----Page Break----

Internet usage
How often do you use social media?

- ◯ Once a week or less
- ◯ 2-3 times a week
- ◯ 4-5 times a week
- ◯ Every day

----Page Break----

social media
Which social media sites do you use? Please check all that apply.

- ▢ Facebook
- ▢ Twitter
- ▢ Instagram
- ▢ TikTok

- [ ] YouTube
- [ ] SnapChat
- [ ] WhatsApp
- [ ] Snapgram
- [ ] Other (please describe) ____

----Page Break----

Fav social media
Which social media site do you use the MOST?

- ○ Facebook
- ○ Twitter
- ○ Instagram
- ○ TikTok
- ○ YouTube
- ○ SnapChat
- ○ WhatsApp
- ○ Snapgram
- ○ Other ____

End of Block: Demographics / Page Break

Start of Block: Instructions

Instruction intro
Thanks for answering those questions for us 🙏

Next, we're going to ask you some questions about your everyday experiences and feelings about yourself and your body.

Are you sitting comfortably? Here we go! 🚀

----Page Break----

Instruction intro 2

To complete these questions, you will need to select the answer that matches how you think and feel about important areas of your life.

Remember, there are no right or wrong answers. Got it?

Let’s try an example question to get you started.

Click "Next" to start the example question. →

----Page Break----

1st example question

How much do you agree or disagree with the following statement?

I like reading books.

Over to you. Select the response that best describes how you feel about reading books.

- ○ Strongly disagree
- ○ Disagree
- ○ Neither agree nor disagree
- ○ Agree
- ○ Strongly agree

----Page Break----

T1 check1

Easy, right?

- If you *really* like reading books, you would have chosen “strongly agree”.
- If you like reading books most of the time, you would have chosen “agree”.
- If you like reading books sometimes, you maybe would have chosen “neither agree nor disagree”.
- If you don’t like reading books, you would have chosen “disagree”.
- Finally, if you *really don’t* like reading books, you would have chosen “strongly disagree”.

Now, do you feel ready to get started?

- ○ Yes, let me get started.
- ○ No, I'd like to practice with another question.

Skip To: Start If Easy, right? If you really like reading books, you would have chosen “strongly agree”. If y... = Yes, let me get started.

Display This Question:
If Easy, right? If you really like reading books, you would have chosen “strongly agree”. If y... = No, I'd like to practice with another question.

2nd example question
Let’s try another example question.

How much do you agree or disagree with the following statement?

I find it easy to get to sleep at night.

Now, select the response that best describes how you easy you find getting to sleep at night.

- ○ Strongly disagree
- ○ Disagree
- ○ Neither agree nor disagree
- ○ Agree
- ○ Strongly agree

----Page Break----

T1 check 2
That wasn't too difficult, was it?

- If you find you always find it easy to get to sleep at night, you would have chosen “strongly agree”.
- If you find you find it easy to get to sleep at night most of the time, you would have chosen “agree”.
- If you find you find it easy to get to sleep at night sometimes, you maybe would have chosen “neither agree nor disagree”.
- If you don't find it easy to get to sleep at night most of the time, you would have chosen “disagree”.
- Finally, if you never find it easy to get to sleep at night, you would have chosen “strongly disagree”.

Are you ready to get started now?

- ○ Yes, I'm ready to start.
- ○ No, I'm still not sure.

----Page Break----

Display This Question:
If That wasn't too difficult, was it? If you find you always find it easy to get to sleep at night... = No, I'm still not sure.

Extra help required
Please WhatsApp the researcher for further explanation on how to complete the questions.

After speaking with the researcher and are clear on how to complete the questions, please click "Next" to begin. ➡

----Page Break----

Display This Question:
If That wasn't too difficult, was it? If you find you always find it easy to get to sleep at night... = Yes, I'm ready to start.
Or Please WhatsApp the researcher for further explanation on how to complete the questions. After... Is Displayed

Start Let's begin! 🚀 (Remember, the researcher will be available via WhatsApp in case you have any questions.)

End of Block: Instructions / Page Break

Start of Block: UPDATED BESAA

T1 BES Q1,2,4,5
Please select how often each of the following statements is true of you:

T1 BES Q1
1. I like what I look like in pictures (*photos*).

- ○ Never
- ○ Rarely
- ○ Sometimes
- ○ Often
- ○ Always

T1 BES Q2

2. Other people consider me good looking (*beautiful/handsome/pretty*).

- Never
- Rarely
- Sometimes
- Often
- Always

T1 BES Q4

3. I am preoccupied with trying to change my body weight.

- Never
- Rarely
- Sometimes
- Often
- Always

T1 BES Q5

4. I think my appearance would help me get a job.

- Never
- Rarely
- Sometimes
- Often
- Always

----Page Break----

T1 BES Q6,8,10,11

Please select how often each of the following statements is true of you:

T1 BES Q6

5. I like what I see when I look in the mirror.

- ○ Never
- ○ Rarely
- ○ Sometimes
- ○ Often
- ○ Always

T1 BES Q8

6. I am satisfied with my weight.

- ○ Never
- ○ Rarely
- ○ Sometimes
- ○ Often
- ○ Always

T1 BES Q10

7. I really like what I weigh.

- ○ Never
- ○ Rarely
- ○ Sometimes
- ○ Often
- ○ Always

T1 BES Q11

8. I wish I looked like someone else.

- ○ Never
- ○ Rarely
- ○ Sometimes
- ○ Often
- ○ Always

----Page Break----

T1BESQ12,13,14,15,16

Please select how often each of the following statements is true of you:

T1 BES Q12

9. People my own age like my looks.

- ○ Never
- ○ Rarely
- ○ Sometimes
- ○ Often
- ○ Always

T1 BES Q13

10. My looks upset me (sad).

- ○ Never
- ○ Rarely
- ○ Sometimes
- ○ Often
- ○ Always

T1 BES Q14

11. I’m as nice looking as most people.

- ○ Never
- ○ Rarely
- ○ Sometimes
- ○ Often
- ○ Always

T1 BES Q15

12. I’m pretty happy about the way I look.

- ◯ Never
- ◯ Rarely
- ◯ Sometimes
- ◯ Often
- ◯ Always

T1 BES Q16

13. I feel I weigh the right amount for my height.

- ◯ Never
- ◯ Rarely
- ◯ Sometimes
- ◯ Often
- ◯ Always

----Page Break----

T1BESQ17,19,20,21, AC1

Please select how often each of the following statements is true of you:

T1 BES Q17

14. I feel ashamed of how I look.

- ◯ Never
- ◯ Rarely
- ◯ Sometimes
- ◯ Often
- ◯ Always

T1 BES AC1

15. Please choose "rarely"

- ◯ Never
- ◯ Rarely
- ◯ Sometimes
- ◯ Often
- ◯ Always

T1 BES Q19

16. My weight makes me unhappy.

- ◯ Never
- ◯ Rarely
- ◯ Sometimes
- ◯ Often
- ◯ Always

T1 BES Q20

17. My looks make me attractive (beautiful/handsome/pretty) and popular.

- ◯ Never
- ◯ Rarely
- ◯ Sometimes
- ◯ Often
- ◯ Always

T1 BES Q21

18. I worry about the way I look.

- Never
- Rarely
- Sometimes
- Often
- Always

End of Block: UPDATED BESAA / Page Break

Start of Block: Internalisation

T1 INT Q1-4

How much do you agree with the following statements?

T1 INT Q1

1. I would like my body to look like the bodies of people who are on TV.

- Totally disagree
- Mostly disagree
- Neither agree nor disagree
- Mostly agree
- Totally agree

T1 INT Q2

2. I compare my body to the bodies of people who are on TV.

- Totally disagree
- Mostly disagree
- Neither agree nor disagree
- Mostly agree
- Totally agree

T1 INT Q3

3. I would like my body to look like the models who appear in magazines.

- ◯ Totally disagree
- ◯ Mostly disagree
- ◯ Neither agree nor disagree
- ◯ Mostly agree
- ◯ Totally agree

T1 INT Q4

4. I compare my appearance to the appearance of TV and movie stars.

- ◯ Totally disagree
- ◯ Mostly disagree
- ◯ Neither agree nor disagree
- ◯ Mostly agree
- ◯ Totally agree

----Page Break----

T1 INT Q5-8

How much do you agree with the following statements?

T1 INT Q5

5. I would like my body to look like the people who are in movies

- ◯ Totally disagree
- ◯ Mostly disagree
- ◯ Neither agree nor disagree
- ◯ Mostly agree
- ◯ Totally agree

T1 INT Q6

6. I compare my body to the bodies of people who appear in magazines.

- ○ Totally disagree
- ○ Mostly disagree
- ○ Neither agree nor disagree
- ○ Mostly agree
- ○ Totally agree

T1 INT Q7

7. I wish I looked like the models in music videos.

- ○ Totally disagree
- ○ Mostly disagree
- ○ Neither agree nor disagree
- ○ Mostly agree
- ○ Totally agree

T1 INT Q8

8. I compare my appearance to the appearance of people in magazines.

- ○ Totally disagree
- ○ Mostly disagree
- ○ Neither agree nor disagree
- ○ Mostly agree
- ○ Totally agree

----Page Break----

T1 INT Q9-12

How much do you agree with the following statements?

T1 INT Q9

9. I try to look like the people on TV.

- Totally disagree
- Mostly disagree
- Neither agree nor disagree
- Mostly agree
- Totally agree

T1 INT Q10

10. I compare my body to the bodies of people who are on social media.

- Totally disagree
- Mostly disagree
- Neither agree nor disagree
- Mostly agree
- Totally agree

T1 INT Q11

11. I would like my body to look like the bodies of people who are on social media.

- Totally disagree
- Mostly disagree
- Neither agree nor disagree
- Mostly agree
- Totally agree

T1 INT Q12

12. I try to look like the people I see on social media.

- Totally disagree
- Mostly disagree
- Neither agree nor disagree
- Mostly agree
- Totally agree

End of Block: Internalisation / Page Break

Start of Block: Progress note

T1 Progress note

Congratulations! You have completed more than half the survey!

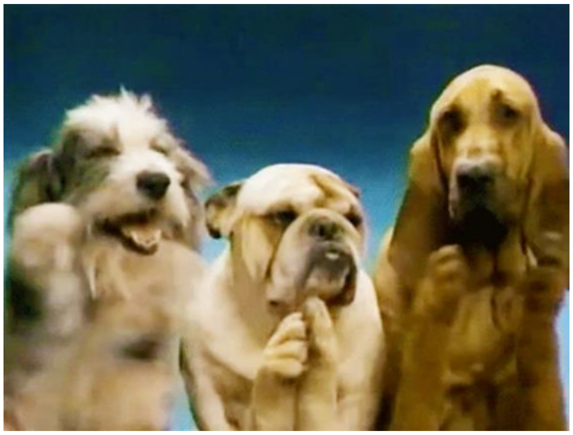

via GIPHY

End of Block: Progress note / Page Break

Start of Block: PANAS

T1 PANAS Q1-6

For each item below, please select the response that best describes how much you have felt that way **over the past week**.

	Never	Rarely	Sometimes	Often	Always
1. **Interested**	○	○	○	○	○
2. **Alert**	○	○	○	○	○
3. **Excited**	○	○	○	○	○
4. **Happy**	○	○	○	○	○
5. **Strong**	○	○	○	○	○
6. **Energetic**	○	○	○	○	○

----Page Break----

T1 PANAS Q7-12

For each item below, please select the response that best describes how much you have felt that way over the past week.

	Never	Rarely	Sometimes	Often	Always
7. **Calm**	○	○	○	○	○
8. **Cheerful**	○	○	○	○	○
9. **Active**	○	○	○	○	○
10. **Proud**	○	○	○	○	○
11. **Joyful**	○	○	○	○	○
12. **Fearless**	○	○	○	○	○

----Page Break----

T1 PANAS Q13-18

For each item below, please select the response that best describes how much you have felt that way over the past week.

	Never	Rarely	Sometimes	Often	Always
13. **Delighted**	○	○	○	○	○
14. **Daring**	○	○	○	○	○
15. **Sad**	○	○	○	○	○
16. **Frightened**	○	○	○	○	○
17. **Ashamed**	○	○	○	○	○
18. **Upset**	○	○	○	○	○

----Page Break----

T1 PANAS Q19-23 +AC2

For each item below, please select the response that best describes how much you have felt that way over the past week.

	Never	Rarely	Sometimes	Often	Always
19. **Nervous**	○	○	○	○	○
20. **Guilty**	○	○	○	○	○
21. **Please choose "often".**	○	○	○	○	○
22. **Scared**	○	○	○	○	○
23. **Miserable**	○	○	○	○	○
24. **Jittery/jumpy**	○	○	○	○	○

T1 PANAS Q24-28
For each item below, please select the response that best describes how much you have felt that way over the past week.

	Never	Rarely	Sometimes	Often	Always
25. **Afraid**	○	○	○	○	○
26. **Lonely**	○	○	○	○	○
27. **Mad**	○	○	○	○	○
28. **Disgusted**	○	○	○	○	○
29. **Gloomy**	○	○	○	○	○

End of Block: PANAS / Page Break

Start of Block: Skin Shade satisfaction

UWE Bristol University of the West of England

T1 Skin Q1

How dissatisfied or satisfied you are with the **colour of your skin?**

- ◯ Very dissatisfied
- ◯ Mostly dissatisfied
- ◯ Neither satisfied nor dissatisfied
- ◯ Mostly satisfied
- ◯ Very satisfied

T1 Skin Q2

Which of the following statements do you **agree** with the most:

- ◯ I would like my skin colour to be lighter
- ◯ I would like my skin colour to stay the same
- ◯ I would like my skin colour to be darker

----Page Break----

T1Skin chart_current

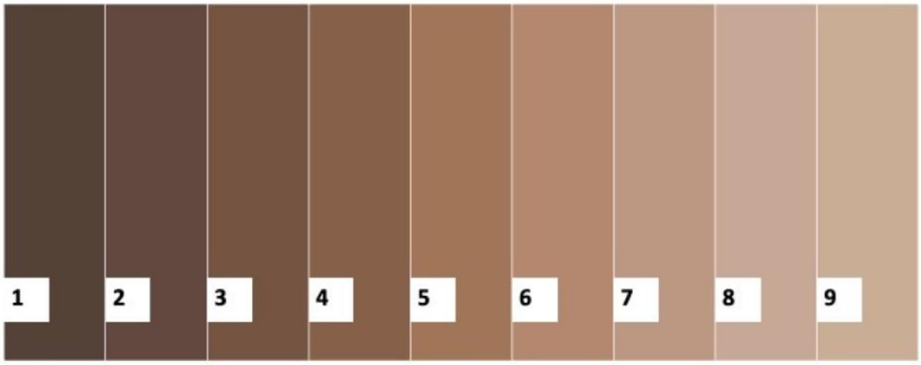

Using the chart above, please select the number that best represents **your current skin shade**.

- 1
- 2
- 3
- 4
- 5
- 6
- 7
- 8
- 9

----Page Break----

T1 Skin chart_ideal

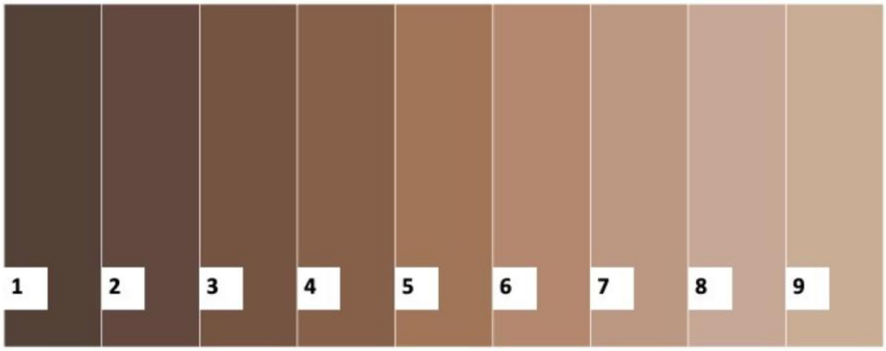

Using the chart above, select the number that best represents **the skin shade that you would like for yourself - your 'ideal' skin shade**.

○ 1
○ 2
○ 3
○ 4
○ 5
○ 6
○ 7
○ 8
○ 9

End of Block: Skin Shade satisfaction / Page Break

Start of Block: Final message

Final

Thank you for your participation! We’ll be sharing two similar surveys with you over the next few weeks. 📅

It’s important you complete them as soon as possible once you receive them, so look out for them! 👀

Many thanks again for helping us with this important work! You are helping to improve the lives of adolescent girls across Indonesia. 🙏

Please click "Next" to close the survey. ➡

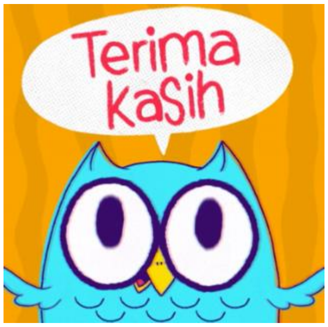

via GIPHY

End of Block: Final message

Thank you for taking the time to complete this survey.
Your response has been recorded.

Time Two (T2) Questionnaire

Start of Block: Participant Number

Participant number
Please enter the Participant Number that the researcher gave you.

____

End of Block: Participant Number / Page Break

Start of Block: Consent

Consent
Today, we’d like to request you complete another survey for us.

Just like the previous survey, it will take around 30 minutes to complete.

○ Yes, let's go!

○ No, thank you for asking.

Display This Question:
If Today, we’d like to request you complete another survey for us. Just like the previous survey, it... = No, thank you for asking.

Consent check
Are you sure?

○ I am sure. I do not want to take part in this study.

○ Whoops! I hit the wrong button. I would like to take part in this study today.

Then Branch If:
If Are you sure? = I am sure. I do not want to take part in this study.
Then: End of Survey

----Page Break----

Display This Question:
If Today, we’d like to request you complete another survey for us. Just like the previous survey, it... = Yes, let's go!
Or Are you sure? = Whoops! I hit the wrong button. I would like to take part in this study today.

Consent reminder
Before you get started, we would like to remind you about some important information about this survey:

1. This is **not a test.** Everyone will have different answers and there are no right or wrong answers.

2. Although it is not a test, it is really important that you **complete your survey on your own, in a quiet space, if you can.** If you have any questions, please ask the researcher who will be happy to help you.

3. All your answers are **completely confidential –** your parents, family and friends won't see them.

4. If you do not feel comfortable answering a particular question, **just skip it** and go on to the next question.

5. **If you do not want to do the survey**, that's OK. Just let the researcher know. You can stop completing the survey at any point, too. Again, just let the researcher know.

6. If you understand everything we've just told you, **please press "next" to begin**.

End of Block: Consent / Page Break

Start of Block: UPC

UPC
✨ **Thank you for agreeing to complete a survey for us again!** ✨ Next, we need you to recreate your unique code that you created 7-10 days ago. This will enable us to link what you tell us today to what you told us the first time you completed a survey for us. As with the first survey, please answer the following two questions:

UPC - First name
1. Write the **first two letters** of your **first name.**
(Example: if your name is ARIF, you would enter AR).

____

UPC - Month of birth
2. Select the **month** you were born in.

- ○ January
- ○ February
- ○ March

April

May

June

July

August

September

October

November

December

End of Block: UPC / Page Break

Start of Block: Instructions

Instruction refresh?
Would you like a refresher on how to complete the questions in the survey?

Yes, please!

No, I remember what I need to do.

Skip To: Start If Would you like a refresher on how to complete the questions in the survey? = No, I remember what I need to do.

Display This Question:
If Would you like a refresher on how to complete the questions in the survey? = Yes, please!

T2 Example intro
As before, this survey asks questions about some of your everyday experiences and feelings about yourself and your body.

You will need to select the answer that matches how you think and feel about important areas of your life. Remember, there are no right or wrong answers.

Let's try an example. Click "Next" to start the example question. →

T2 1st example
How much do you agree or disagree with the following statement?

I like playing sports.

Over to you. Select the response that best describes how you feel about playing sports.

- ○ Strongly disagree
- ○ Disagree
- ○ Neither agree nor disagree
- ○ Agree
- ○ Strongly agree

----Page Break----

T2 Check 1
Not too difficult, right?

- If you **really like** playing sports, you would have chosen “strongly agree”.
- If you like playing sports **most of the time**, you would have chosen “agree”.
- If you like playing sports **sometimes**, you would have chosen “neither agree nor disagree”.
- If you **don’t** like playing sports, you would have chosen “disagree”.
- Finally, if you **really don’t** like playing sports, you would have chosen “strongly disagree”.

Do you feel ready to get started?

- ○ Yes, let me get started.
- ○ No, I'd like another practice question.

Skip To: Start If Not too difficult, right? If you really like playing sports, you would have chosen “strongly a... = Yes, let me get started.

Display This Question:
If Not too difficult, right? If you really like playing sports, you would have chosen “strongly a... = No, I'd like another practice question.

T2 2nd example
Let’s try another example question.

How much do you agree or disagree with the following statement?

I am a kind person.

Now, select the response that best describes how you feel about being a kind person.

- ○ Strongly disagree
- ○ Disagree
- ○ Neither agree nor disagree
- ○ Agree
- ○ Strongly agree

----Page Break----

T2 Check 2

- If you think you are always a kind person, you would have chosen “strongly agree”.
- If you think you are a kind person most of the time, you would have chosen “agree”.
- If you think you are a kind person sometimes, you maybe would have chosen “neither agree nor disagree”.
- If you don't think you are a kind person most of the time, you would have chosen “disagree”.
- Finally, if you think you are never a kind person, you would have chosen “strongly disagree”.

Now, are you ready to get started?

- ○ Yes, let me get started.
- ○ No, I'm still not sure.

Skip To: Start If If you think you are always a kind person, you would have chosen “strongly agree”. If you thin... = Yes, let me get started.

Display This Question:
If If you think you are always a kind person, you would have chosen “strongly agree”. If you thin... = No, I'm still not sure.

Extra help required
Please WhatsApp the researcher for further explanation on how to complete the questions.

After speaking with the researcher and are clear on how to complete the questions, please click "Next" to begin. →

Display This Question:
If If you think you are always a kind person, you would have chosen “strongly agree”. If you thin... = Yes, let me get started.
Or Not too difficult, right? If you really like playing sports, you would have chosen “strongly a... = Yes, let me get started.
Or Please WhatsApp the researcher for further explanation on how to complete the questions. After... Is Displayed
Or Would you like a refresher on how to complete the questions in the survey? = No, I remember what I need to do.

End of Block: Instructions / Page Break

Start of Block: UPDATED BESAA

T2 BES Q1,2,4,5
Please select how often each of the following statements is true of you:

T2 BES Q1
1. I like what I look like in pictures (*photos*).

- ○ Never
- ○ Rarely
- ○ Sometimes
- ○ Often
- ○ Always

T2 BES Q2
2. Other people consider me good looking (*beautiful/handsome/pretty*).

- ○ Never
- ○ Rarely
- ○ Sometimes
- ○ Often
- ○ Always

T2 BES Q4

3. I am preoccupied with trying to change my body weight.

- ○ Never
- ○ Rarely
- ○ Sometimes
- ○ Often
- ○ Always

T2 BES Q5

4. I think my appearance would help me get a job.

- ○ Never
- ○ Rarely
- ○ Sometimes
- ○ Often
- ○ Always

----Page Break----

T2 BES Q6,8,10,11

Please select how often each of the following statements is true of you:

T2 BES Q6

5. I like what I see when I look in the mirror.

- ○ Never
- ○ Rarely
- ○ Sometimes
- ○ Often
- ○ Always

UWE Bristol University of the West of England

T2 BES Q8

6. I am satisfied with my weight.

- ◯ Never
- ◯ Rarely
- ◯ Sometimes
- ◯ Often
- ◯ Always

T2 BES Q10

7. I really like what I weigh.

- ◯ Never
- ◯ Rarely
- ◯ Sometimes
- ◯ Often
- ◯ Always

T2 BES Q11

8. I wish I looked like someone else.

- ◯ Never
- ◯ Rarely
- ◯ Sometimes
- ◯ Often
- ◯ Always

----Page Break----

T2BESQ12,13,14,15,16

Please select how often each of the following statements is <u>true of you</u>:

T2 BES Q12

9. People my own age like my looks.

- Never
- Rarely
- Sometimes
- Often
- Always

T2 BES Q13

10. My looks upset me (sad).

- Never
- Rarely
- Sometimes
- Often
- Always

T2 BES Q14

11. I'm as nice looking as most people.

- Never
- Rarely
- Sometimes
- Often
- Always

T2 BES Q15

12. I'm pretty happy about the way I look.

- Never
- Rarely
- Sometimes
- Often
- Always

T2 BES Q16

13. I feel I weigh the right amount for my height.

- Never
- Rarely
- Sometimes
- Often
- Always

----Page Break----

T2BESQ17,19,20,21,AC

Please select how often each of the following statements is true of you:

T2 BES Q17

14. I feel ashamed of how I look.

- Never
- Rarely
- Sometimes
- Often
- Always

T2 BES AC1

15. Please choose "often".

- Never
- Rarely
- Sometimes
- Often
- Always

T2 BES Q19
16. My weight makes me unhappy.

- ◯ Never
- ◯ Rarely
- ◯ Sometimes
- ◯ Often
- ◯ Always

T2 BES Q20
17. My looks make me attractive (beautiful/handsome/pretty) and popular.

- ◯ Never
- ◯ Rarely
- ◯ Sometimes
- ◯ Often
- ◯ Always

T2 BES Q21
18. I worry about the way I look.

- ◯ Never
- ◯ Rarely
- ◯ Sometimes
- ◯ Often
- ◯ Always

End of Block: UPDATED BESAA / Page Break

Start of Block: Internalisation

T2 INT Q1-4
How much do you agree with the following statements?

T2 INT Q1

1. I would like my body to look like the bodies of people who are on TV.

- ◯ Totally disagree
- ◯ Mostly disagree
- ◯ Neither agree nor disagree
- ◯ Mostly agree
- ◯ Totally agree

T2 INT Q2

2. I compare my body to the bodies of people who are on TV.

- ◯ Totally disagree
- ◯ Mostly disagree
- ◯ Neither agree nor disagree
- ◯ Mostly agree
- ◯ Totally agree

T2 INT Q3

3. I would like my body to look like the models who appear in magazines.

- ◯ Totally disagree
- ◯ Mostly disagree
- ◯ Neither agree nor disagree
- ◯ Mostly agree
- ◯ Totally agree

T2 INT Q4

4. I compare my appearance to the appearance of TV and movie stars.

- ○ Totally disagree
- ○ Mostly disagree
- ○ Neither agree nor disagree
- ○ Mostly agree
- ○ Totally agree

----Page Break----

T2 INT Q5-8

How much do you agree with the following statements?

T2 INT Q5

5. I would like my body to look like the people who are in movies.

- ○ Totally disagree
- ○ Mostly disagree
- ○ Neither agree nor disagree
- ○ Mostly agree
- ○ Totally agree

T2 INT Q6

6. I compare my body to the bodies of people who appear in magazines.

- ○ Totally disagree
- ○ Mostly disagree
- ○ Neither agree nor disagree
- ○ Mostly agree
- ○ Totally agree

T2 INT Q7
7. I wish I looked like the models in music videos.

- ◯ Totally disagree
- ◯ Mostly disagree
- ◯ Neither agree nor disagree
- ◯ Mostly agree
- ◯ Totally agree

T2 INT Q8
8. I compare my appearance to the appearance of people in magazines.

- ◯ Totally disagree
- ◯ Mostly disagree
- ◯ Neither agree nor disagree
- ◯ Mostly agree
- ◯ Totally agree

----Page Break----

T2 INT Q9-12
How much do you agree with the following statements?

T2 INT Q9
9. I try to look like the people on TV.

- ◯ Totally disagree
- ◯ Mostly disagree
- ◯ Neither agree nor disagree
- ◯ Mostly agree
- ◯ Totally agree

T2 INT Q10

10. I compare my body to the bodies of people who are on social media.

- ○ Totally disagree
- ○ Mostly disagree
- ○ Neither agree nor disagree
- ○ Mostly agree
- ○ Totally agree

T2 INT Q11

11. I would like my body to look like the bodies of people who are on social media.

- ○ Totally disagree
- ○ Mostly disagree
- ○ Neither agree nor disagree
- ○ Mostly agree
- ○ Totally agree

T2 INT Q12

12. I try to look like the people I see on social media.

- ○ Totally disagree
- ○ Mostly disagree
- ○ Neither agree nor disagree
- ○ Mostly agree
- ○ Totally agree

End of Block: Internalisation / Page Break

Start of Block: Progress note

T2 Progress note

Congratulations! You have completed more than half the survey!

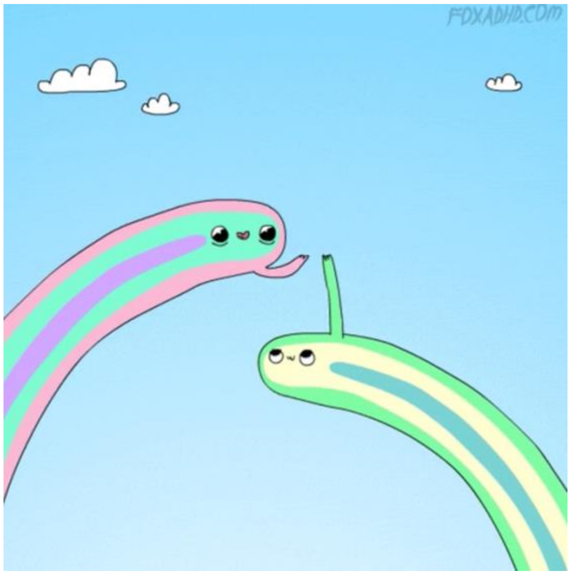

via GIPHY

End of Block: Progress note / Page Break

Start of Block: PANAS

T2 PANAS Q1-6
For each item below, please select the response that best describes how much you have felt that way **over the past week**.

	Never	Rarely	Sometimes	Often	Always
1. **Interested**	○	○	○	○	○
2. **Alert**	○	○	○	○	○
3. **Excited**	○	○	○	○	○
4. **Happy**	○	○	○	○	○
5. **Strong**	○	○	○	○	○
6. **Energetic**	○	○	○	○	○

----Page Break----

T2 PANAS Q7-12
For each item below, please select the response that best describes how much you have felt that way over the past week.

	Never	Rarely	Sometimes	Often	Always
7. **Calm**	◯	◯	◯	◯	◯
8. **Cheerful**	◯	◯	◯	◯	◯
9. **Active**	◯	◯	◯	◯	◯
10. **Proud**	◯	◯	◯	◯	◯
11. **Joyful**	◯	◯	◯	◯	◯
12. **Fearless**	◯	◯	◯	◯	◯

----Page Break----

T2 PANAS Q13-18
For each item below, please select the response that best describes how much you have felt that way over the past week.

	Never	Rarely	Sometimes	Often	Always
13. **Delighted**	◯	◯	◯	◯	◯
14. **Daring**	◯	◯	◯	◯	◯
15. **Sad**	◯	◯	◯	◯	◯
16. **Frightened**	◯	◯	◯	◯	◯
17. **Ashamed**	◯	◯	◯	◯	◯
18. **Upset**	◯	◯	◯	◯	◯

----Page Break----

T2 PANAS Q19-23 +AC2
For each item below, please select the response that best describes how much you have felt that way over the past week.

	Never	Rarely	Sometimes	Often	Always
19. **Nervous**	○	○	○	○	○
20. **Guilty**	○	○	○	○	○
21. **Please choose "never".**	○	○	○	○	○
22. **Scared**	○	○	○	○	○
23. **Miserable**	○	○	○	○	○
24. **Jittery/jumpy**	○	○	○	○	○

----Page Break----

T2 PANAS Q24-28

For each item below, please select the response that best describes how much you have felt that way over the past week.

	Never	Rarely	Sometimes	Often	Always
25. **Afraid**	○	○	○	○	○
26. **Lonely**	○	○	○	○	○
27. **Mad**	○	○	○	○	○
28. **Disgusted**	○	○	○	○	○
29. **Gloomy**	○	○	○	○	○

End of Block: PANAS / Page Break

Start of Block: Skin Shade satisfaction

T2 Skin Q1

How dissatisfied or satisfied you are with the **colour of your skin?**

- ◯ Very dissatisfied
- ◯ Mostly dissatisfied
- ◯ Neither satisfied nor dissatisfied
- ◯ Mostly satisfied
- ◯ Very satisfied

T2 Skin Q2

Which of the following statements do you **agree** with the most:

- ◯ I would like my skin colour to be lighter
- ◯ I would like my skin colour to stay the same
- ◯ I would like my skin colour to be darker

----Page Break----

T2Skin chart_current

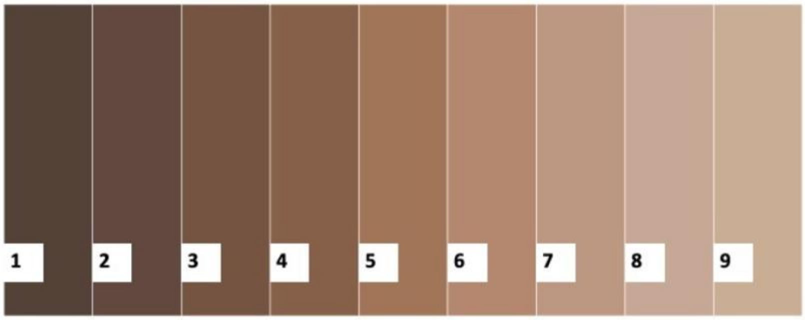

Using the chart above, please select the number that best represents **your current skin shade**.

- 1
- 2
- 3
- 4
- 5
- 6
- 7
- 8
- 9

----Page Break----

T2 Skin chart_ideal

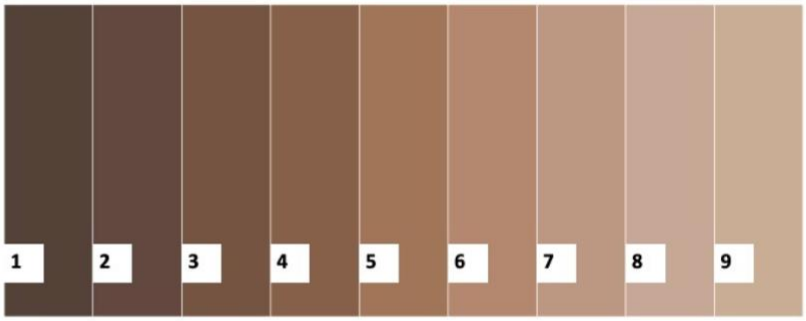

Using the chart above, select the number that best represents **the skin shade that you would like for yourself - your 'ideal' skin shade**.

- 1
- 2
- 3
- 4
- 5
- 6
- 7
- 8
- 9

End of Block: Skin Shade satisfaction / Page Break

Start of Block: Intervention or Control?

Sent video links?
Were you sent links to watch the Warna-Warni Waktu video series over the last week?

Please take care to answer this question correctly as you will not be able to return to this screen after hitting the “Next” button.

- Yes
- No

Then Branch If:
If Were you sent links to watch the Warna-Warni Waktu video series over the last week? …= ***No***
Then Show Block: Support

End of Block: Intervention or Control?

Start of Block: Video series feedback

Display This Question:
If Were you sent links to watch the Warna Warni Waktu video series over the last week? Please take... = Yes

Video feedback intro

We'd now like to ask you some questions about the video series, Warna-Warni Waktu, that we've been sharing with you. Please be as honest as possible.

Please select the response that best reflects how much you agree with the following statements:

1. The Warna-Warni Waktu video series was **easy to understand.**

- ○ Strongly disagree
- ○ Disagree
- ○ Neither agree nor disagree
- ○ Agree
- ○ Strongly agree
- ○ I did not watch any of the video series.

2. I **liked the characters** in the Warna-Warni Waktu video series.

- ○ Strongly disagree
- ○ Disagree
- ○ Neither agree nor disagree
- ○ Agree
- ○ Strongly agree
- ○ I did not watch any of the video series.

3. I **enjoyed** watching the Warna-Warni Waktu video series.

- ○ Strongly disagree
- ○ Disagree
- ○ Neither agree nor disagree
- ○ Agree
- ○ Strongly agree
- ○ I did not watch any of the video series.

4. The Warna-Warni Waktu video series is **appropriate** for young women my age.

- Strongly disagree
- Disagree
- Neither agree nor disagree
- Agree
- Strongly agree
- I did not watch any of the video series.

5. The Warna Warni Waktu video series taught me how to be more **body confident**.

- Strongly disagree
- Disagree
- Neither agree nor disagree
- Agree
- Strongly agree
- I did not watch any of the video series.

6. I would **share** the Warna-Warni Waktu video series with young women my age.

- Strongly disagree
- Disagree
- Neither agree nor disagree
- Agree
- Strongly agree
- I did not watch any of the video series.

----Page Break----

Series feedback

We'd love to know a bit more about what you thought about the Warna-Warni Waktu **video series**.

Please use the space below to write down anything you particularly liked or didn't like about the Warna-Warni Waktu **video series**.

____
____
____
____

UWE Bristol University of the West of England

Activity feedback
We'd also love to know what you thought about the Warna-Warni Waktu **activities** you were given.

Please use the space below to write down anything you particularly liked or didn't like about the Warna-Warni Waktu **activities.**

____
____
____
____
____

End of Block: Video series feedback

Start of Block: Internet connection

Display This Question:
If Were you sent links to watch the Warna Warni Waktu video series over the last week? Please take... = Yes

T2 Int connection
We would like to know what was your internet connection like whilst viewing the video series over the past week?

- ○ *I had a good connection and was able to view the videos easily*.
- ○ *My connection was okay but I sometimes struggled to view the videos.*
- ○ *I had poor connection and struggled to watch the videos.*

Display This Question:
If We would like to know what was your internet connection like whilst viewing the video series over... = <i>My connection was okay but I sometimes struggled to view the videos.</i>
Or We would like to know what was your internet connection like whilst viewing the video series over... = I had poor connection and struggled to watch the videos.

T2 Internet issues
Please describe the internet problems you experienced while watching the video series.

____
____
____
____
____

End of Block: Internet connection

Start of Block: Viewed videos?

Display This Question:
If Were you sent links to watch the Warna-Warni Waktu video series over the last week? Please take... = Yes

Viewed videos?
Lastly, did you manage to watch all of the videos we sent to you over the last 6 days?

- ◯ Yes
- ◯ No
- ◯ I'm not sure

Skip To: End of Block If Lastly, did you manage to watch all of the videos we sent to you over the last 6 days? = Yes

Display This Question:
If Lastly, did you manage to watch all of the videos we sent to you over the last 6 days? = No
Or Lastly, did you manage to watch all of the videos we sent to you over the last 6 days? = I'm not sure

Reason not viewed
If you can, please select what stopped you from watching the videos we sent to you.

- ◯ I did not have access to a phone or a device
- ◯ I didn't have any phone credit
- ◯ I found the videos boring.
- ◯ I did not receive a link to watch them.
- ◯ I didn't have an internet connection.
- ◯ I forgot.
- ◯ I didn't realise I had to watch the videos.
- ◯ I didn't have the time.
- ◯ Other. Please explain.

____

End of Block: Viewed videos?

Start of Block: Support

T2 Support

Have you sought extra support related to body image, since you completed the last survey? (e.g., school counsellor, teacher, online support, family member, friend)

○ No

○ Yes. Please describe what this support involved, in as much detail as you can (if you feel comfortable). ____

End of Block: Support

Start of Block: Final message

Final

Thank you for your participation! We'll be sharing one more similar survey with you in four weeks' time. 📅

It's important you complete it as soon as possible once you receive it, so look out for it! 👀

Many thanks again for helping us with this important work! You are helping to improve the lives of adolescent girls across Indonesia. 🙌🏿

Please click "Next" to close the survey. ➡️

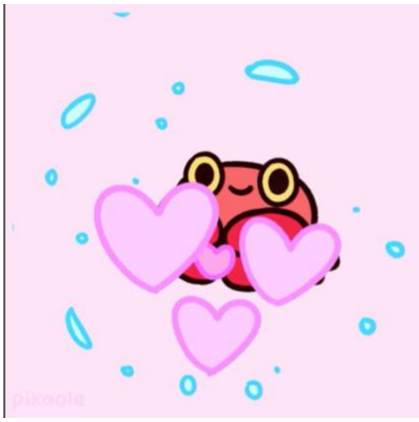

via GIPHY

End of Block: Final message

Thank you for taking the time to complete this survey.
Your response has been recorded.

Time Three (T3) Questionnaire

Start of Block: Participant number

Participant number
Please enter the Participant Number that the researcher gave you.

____

End of Block: Participant number / Page Break

Start of Block: Consent

Consent 2
Do you agree to complete one more survey again for us today? This is the last time we will ask you to complete a survey - we promise!

- ○ Yes, let's do it!
- ○ No, thank you for asking.

Display This Question:
If Do you agree to complete one more survey again for us today? This is the last time we will ask yo... = No, thank you for asking.

Consent check
Are you sure?

- ○ I am sure. I do not want to take part in this study.
- ○ Whoops! I hit the wrong button. I would like to take part in this study today.

Then Branch If:
If Are you sure? = I am sure. I do not want to take part in this study.
Then: End of Survey

Display This Question:
If Do you agree to complete one more survey again for us today? This is the last time we will ask yo... = Yes, let's do it!
Or Are you sure? = Whoops! I hit the wrong button. I would like to take part in this study today.

Consent reminder
Before you get started, we would like to remind you about some important information about this survey:

1. This is **not a test.** Everyone will have different answers and there are no right or wrong answers.

2. Although it is not a test, it is really important that you **complete your survey on your own, in a quiet space, if you can.** If you have any questions, please ask the researcher who will be happy to help you.

UWE Bristol University of the West of England

3. All your answers are **completely confidential** – your parents, family and friends won't see them.

4. If you do not feel comfortable answering a particular question, **just skip it** and go on to the next question.

5. **If you do not want to do the survey**, that's OK. Just let the researcher know. You can stop completing the survey at any point, too. Again, just let the researcher know.

6. If you understand everything we've just told you, **please press "next" to begin**.

End of Block: Consent / Page Break

Start of Block: UPC

UPC
Thank you for agreeing to complete this final survey! Next, we need you to recreate your unique code that you created a few weeks ago. This will enable us to link what you tell us today to what you told us in the previous surveys. As with the first survey, please answer the following two questions:

UPC - First name
1. Write the **first two letters** of your **first name.**
(Example: if your name is ARIF, you would enter AR).

____

UPC - Month of birth
2. Select the **month** you were born in.

- ○ January
- ○ February
- ○ March
- ○ April
- ○ May
- ○ June
- ○ July
- ○ August

- ◯ September
- ◯ October
- ◯ November
- ◯ December

End of Block: UPC

Start of Block: Instructions

Instruction refresh?
It's been a few weeks since you completed a survey for us. Would you like a refresher on how to complete the questions?

- ◯ Yes, please!
- ◯ No, I remember what I need to do.

Skip To: Start If It's been a few weeks since you completed a survey for us. Would you like a refresher on how to c... = No, I remember what I need to do.

Display This Question:
If It's been a few weeks since you completed a survey for us. Would you like a refresher on how to c... = Yes, please!

Example intro
As before, this survey asks questions about some of your everyday experiences and feelings about yourself and your body.

You will need to select the answer that matches how you think and feel about important areas of your life.

Remember, there are no right or wrong answers.

Let's try an example.

Click "Next" to start the example question. →

----Page Break----

1st example question
How often do you engage in the following activity?

I listen to music.

Over to you. Select the response that best describes how often you listen to music.

- Never
- Rarely
- Sometimes
- Often
- Always

----Page Break----

T3 Check 1

Not too difficult, right?

- If you listen to music every day, you would have chosen “always”.
- If you listen to music almost every day, you would have chosen “often”.
- If you listen to music two or three times a week, you would have chosen “sometimes”.
- If you listen to music once in a while, perhaps once a week or less, you would have chosen “rarely”.
- Lastly, if you never listen to music, you would have chosen “never”.

Do you feel ready to get started?

- Yes, let me get started.
- No, I'd like another practice question, please.

Skip To: Start If Not too difficult, right? If you listen to music every day, you would have chosen “always”. ... = Yes, let me get started.

Display This Question:

If Not too difficult, right? If you listen to music every day, you would have chosen “always”. ... = No, I'd like another practice question, please.

T3 2nd example

Let’s try another example question.

How often do you engage in the following activity?

I send text messages to my friends.

Now, select the response that best describes how often you send text messages to your friends.

○ Never

○ Rarely

○ Sometimes

○ Often

○ Always

----Page Break----

T3 Check 2

- If you send text messages to your friends every day, you would have chosen “always”.
- If you send text messages to your friends almost every day, you would have chosen “often”.
- If you send text messages to your friends two or three times a week, you would have chosen “sometimes”.
- If you send text messages to your friends once in a while, perhaps once a week or less, you would have chosen “rarely”.
- Lastly, if you never send text messages to your friends, you would have chosen “never”.

Now, do you feel ready to get started?

○ Yes, let me get started.

○ No, I'm still not sure.

Skip To: Start If If you send text messages to your friends every day, you would have chosen “always”. If you send... = Yes, let me get started.

Display This Question:
If If you send text messages to your friends every day, you would have chosen “always”. If you send... = No, I'm still not sure.

Extra help required

Please WhatsApp the researcher for further explanation on how to complete the questions. After speaking with the researcher and are clear on how to complete the questions, please click "Next" to begin. →

Display This Question:
If If you send text messages to your friends every day, you would have chosen "always". If you send... = Yes, let me get started.
Or Not too difficult, right? If you listen to music every day, you would have chosen "always". ... = Yes, let me get started.
Or Or Please WhatsApp the researcher for further explanation on how to complete the questions. After... Is Displayed
Or It's been a few weeks since you completed a survey for us. Would you like a refresher on how to c... = No, I remember what I need to do.

End of Block: Instructions / Page Break

Start of Block: UPDATED BESAA

T3 BES Q1,2,4,5
Please select how often each of the following statements is true of you:

T3 BES Q1
1. I like what I look like in pictures (*photos*).

- ◯ Never
- ◯ Rarely
- ◯ Sometimes
- ◯ Often
- ◯ Always

T3 BES Q2
2. Other people consider me good looking (*beautiful/handsome/pretty*).

- ◯ Never
- ◯ Rarely
- ◯ Sometimes
- ◯ Often
- ◯ Always

T3 BES Q4
3. I am preoccupied with trying to change my body weight.

- Never
- Rarely
- Sometimes
- Often
- Always

T3 BES Q5
4. I think my appearance would help me get a job.

- Never
- Rarely
- Sometimes
- Often
- Always

----Page Break----

T3 BES Q6,8,10,11
Please select how often each of the following statements is true of you:

T3 BES Q6
5. I like what I see when I look in the mirror.

- Never
- Rarely
- Sometimes
- Often
- Always

T3 BES Q8

6. I am satisfied with my weight.

- ◯ Never
- ◯ Rarely
- ◯ Sometimes
- ◯ Often
- ◯ Always

T3 BES Q10

7. I really like what I weigh.

- ◯ Never
- ◯ Rarely
- ◯ Sometimes
- ◯ Often
- ◯ Always

T3 BES Q11

8. I wish I looked like someone else.

- ◯ Never
- ◯ Rarely
- ◯ Sometimes
- ◯ Often
- ◯ Always

----Page Break----

T3BESQ12,13,14,15,16

Please select how often each of the following statements is true of you:

T3 BES Q12

9. People my own age like my looks.

- ○ Never
- ○ Rarely
- ○ Sometimes
- ○ Often
- ○ Always

T3 BES Q13

10. My looks upset me (sad).

- ○ Never
- ○ Rarely
- ○ Sometimes
- ○ Often
- ○ Always

T3 BES Q14

11. I'm as nice looking as most people.

- ○ Never
- ○ Rarely
- ○ Sometimes
- ○ Often
- ○ Always

T3 BES Q15

12. I'm pretty happy about the way I look.

- ○ Never
- ○ Rarely
- ○ Sometimes
- ○ Often
- ○ Always

T3 BES Q16

13. I feel I weigh the right amount for my height.

- Never
- Rarely
- Sometimes
- Often
- Always

----Page Break----

T3BESQ17,AC,19,20,21

Please select how often each of the following statements is true of you:

T3 BES Q17

14. I feel ashamed of how I look.

- Never
- Rarely
- Sometimes
- Often
- Always

T3 BES AC1

15. Please choose "always"

- Never
- Rarely
- Sometimes
- Often
- Always

T3 BES Q19

16. My weight makes me unhappy.

- ○ Never
- ○ Rarely
- ○ Sometimes
- ○ Often
- ○ Always

T3 BES Q20

17. My looks make me attractive (beautiful/handsome/pretty) and popular.

- ○ Never
- ○ Rarely
- ○ Sometimes
- ○ Often
- ○ Always

T3 BES Q21

18. I worry about the way I look.

- ○ Never
- ○ Rarely
- ○ Sometimes
- ○ Often
- ○ Always

End of Block: UPDATED BESAA / Page Break

Start of Block: Internalisation

T3 INT Q1-4

How much do you agree with the following statements?

T3 INT Q1

1. I would like my body to look like the bodies of people who are on TV.

- ○ Totally disagree
- ○ Mostly disagree
- ○ Neither agree nor disagree
- ○ Mostly agree
- ○ Totally agree

T3 INT Q2

2. I compare my body to the bodies of people who are on TV.

- ○ Totally disagree
- ○ Mostly disagree
- ○ Neither agree nor disagree
- ○ Mostly agree
- ○ Totally agree

T3 INT Q3

3. I would like my body to look like the models who appear in magazines.

- ○ Totally disagree
- ○ Mostly disagree
- ○ Neither agree nor disagree
- ○ Mostly agree
- ○ Totally agree

T3 INT Q4

4. I compare my appearance to the appearance of TV and movie stars.

- ◯ Totally disagree
- ◯ Mostly disagree
- ◯ Neither agree nor disagree
- ◯ Mostly agree
- ◯ Totally agree

----Page Break----

T3 INT Q5-8
How much do you agree with the following statements?

T3 INT Q5
5. I would like my body to look like the people who are in movies.

- ◯ Totally disagree
- ◯ Mostly disagree
- ◯ Neither agree nor disagree
- ◯ Mostly agree
- ◯ Totally agree

T3 INT Q6
6. I compare my body to the bodies of people who appear in magazines.

- ◯ Totally disagree
- ◯ Mostly disagree
- ◯ Neither agree nor disagree
- ◯ Mostly agree
- ◯ Totally agree

T3 INT Q7
7. I wish I looked like the models in music videos.

- ○ Totally disagree
- ○ Mostly disagree
- ○ Neither agree nor disagree
- ○ Mostly agree
- ○ Totally agree

T3 INT Q8
8. I compare my appearance to the appearance of people in magazines.

- ○ Totally disagree
- ○ Mostly disagree
- ○ Neither agree nor disagree
- ○ Mostly agree
- ○ Totally agree

----Page Break----

T3 INT Q9-12
How much do you agree with the following statements?

T3 INT Q9
9. I try to look like the people on TV.

- ○ Totally disagree
- ○ Mostly disagree
- ○ Neither agree nor disagree
- ○ Mostly agree
- ○ Totally agree

T3 INT Q10

10. I compare my body to the bodies of people who are on social media.

- ○ Totally disagree
- ○ Mostly disagree
- ○ Neither agree nor disagree
- ○ Mostly agree
- ○ Totally agree

T3 INT Q11

11. I would like my body to look like the bodies of people who are on social media.

- ○ Totally disagree
- ○ Mostly disagree
- ○ Neither agree nor disagree
- ○ Mostly agree
- ○ Totally agree

T3 INT Q12

12. I try to look like the people I see on social media.

- ○ Totally disagree
- ○ Mostly disagree
- ○ Neither agree nor disagree
- ○ Mostly agree
- ○ Totally agree

End of Block: Internalisation / Page Break

Start of Block: Progress note

T3 Progress note

Congratulations! You have completed more than half the survey!

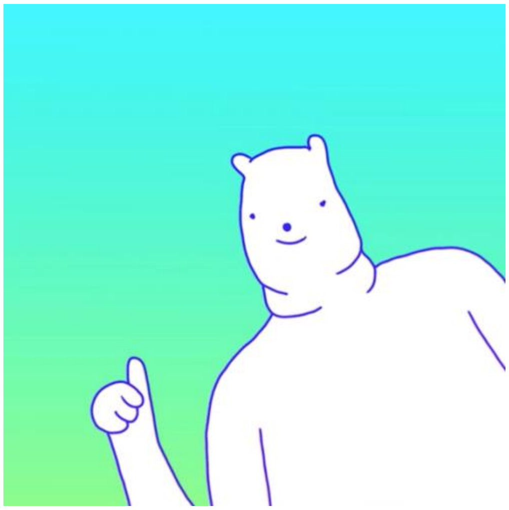

via GIPHY

End of Block: Progress note / Page Break

Start of Block: PANAS

T3 PANAS Q1-6
For each item below, please select the response that best describes how much you have felt that way **over the past week**.

	Never	Rarely	Sometimes	Often	Always
1. **Interested**	○	○	○	○	○
2. **Alert**	○	○	○	○	○
3. **Excited**	○	○	○	○	○
4. **Happy**	○	○	○	○	○
5. **Strong**	○	○	○	○	○
6. **Energetic**	○	○	○	○	○

----Page Break----

T3 PANAS Q7-12

For each item below, please select the response that best describes how much you have felt that way over the past week.

	Never	Rarely	Sometimes	Often	Always
7. **Calm**	○	○	○	○	○
8. **Cheerful**	○	○	○	○	○
9. **Active**	○	○	○	○	○
10. **Proud**	○	○	○	○	○
11. **Joyful**	○	○	○	○	○
12. **Fearless**	○	○	○	○	○

----Page Break----

T3 PANAS Q13-18

For each item below, please select the response that best describes how much you have felt that way over the past week.

	Never	Rarely	Sometimes	Often	Always
13. **Delighted**	○	○	○	○	○
14. **Daring**	○	○	○	○	○
15. **Sad**	○	○	○	○	○
16. **Frightened**	○	○	○	○	○
17. **Ashamed**	○	○	○	○	○
18. **Upset**	○	○	○	○	○

----Page Break----

T3 PANAS Q19-23 +AC2

For each item below, please select the response that best describes how much you have felt that way over the past week.

	Never	Rarely	Sometimes	Often	Always
19. **Nervous**	◯	◯	◯	◯	◯
20. **Guilty**	◯	◯	◯	◯	◯
21. **Please choose "sometimes".**	◯	◯	◯	◯	◯
22. **Scared**	◯	◯	◯	◯	◯
23. **Miserable**	◯	◯	◯	◯	◯
24. **Jittery/jumpy**	◯	◯	◯	◯	◯

----Page Break----

T3 PANAS Q24-28
For each item below, please select the response that best describes how much you have felt that way over the past week.

	Never	Rarely	Sometimes	Often	Always
25. **Afraid**	◯	◯	◯	◯	◯
26. **Lonely**	◯	◯	◯	◯	◯
27. **Mad**	◯	◯	◯	◯	◯
28. **Disgusted**	◯	◯	◯	◯	◯
29. **Gloomy**	◯	◯	◯	◯	◯

End of Block: PANAS / Page Break

Start of Block: Skin Shade satisfaction

T3 Skin Q1

How dissatisfied or satisfied you are with the **colour of your skin?**

- ○ Very dissatisfied
- ○ Mostly dissatisfied
- ○ Neither satisfied nor dissatisfied
- ○ Mostly satisfied
- ○ Very satisfied

T3 Skin Q2

Which of the following statements do you **agree** with the most:

- ○ I would like my skin colour to be lighter
- ○ I would like my skin colour to stay the same
- ○ I would like my skin colour to be darker

----Page Break----

T3Skin chart_current

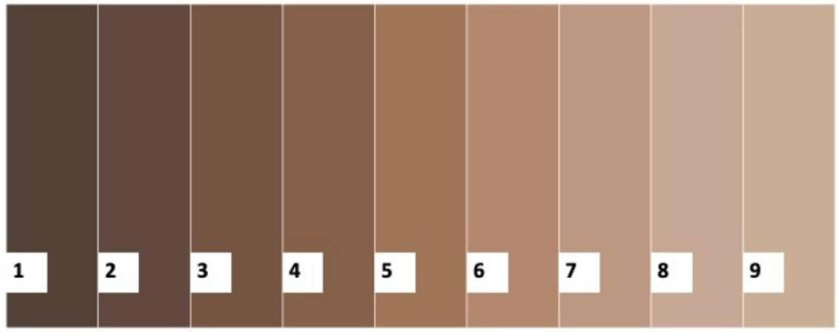

Using the chart above, please select the number that best represents **your current skin shade**.

- ○ 1
- ○ 2
- ○ 3
- ○ 4
- ○ 5
- ○ 6
- ○ 7
- ○ 8
- ○ 9

----Page Break----

T3 Skin chart_ideal

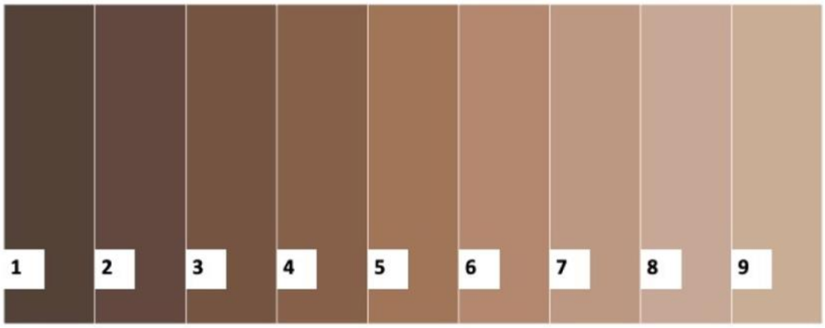

Using the chart above, select the number that best represents **the skin shade that you would like for yourself - your 'ideal' skin shade**.

○ 1

○ 2

○ 3

○ 4

○ 5

○ 6

○ 7

○ 8

○ 9

End of Block: Skin Shade satisfaction / Page Break

Start of Block: T3 Support

Support
Have you sought extra support related to body image, since you completed the last survey? (e.g., school counsellor, teacher, online support, family member, friend)

○ No

○ Yes. Please describe what this support involved, in as much detail as you can (if you feel comfortable). ____

End of Block: T3 Support

Start of Block: Final msg

T3 Final
Many thanks again for helping us with this important work! 🙏🏽

Your participation has truly been invaluable to us!

As a small thank you, please contact the researcher to receive your Certificate of Completion. ✨
Please click "next" to close the survey. ➡️

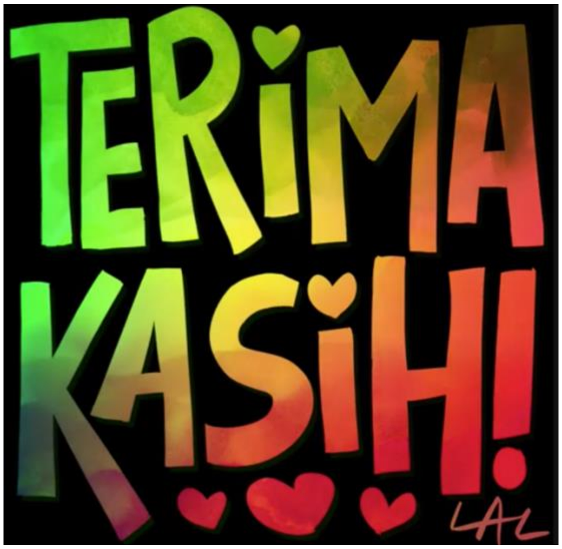

via GIPHY

End of Block: Final msg

Thank you for taking the time to complete this survey.
Your response has been recorded.

Intervention and state measures questionnaire 1

Start of Block: Participant Number

Participant number
Please enter the Participant Number that the researcher gave you.

____

End of Block: Participant Number / Page Break

Start of Block: Consent

Consent
Welcome back – we're so happy you're here! 👋🏾

Today we are sharing the first of a series of six videos for you to watch. The video is less than 5 minutes long. It's important that you try and watch this video in a quiet area free from distractions.

As well as showing you a video, we will also ask you to answer a few very short questions about how you're feeling today. At the end of the survey, you will be invited to take part in an activity that will be related to the video you will have just watched.

Sound good?

○ Yes, let's go!

○ No, thanks!

Skip To: End of Block If Welcome back – we're so happy you're here! 👋 Today we are sharing the first of a series of six... = Yes, let's go!

Display This Question:
If Welcome back – we're so happy you're here! 👋 Today we are sharing the first of a series of six... = No, thanks!

Consent check
Are you sure? We really value your thoughts and opinions and would like very much for you to participate.

○ I am sure. I do not want to take part today.

○ I would like to take part in this study today.

Then Branch If:
If Are you sure? We really .... = I am sure. I do not want to take part today.
Then: End of Survey

End of Block: Consent / Page Break

Start of Block: UPC

UPC
Thank you! 🙏🏿
Before we start, please complete the two questions below that together make up your **unique participant code**.

UPC first name
1. Write the first two letters of your first name.
(Example: if your name is ARIF, you would enter AR).

____

UPC month
2. Select the **MONTH** you were born in.

- ◯ January
- ◯ February
- ◯ March
- ◯ April
- ◯ May
- ◯ June
- ◯ July
- ◯ August
- ◯ September
- ◯ October
- ◯ November
- ◯ December

End of Block: UPC / Page Break

Start of Block: Instructions

Instructions

Our first task for you is to answer two quick questions about how you feel RIGHT NOW, IN THIS MOMENT.

Before we begin, let's try an example question.

----Page Break----

1st example question

The example question is**:**

How tired do you feel RIGHT NOW?

Your answer depends on how you are feeling. For instance,

- If you are not feeling tired at all, you might keep the slider at ‘0’.
- If you are feeling a little tired, you might move the slider to ’25’.
- If you are feeling somewhat tired, you might move the slider to the number ‘50’.
- If you are feeling tired but not extremely tired, you might move to the slider to ‘75’.
- If you are feeling extremely tired, you might move the slider to ‘100’.

Okay, now you give it a try. Use the slider below and share **how tired you feel RIGHT NOW**.

0 = Not at all — Extremely = 100

0 10 20 30 40 50 60 70 80 90 100

----Page Break----

1st eg Q check

Do you understand how to answer this type of question?

○ Yes, I'm ready to start!

○ No, not quite.

Skip To: End of Block If Do you understand how to answer this type of question? = Yes, I'm ready to start!

Display This Question:

If Do you understand how to answer this type of question? = No, not quite.

2nd example question

Let’s try another example question.

How hungry do you feel RIGHT NOW?

- If you are not hungry at all, you might move keep the slider at '0'.
- If are feeling a little bit hungry, you might move the slider to ‘25’
- If you are feeling somewhat hungry, you might move the slider to ’50’
- If you are feeling hungry but not extremely hungry you might move the slider to ’75’.
- If you are feeling extremely hungry, you might move the slider to ‘100’.

Okay, you give it a go. How hungry do you feel RIGHT NOW?

0 = Not at all Extremely = 100

0 10 20 30 40 50 60 70 80 90 100

---Page Break---

2nd ex Q check

Do you now understand how to complete this type of question?

◯ Yes, let me get started!

◯ No, I'm not sure.

Skip To: End of Block If Do you now understand how to complete this type of question? = Yes, let me get started!

Display This Question:

If Do you now understand how to complete this type of question? = No, I'm not sure.

Extra help required

Please WhatsApp the researcher for further explanation on how to complete the questions.

After speaking with the researcher and are clear on how to complete the questions, please click "Next" to begin. →

End of Block: Instructions / Page Break

Start of Block: Questions

Display This Question:

If Do you understand how to answer this type of question? = Yes, I'm ready to start!

Or Do you now understand how to complete this type of question? = Yes, let me get started!

Start Let's begin! 🚀 (And remember, the researcher will be available via WhatsApp in case you have any questions.)

----Page Break----

pre-video BI V1

How satisfied do you feel about your appearance (the way you look), **RIGHT NOW**?

0 = Not at all Extremely = 100

0 10 20 30 40 50 60 70 80 90 100

----Page Break----

pre-video mood V1

How happy do you feel, RIGHT NOW?

0 = Not at all Extremely = 100

0 10 20 30 40 50 60 70 80 90 100

----Page Break----

Episode 1 video

Now you are ready to watch episode one in the six-episode series, Warna-Warni Waktu.

You should know that we plan on sharing this video series on social media in the near future, so you might hear or see instructions to “like this video”, “share it with your friends”, or write your thoughts “in the comment section”.

Please don’t worry about any of these references (just ignore them 🙈).

All you need to do today, and for the next five days, is watch the video 👀 and complete the short exercises that follow ✍🏽

Please press the "Play" arrow to begin ▶️

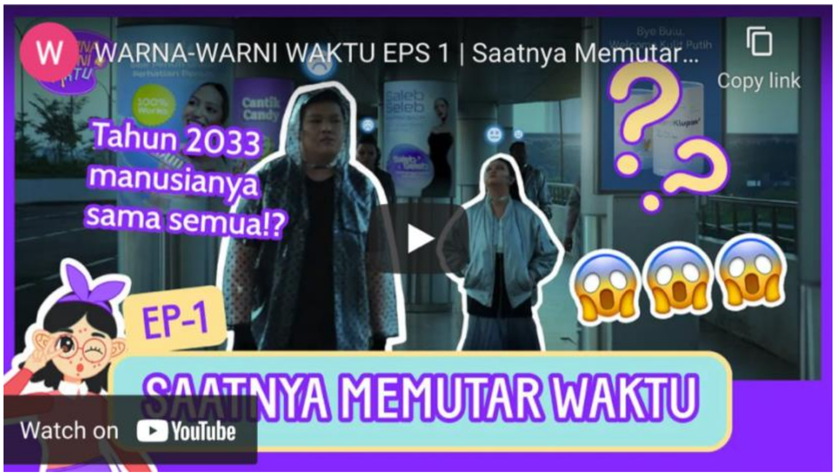

Timer 240 seconds (4:00 minutes)
Timing
First Click
Last Click
Page Submit
Click Count

----Page Break----

Post-video intro

Now, please answer two more quick questions about how you feel **RIGHT NOW**.

----Page Break----

Post-video BI V1

How satisfied do you feel about your appearance (the way you look), **RIGHT NOW**?

0 = Not at all Extremely = 100

0 10 20 30 40 50 60 70 80 90 100

----Page Break----

post-video mood V1

How happy do you feel, **RIGHT NOW**?

0 = Not at all Extremely = 100

0 10 20 30 40 50 60 70 80 90 100

End of Block: Questions / Page Break

Start of Block: Reinforcer

Reinforcer intro

Our last task for you today is a short activity that is related to the video you just watched.

----Page Break----

V1 Bonus activity a

Let's take a look at some of the key terms related to the video you just watched.

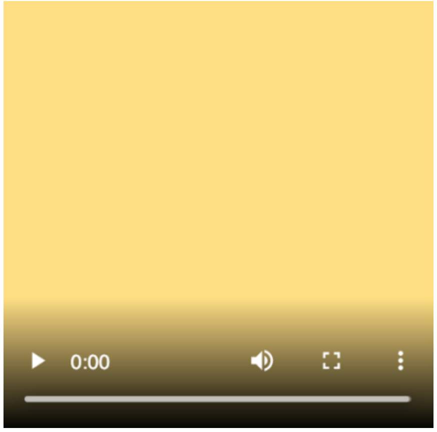

----Page Break----

V1 Bonus activity b

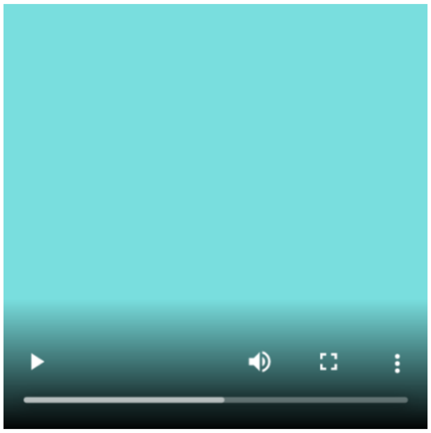

----Page Break----

V1 Bonus activity c

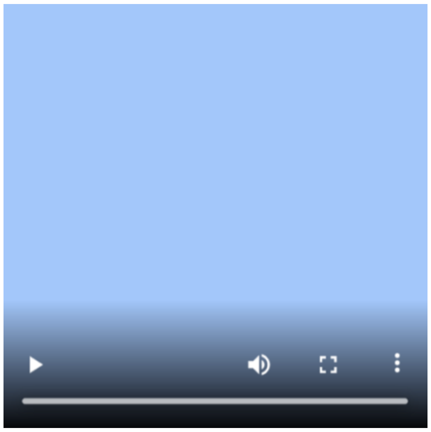

----Page Break----

V1 Bonus activity d

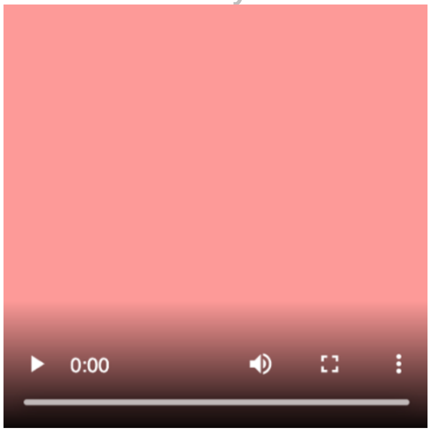

----Page Break----

V1 Bonus activity e

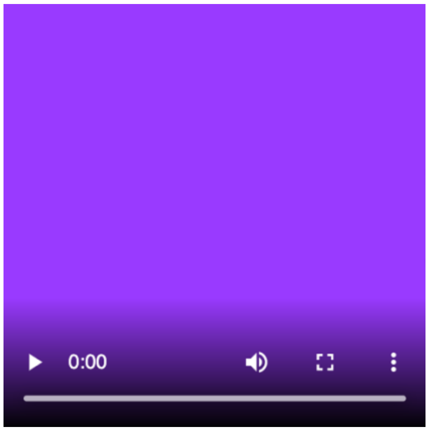

----Page Break----

V1 Bonus activity Q
Using some of these key terms, write one sentence about why Laras, Sekar, and their friends want Indonesian girls to feel confident about their bodies! ✍🏽

____
____
____
____
____

End of Block: Reinforcer / Page Break

Start of Block: Internet connection

Internet connection
You have completed your tasks for today – thank you!! 🙏🏽

Before you go, can you please let us know what your internet connection was like whilst viewing the video?

- ◯ I had a good connection and was able to view the video easily.
- ◯ My connection was okay but I sometimes struggled to view the video.
- ◯ I had poor connection and struggled to watch the video.

Display This Question:
If You have completed your tasks for today – thank you!! 🙏 Before you go, can you please let us kno... = My connection was okay but I sometimes struggled to view the video.
Or You have completed your tasks for today – thank you!! 🙏 Before you go, can you please let us kno... = I had poor connection and struggled to watch the video.

Internet problems

Please describe the problem(s) you had.

____

____

____

____

____

End of Block: Internet connection / Page Break

Start of Block: Final msg

Thank you Thank you for your participation! 🙌

We will share the link to the second video in the Warna-Warni Waktu series tomorrow. 📅

Many thanks again for helping us with this important work! 💫

Please click "Next" to close the survey. ➡️

End of Block: Final msg

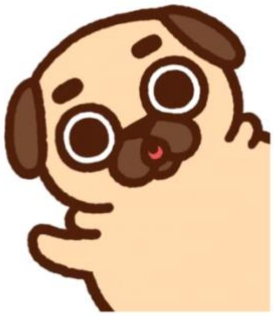

via GIPHY

Intervention and state measures questionnaire 2

Start of Block: Participant Number

Participant number
Please enter the Participant Number that the researcher gave you.

____

End of Block: Participant Number / Page Break

Start of Block: Consent

Consent
Great to have you back! 🤩

Today we are sharing the second episode of a series of six videos for you to watch.

Like yesterday’s video, today’s episode is less than 5 minutes long.

Please try and watch this video in a quiet area free from distractions.

Sound good/?

◯ Yes, let's go!

◯ No, thanks!

Skip To: End of Block If Great to have you back! 🤩 Today we are sharing the second episode of a series of six videos for... = Yes, let's go!

Display This Question:
If Great to have you back! 🤩 Today we are sharing the second episode of a series of six videos for... = No, thanks!

Consent check
Are you sure? We really value your thoughts and opinions and would like very much for you to participate.

◯ I am sure. I do not want to take part today.

◯ I would like to take part in this study today.

Then Branch If:
If Are you sure? We really .... = I am sure. I do not want to take part today.
Then: End of Survey

End of Block: Consent / Page Break

Start of Block: UPC

UPC

Thank you! 🙏

Before we start, please complete the two questions below that together make up your **unique participant code**.

UPC first name

1. Write the first two letters of your first name.
(Example: if your name is ARIF, you would enter AR).

____

UPC month

2. Select the **MONTH** you were born in.

- ◯ January
- ◯ February
- ◯ March
- ◯ April
- ◯ May
- ◯ June
- ◯ July
- ◯ August
- ◯ September
- ◯ October
- ◯ November
- ◯ December

End of Block: UPC / Page Break

Start of Block: Instructions

Instructions Our first task for you is to answer two quick questions about how you feel RIGHT NOW, IN THIS MOMENT. Would you like a refresher on how to complete the questions?

- ◯ Yes, please!
- ◯ No, I remember what to do.

Skip To: End of Block If Our first task for you is to answer two quick questions about how you feel RIGHT NOW, IN THIS MOM... = No, I remember what to do.

Display This Question:
If Our first task for you is to answer two quick questions about how you feel RIGHT NOW, IN THIS MOM... = Yes, please!

1st example question

The example question is

How excited do you feel RIGHT NOW?

Your answer depends on how you are feeling. For instance,

- If you are not feeling excited at all, you might keep the slider at ‘0’
- If you are feeling a little excited, you might move the slider to ’25
- If you are feeling somewhat excited, you might move the slider to the number ‘50’
- If you are feeling excited but not extremely excited, you might move to the slider to ‘75’
- If you are feeling extremely excited, you might move the slider to ‘100’.

Okay, now you give it a try.

Use the slider below and share **how excited you feel RIGHT NOW**.

0 = Not at all Extremely = 100

0 10 20 30 40 50 60 70 80 90 100

----Page Break----

1st eg Q check

Now are you ready to get started?

- ◯ Yes, I'm ready to start!
- ◯ No, I would like to practice with another question.

Skip To: End of Block If Now are you ready to get started? = Yes, I'm ready to start!

Display This Question:
If Now are you ready to get started? = No, I would like to practice with another question.

2nd example question

Let’s try another example question.

How thirsty do you feel RIGHT NOW?

- If you are not thirsty at all, you might move keep the slider at '0'
- If are feeling a little bit thirsty, you might move the slider to ‘25
- If you are feeling somewhat thirsty, you might move the slider to ’50’.
- If you are feeling thirsty but not extremely thirsty you might move the slider to ’75’.
- If you are feeling extremely thirsty, you might move the slider to ‘100’.

Okay, you give it a try.

How thirsty do you feel RIGHT NOW?

0 = Not at all Extremely = 100

0 10 20 30 40 50 60 70 80 90 100

----Page Break----

2nd ex Q check

Are you ready to get started?

○ Yes, let me get started!

○ No, I'm not sure.

Skip To: End of Block If Are you ready to get started? = Yes, let me get started!

Display This Question:
If Are you ready to get started? = No, I'm not sure.

Extra help required

Please WhatsApp the researcher for further explanation on how to complete the questions.

After speaking with the researcher and are clear on how to complete the questions, please click "Next" to begin. →

End of Block: Instructions / Page Break

Start of Block: Questions

Display This Question:
If Now are you ready to get started? = Yes, I'm ready to start!
Or Are you ready to get started? = Yes, let me get started!
Or Our first task for you is to answer two quick questions about how you feel RIGHT NOW, IN THIS MOM... = No, I remember what to do.

Start

Let's begin! 🚀 (And remember, the researcher will be available via WhatsApp in case you have any questions.)

----Page Break----

pre-video BI

How satisfied do you feel about your appearance (the way you look), **RIGHT NOW**?

0 = Not at all Extremely = 100

0 10 20 30 40 50 60 70 80 90 100

----Page Break----

pre-video mood How happy do you feel, RIGHT NOW?

0 = Not at all Extremely = 100

0 10 20 30 40 50 60 70 80 90 100

----Page Break----

Episode 2 video

Now you are ready to watch episode two in the six-episode series, Warna-Warni Waktu.

Just a reminder that you might hear or see instructions that reference social media during this video. Remember that you don't need to take any action except for watching the video 👀 and completing the exercises after the video ✍🏽

Please press the "Play" arrow to begin ▶️

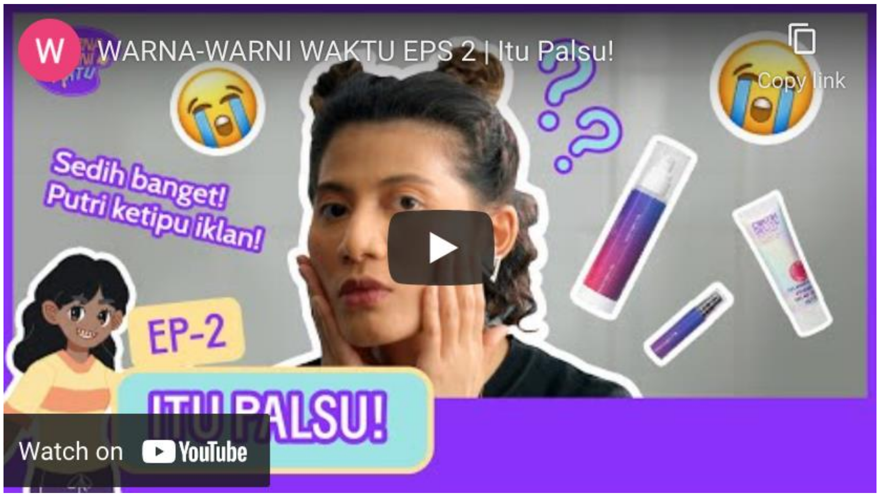

Timer 270 seconds (4:30 minutes)
Timing
First Click
Last Click
Page Submit
Click Count

----Page Break----

Post-video intro

Now, please answer two more quick questions about how you feel **RIGHT NOW**.

----Page Break----

Post-video BI V2

How satisfied do you feel about your appearance (the way you look), **RIGHT NOW**?

0 = Not at all Extremely = 100

0 10 20 30 40 50 60 70 80 90 100

----Page Break----

post-video mood V2
How happy do you feel, **RIGHT NOW**?

0 = Not at all Extremely = 100

0 10 20 30 40 50 60 70 80 90 100

End of Block: Questions / Page Break

Start of Block: Reinforcer

Reinforcer intro
We now have 5 short activities related to the video you just watched.

----Page Break----

V2 #1 CTA repeat
Here is the first activity:

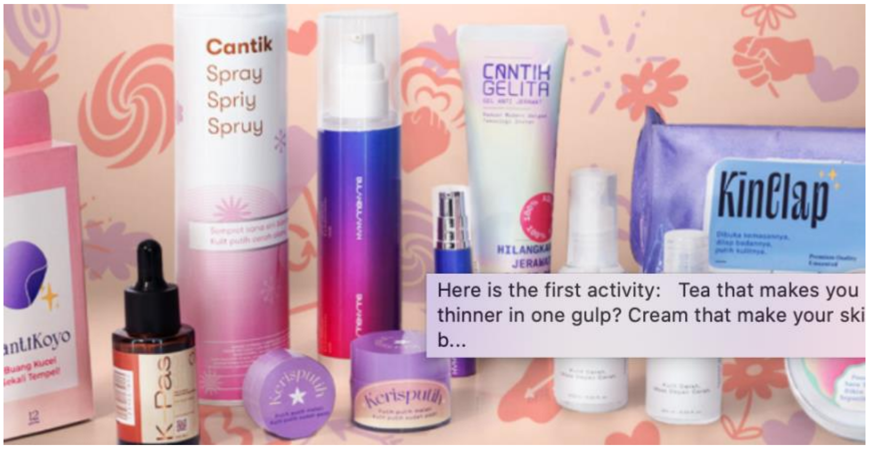

Tea that makes you thinner in one gulp? Cream that makes your skin brighter in a day? A pill to boost your height in a week? 🤔 When we think about it, there are countless ads on the internet that are illogical and unsafe 🤷‍♀️. Unfollowing them is one of the ways we can keep ourselves happy 😌 ✨ Have you ever encountered these kinds of ads online? Share your experience here! 🤳

____

____

____

____

____

----Page Break----

V2 #2BonusActivity1

Here is the second activity:

My oh my 😱 This is the digitally-edited video that Putri saw. There is stuff like this all around us that have huge differences between what is shown online and what reality is 😤.

Imagine that you are going to share this video on social media. What would your message say? Explain why you think it’s important that your friends and followers should watch this video 🏃‍♀️💖

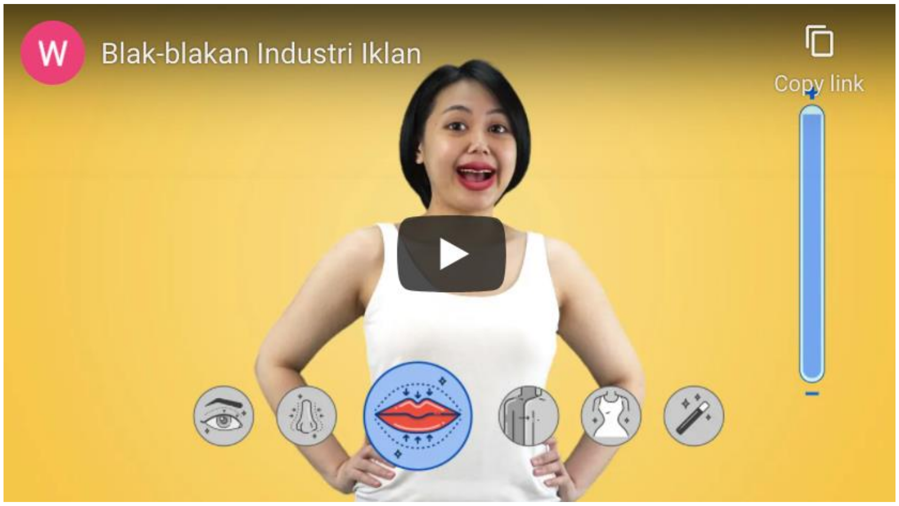

____

____

____

____

____

----Page Break----

V2 #3BonusActivity2

Here is the third activity:

Not everything you see on social media is true! 🙄 Here's an ad for a skin brightening lotion that popped up on Putri's phone. 📱 But look, the first ad is using an edited photo for promotional purposes and the second one is the ad with the unedited picture! 😱

Can you spot the differences between the two? Make a list of all the differences you can find and list them in the boxes below. 🔎

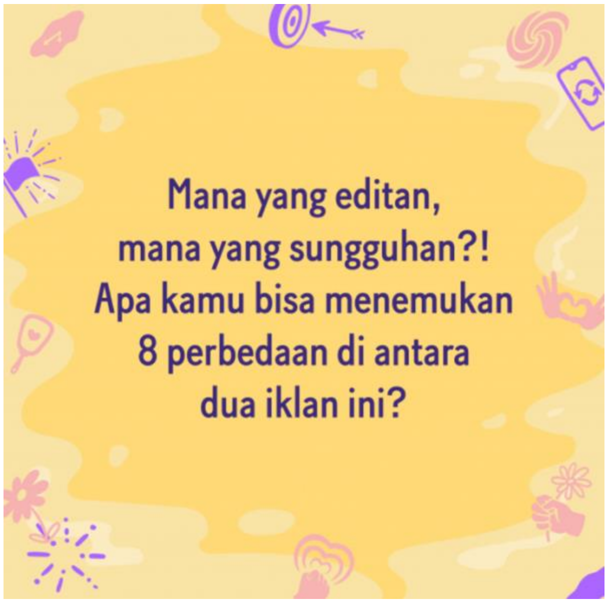
Mana yang editan,
mana yang sungguhan?!
Apa kamu bisa menemukan
8 perbedaan di antara
dua iklan ini?

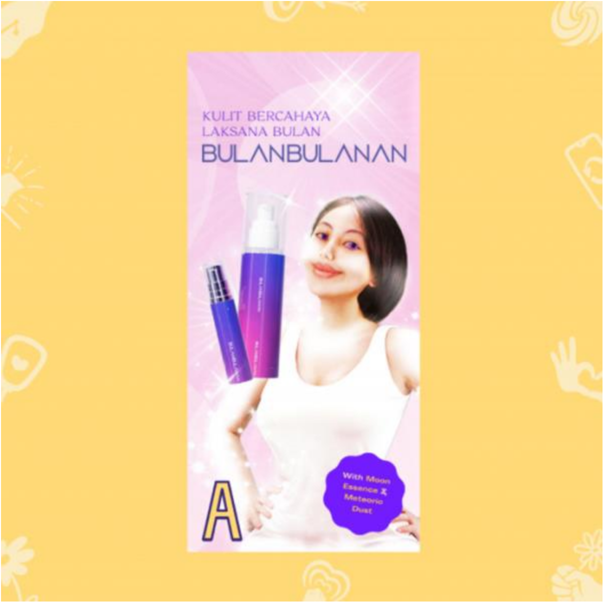
KULIT BERCAHAYA
LAKSANA BULAN
BULANBULANAN
With Moon
Essence X
Meteoric
Dust
A

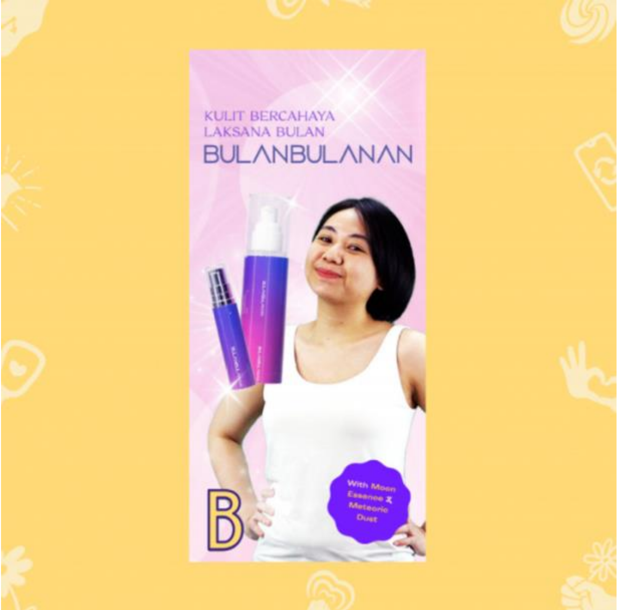

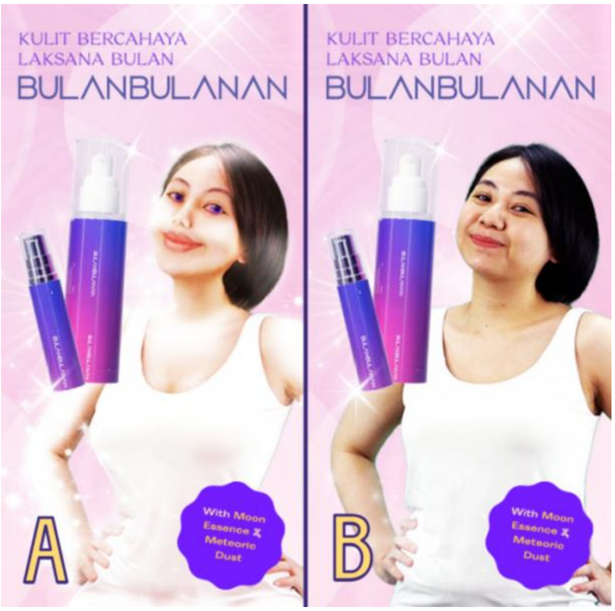

- First difference ____
- Second difference ____
- Third difference ____
- Fourth difference ____
- Fifth difference ____
- Sixth difference ____

Seventh difference ____

Eighth difference ____

----Page Break----

V2 #4BonusActivity3
Here is the fourth activity:

Omg!?! Turns out ads aren't only selling stuff! 😯 Look at this example: it's not only claiming that it can make your appearance 'perfect', it also is trying to sell you an unrealistic lifestyle! 🤯 In your opinion, what kind of lifestyle are they trying to sell? 🤔 Have you ever thought about buying something that you thought could make you cooler or fancier? 😫 What kind of product was it? What did you end up doing? 🤨

Please share your experiences!

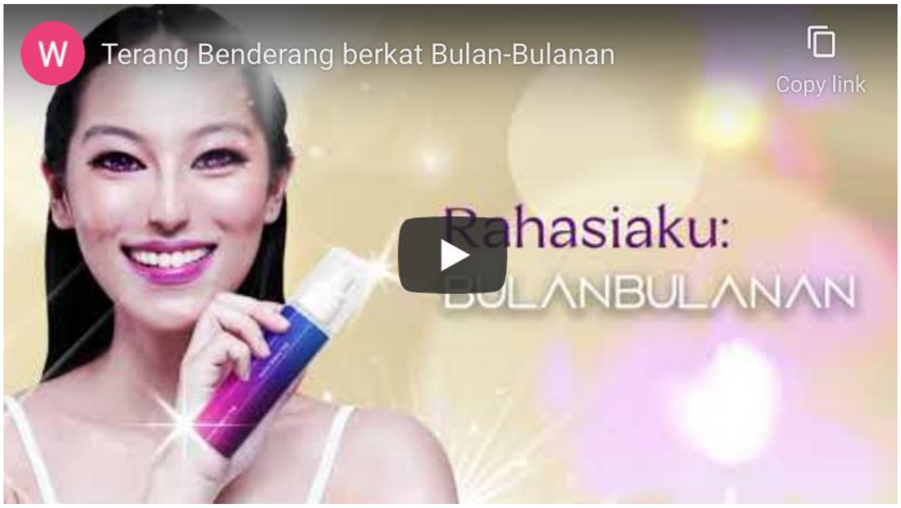

____
____
____
____
____

----Page Break----

V2 #5 YWC
Here is the final activity:

The internet has its benefits, but it also contains things that could be harmful 😫… like beauty product ads that use fake images and have unclear ingredient lists while also offering an instant solution. 🥴

Have you ever experienced or encountered such things? 😩 What kind of product was it and what did you do? When did you realise that this product was problematic? What do you know now about this product that you wish you had known before? ⏰ How would you explain the problems with this product to a friend? 🤔❓

Tell us in 250 words or less 📝✍🏽 and get a chance to win 50k phone credit! 😍👍🏽Responses from everyone participating in the research will be read, and you'll be contacted in a few weeks if you share the BEST response ✨

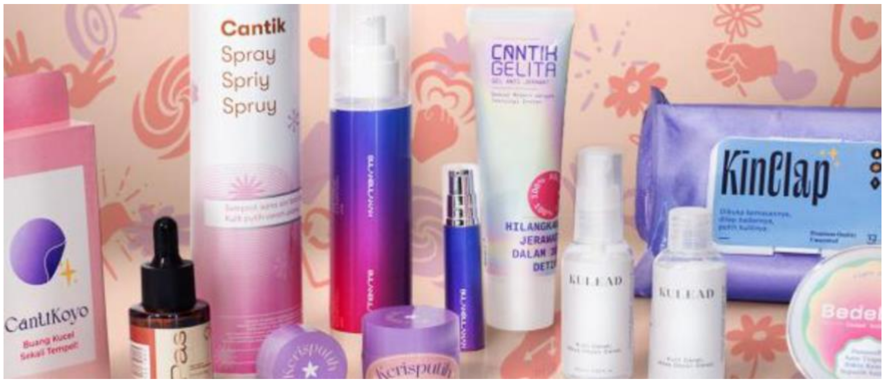

____
____
____
____
____

End of Block: Reinforcer / Page Break

Start of Block: Internet connection

Internet connection
You have completed your tasks for today – thank you!! 🌷
Before you go, can you please let us know what your internet connection was like whilst viewing the video?

○ I had a good connection and was able to view the video easily.

○ My connection was okay but I sometimes struggled to view the video.

○ I had poor connection and struggled to watch the video.

----Page Break----

Display This Question:
If You have completed your tasks for today – thank you!! 🌷 Before you go, can you please let us know... = My connection was okay but I sometimes struggled to view the video.
Or You have completed your tasks for today – thank you!! 🌷 Before you go, can you please let us know... = I had poor connection and struggled to watch the video.

UWE Bristol University of the West of England

Internet problems
Please describe the problem(s) you had.

____
____
____
____
____

End of Block: Internet connection / Page Break

Start of Block: Final msg

Thank you Thank you for your participation! 🤩

We will share the link to the third video in the Warna-Warni Waktu series tomorrow. 📅

Many thanks again for helping us with this important work! 💥

Please click "Next" to close the survey. ➡️

End of Block: Final msg

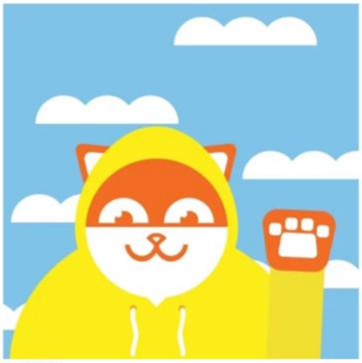

via GIPHY

Intervention and state measures questionnaire 3

Start of Block: Participant Number

Participant number
Please enter the Participant Number that the researcher gave you.

____

End of Block: Participant Number / Page Break

Start of Block: Consent

Consent
Hello again! 👋 Today we are sharing the third episode of a series of six videos for you to watch. Just like the previous two videos, today's episode is less than 5 minutes long.   Please try and watch this video in a quiet area free from distractions.   Sound good/ok?

◯ Yes, let's go!

◯ No, thanks!

Skip To: End of Block If Hello again! 👋 Today we are sharing the third episode of a series of six videos for you to wat... = Yes, let's go!

Display This Question:

If Hello again! 👋 Today we are sharing the third episode of a series of six videos for you to wat... = No, thanks!

Consent check
Are you sure? We really value your thoughts and opinions and would like very much for you to participate.

◯ I am sure. I do not want to take part today.

◯ I would like to take part in this study today.

Then Branch If:
If Are you sure? We really .... = I am sure. I do not want to take part today.
Then: End of Survey

End of Block: Consent / Page Break

Start of Block: UPC

UPC

Thank you! 🌟

Before we start, please complete the two questions below that together make up your **unique participant code**.

UPC first name

1. Write the first two letters of your first name.
(Example: if your name is ARIF, you would enter AR).

____

UPC month

2. Select the **MONTH** you were born in.

- ○ January
- ○ February
- ○ March
- ○ April
- ○ May
- ○ June
- ○ July
- ○ August
- ○ September
- ○ October
- ○ November
- ○ December

End of Block: UPC / Page Break

Start of Block: Instructions

Instructions

Our first task for you is to answer two quick questions about how you feel RIGHT NOW, IN THIS MOMENT.   Would you like a refresher on how to complete the questions?

◯ Yes, please!

◯ No, I remember what to do.

Skip To: End of Block If Our first task for you is to answer two quick questions about how you feel RIGHT NOW, IN THIS MOM... = No, I remember what to do.

Display This Question:

If Our first task for you is to answer two quick questions about how you feel RIGHT NOW, IN THIS MOM... = Yes, please!

1st example question

The example question is **"**The example question is

How satisfied do you feel about your social life RIGHT NOW?

Your answer depends on how you are feeling. For instance,

- If you are not satisfied with your social life at all, you might move keep the slider at '0'.
- If are feeling a little bit satisfied with your social life, you might move the slider to ‘25’.
- If you are feeling somewhat satisfied with your social life, you might move the slider to ’50’.
- If you are feeling satisfied with your social life but not extremely satisfied, you might move the slider to ’75’.
- If you are feeling extremely satisfied with your social life, you might move the slider to ‘100’.

Okay, now you give it a try. Use the slider below and share **how satisfied you feel about your social life RIGHT NOW**.

0 = Not at all   Extremely = 100

0 10 20 30 40 50 60 70 80 90 100

----Page Break----

1st eg Q check

Now are you ready to get started?

○ Yes, I'm ready to start!

○ No, I would like to practice with another question.

Skip To: End of Block If Now are you ready to get started? = Yes, I'm ready to start!

Display This Question:

If Now are you ready to get started? = No, I would like to practice with another question.

2nd example question

Let’s try another example question.

How angry do you feel RIGHT NOW?

- If you are not angry at all, you might move keep the slider at '0'.
- If are feeling a little bit angry, you might move the slider to ‘25’.
- If you are feeling somewhat angry, you might move the slider to ’50’.
- If you are feeling angry but not extremely angry you might move the slider to ’75’.
- If you are feeling extremely angry, you might move the slider to ‘100’.

Okay, now you give it a try.

0 = Not at all Extremely = 100

0 10 20 30 40 50 60 70 80 90 100

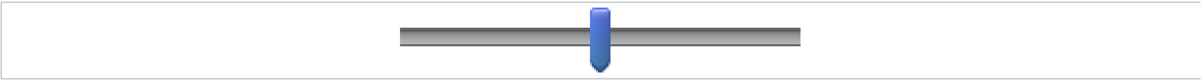

----Page Break----

2nd ex Q check

Are you ready to get started?

◯ Yes, let me get started!

◯ No, I'm not sure.

Skip To: End of Block If Are you ready to get started? = Yes, let me get started!

Display This Question:

If Are you ready to get started? = No, I'm not sure.

Extra help required

Please WhatsApp the researcher for further explanation on how to complete the questions.

After speaking with the researcher and are clear on how to complete the questions, please click "Next" to begin. ➡

End of Block: Instructions / Page Break

Start of Block: Questions

Display This Question:

If Now are you ready to get started? = Yes, I'm ready to start!

Or Are you ready to get started? = Yes, let me get started!

Or Our first task for you is to answer two quick questions about how you feel RIGHT NOW, IN THIS MOM... = No, I remember what to do.

Start Let's begin! 🚀 (And remember, the researcher will be available via WhatsApp in case you have any questions.)

----Page Break----

pre-video BI V3

How satisfied do you feel about your appearance (the way you look), **RIGHT NOW**?

0 = Not at all	Extremely = 100

0 10 20 30 40 50 60 70 80 90 100

----Page Break----

UWE Bristol | University of the West of England

pre-video mood V3

How happy do you feel, **RIGHT NOW**?

0 = Not at all   Extremely = 100

0   10   20   30   40   50   60   70   80   90 100

----Page Break----

Video 3

Now you are ready to watch episode three in the six-episode series, Warna-Warni Waktu.

Just a reminder that you might hear or see instructions that reference social media during this video.

Remember that you don't need to take any action except for watching the video 👀 and completing the exercises after the video ✍️

Please press the "Play" arrow to begin ▶️

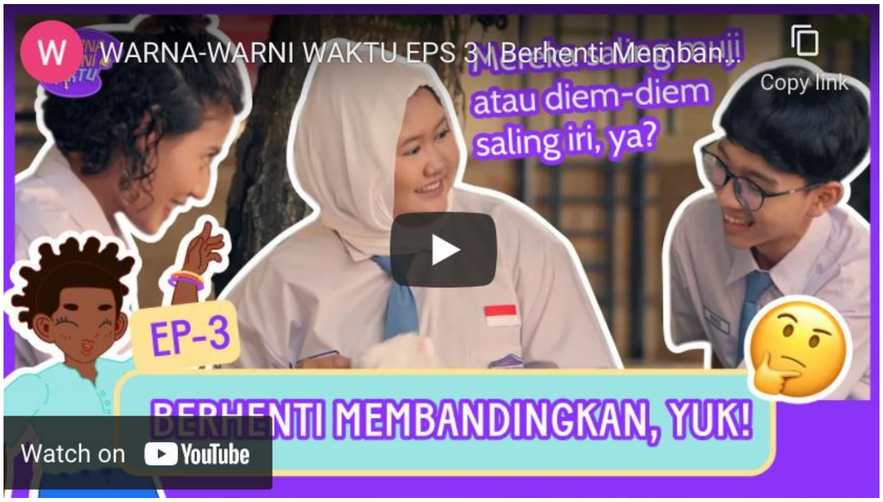

Timer 270 seconds (4:30 minutes)
Timing
First Click
Last Click
Page Submit

----Page Break----

Post-video intro V3
Now, please answer two more quick questions about how you feel **RIGHT NOW**.

----Page Break----

Post-video BI V3
How satisfied do you feel about your appearance (the way you look), **RIGHT NOW**?

0 = Not at all   Extremely = 100

0 10 20 30 40 50 60 70 80 90 100

----Page Break----

post-video mood V3
How happy do you feel, **RIGHT NOW**?

0 = Not at all   Extremely = 100

0 10 20 30 40 50 60 70 80 90 100

End of Block: Questions / Page Break

Start of Block: Reinforcer

Reinforcer intro

Our last task for you today is to complete three short activities that are related to the video you just watched.

----Page Break----

V3 #1 CTA repeat

Here is the first activity:

Friendships make our life more colourful! 👩🏽🫶👩🏾

One way we can show our appreciation for our friends is by telling them things that you like the most about them. (Remember, make sure these things are unrelated to appearance) ❤️❤️❤️ Share examples of what you would say to your closest friends below.

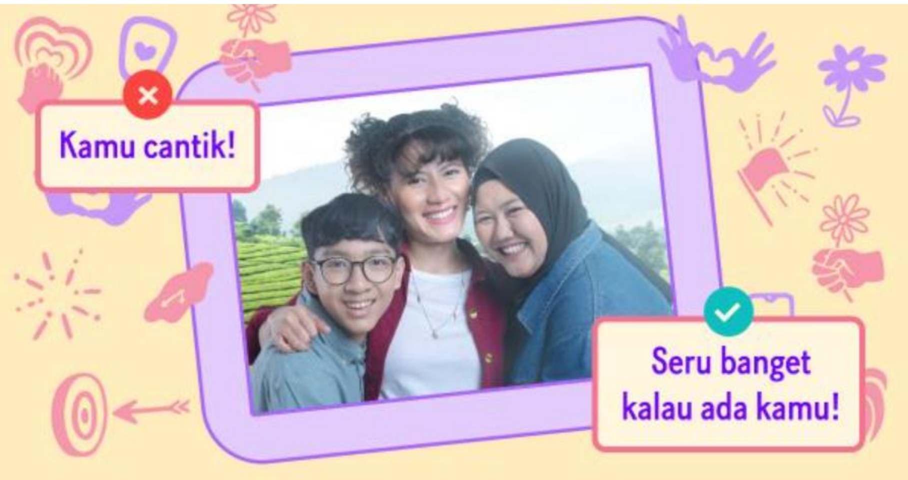

____

____

____

____

----Page Break----

V3 #1 Bonus

Here is the second activity:

Making comparisons with others can be tricky and we don't always say the right thing. 😓

This is your chance to say anything you want regarding this issue by completing the last box of this comic. 🌟🌟🌟

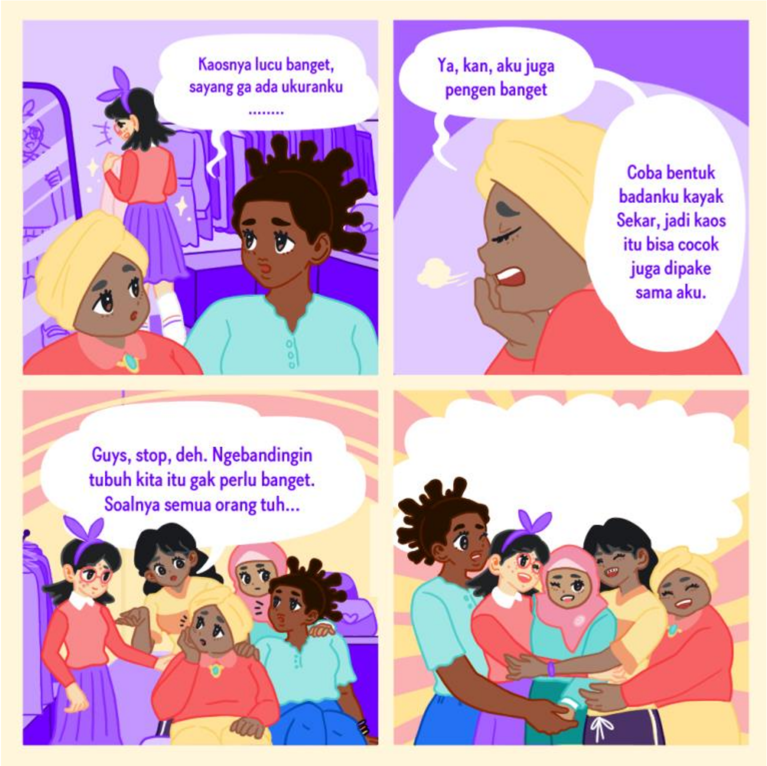

----Page Break----

V3 #3 YWC

Here is the third activity:

Have you ever looked at your friends and feel your heart is so full of pride and happiness because of them? 🥺 Me too! ✌🏿 There are so many things that I love about my friends! One of them is such a quick-thinker ⚡ that they can solve any problem. Another friend of mine is so creative 💃🏽 I am never bored when I am with them! 👩🏽🫶👩🏾

Now it's your turn! Tell us what things unrelated to appearance that you love about your friends in 250 words or less and get a chance to win 50k phone credit! 💥💥

Just like yesterday's 250-word activity, you'll be contacted in a few weeks if you give the BEST response amongst those participating in this research ✨

UWE Bristol University of the West of England

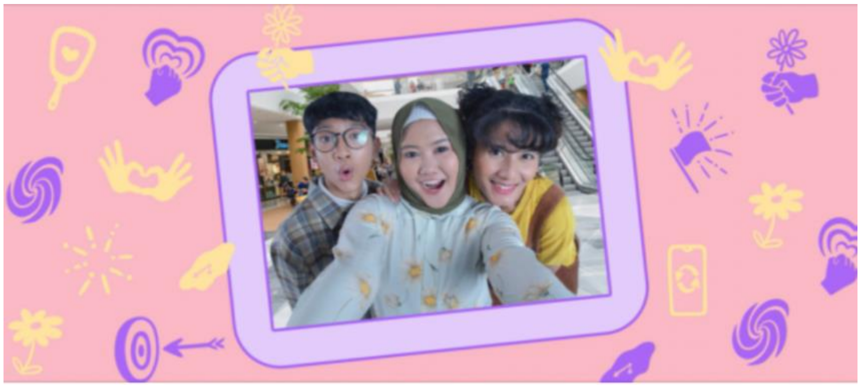

____
____
____
____
____

End of Block: Reinforcer / Page Break

Start of Block: Internet connection

Internet connection
You have completed your tasks for today – thank you!! 🙏
Before you go, can you please let us know what your internet connection was like whilst viewing the video?

◯ I had a good connection and was able to view the video easily.

◯ My connection was okay but I sometimes struggled to view the video.

◯ I had poor connection and struggled to watch the video.

Display This Question:

If You have completed your tasks for today – thank you!! 🙏 Before you go, can you please let us kno... = My connection was okay but I sometimes struggled to view the video.

Or You have completed your tasks for today – thank you!! 🙏 Before you go, can you please let us kno... = I had poor connection and struggled to watch the video.

Internet problems
Please describe the problem(s) you had.

____
____
____

End of Block: Internet connection / Page Break

Start of Block: Final msg

Thank you

Thank you for your participation! 🙌

We will share the link to the fourth video in the Warna-Warni Waktu series tomorrow. 📅

Many thanks again for helping us with this important work! 💫

Please click "Next" to close the survey. ➡

End of Block: Final msg

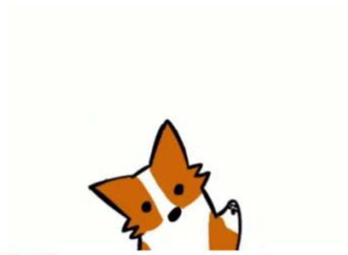

via GIPHY

UWE Bristol University of the West of England

Intervention and state measures questionnaire 4

Start of Block: Participant Number

Participant number
Please enter the Participant Number that the researcher gave you.

____

End of Block: Participant Number / Page Break

Start of Block: Consent

Consent
Wonderful to have you join us again! 🌈

Today we are sharing the fourth episode in our six-episode series.

Similar to the other videos you've watched, today's episode is less than 5 minutes long. If you can, please find a quiet spot to watch it.

Sound good?

- ○ Yes, let's go!
- ○ No, thanks!

Skip To: End of Block If Wonderful to have you join us again! 🌈 Today we are sharing the fourth episode in our six-episo... = Yes, let's go!

Display This Question:
If Wonderful to have you join us again! 🌈 Today we are sharing the fourth episode in our six-episo... = No, thanks!

Consent check
Are you sure? We really value your thoughts and opinions and would like very much for you to participate.

- ○ I am sure. I do not want to take part today.
- ○ I would like to take part in this study today.

Then Branch If:
If Are you sure? = I am sure. I do not want to take part today.
Then: End of Survey

End of Block: Consent / Page Break

Start of Block: UPC

UPC

Thank you! 🤩

Before we start, please complete the two questions below that together make up your **unique participant code**.

UPC first name

1. Write the first two letters of your first name.
(Example: if your name is ARIF, you would enter AR).

____

UPC month

2. Select the **MONTH** you were born in.

- ○ January
- ○ February
- ○ March
- ○ April
- ○ May
- ○ June
- ○ July
- ○ August
- ○ September
- ○ October
- ○ November
- ○ December

End of Block: UPC / Page Break

Start of Block: Instructions

Instructions

Our first task for you is to answer two quick questions about how you feel RIGHT NOW, IN THIS MOMENT.

Would you like a refresher on how to complete the questions in the survey?

- ○ Yes, please!
- ○ No, I remember what to do.

Skip To: End of Block If Our first task for you is to answer two quick questions about how you feel RIGHT NOW, IN THIS MOM... = No, I remember what to do.
Display This Question:
If Our first task for you is to answer two quick questions about how you feel RIGHT NOW, IN THIS MOM... = Yes, please!

1st example question
The example question is **How satisfied do you feel about your social life RIGHT NOW**?"

Your answer depends on how you are feeling. For instance,

- If you are not satisfied with your social life at all, you might move keep the slider at '0'.
- If are feeling a little bit satisfied with your social life, you might move the slider to ‘25’.
- If you are feeling somewhat satisfied with your social life, you might move the slider to ’50’.
- If you are feeling satisfied with your social life but not extremely satisfied, you might move the slider to ’75’
- If you are feeling extremely satisfied with your social life, you might move the slider to ‘100’.

Okay, now you give it a try. Use the slider below and share **how satisfied you feel about your social life RIGHT NOW**

0 = Not at all | Extremely = 100

0 10 20 30 40 50 60 70 80 90 100

----Page Break----

1st eg Q check
Do you understand how to answer this type of question?

○ Yes, I'm ready to start!

○ No, I would like to practice with another question.

Skip To: End of Block If Do you understand how to answer this type of question? = Yes, I'm ready to start!
Display This Question:
If Do you understand how to answer this type of question? = No, I would like to practice with another question.

2nd example question

Let’s try another example question.

How angry do you feel RIGHT NOW?

- If you are not angry at all, you might move keep the slider at '0'.
- If are feeling a little bit angry, you might move the slider to ‘25’.
- If you are feeling somewhat angry, you might move the slider to ’50’.
- If you are feeling angry but not extremely angry you might move the slider to ’75’.
- If you are feeling extremely angry, you might move the slider to ‘100’.

Okay, now you give it a try. How angry do you feel RIGHT NOW?

0 = Not at all Extremely = 100

0 10 20 30 40 50 60 70 80 90 100

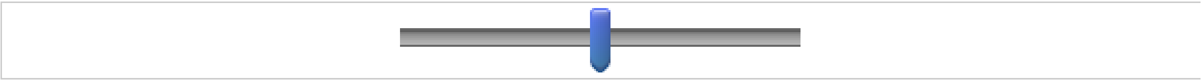

----Page Break----

2nd ex Q check

Do you now understand how to complete this type of question?

- ◯ Yes, let me get started!
- ◯ No, I'm not sure.

Skip To: End of Block If Do you now understand how to complete this type of question? = Yes, let me get started!

Display This Question:

If Do you now understand how to complete this type of question? = No, I'm not sure.

Extra help required

Please WhatsApp the researcher for further explanation on how to complete the questions.

After speaking with the researcher and are clear on how to complete the questions, please click "Next" to begin. →

End of Block: Instructions / Page Break

Start of Block: Questions

UWE Bristol University of the West of England

Display This Question:
If Do you understand how to answer this type of question? = Yes, I'm ready to start!
Or Do you now understand how to complete this type of question? = Yes, let me get started!
Or Our first task for you is to answer two quick questions about how you feel RIGHT NOW, IN THIS MOM... = No, I remember what to do.

Start

Let's begin! 🚀 (And remember, the researcher will be available via WhatsApp in case you have any questions.)

----Page Break----

pre-video BI V4

How satisfied do you feel about your appearance (the way you look), **RIGHT NOW**?

0 = Not at all Extremely = 100

0 10 20 30 40 50 60 70 80 90 100

----Page Break----

pre-video mood V4

How happy do you feel, **RIGHT NOW**?

0 = Not at all Extremely = 100

0 10 20 30 40 50 60 70 80 90 100

Episode 4 video

Now you are ready to watch episode four in the six-episode series, Warna-Warni Waktu.

Just a reminder that you might hear or see instructions that reference social media during this video.

Remember that you don't need to take any action except for watching the video 👀 and completing the exercises after the video ✍🏽

Please press the "Play" arrow to begin ▶️

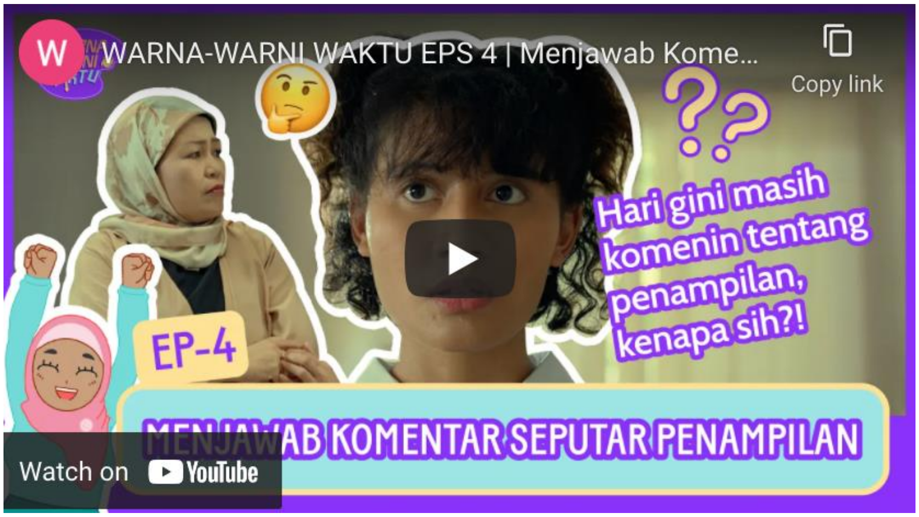

Timer 260 seconds (4:20 minutes)
Timing
First Click
Last Click
Page Submit

----Page Break----

Intro post-video
Now, please answer two more quick questions about how you feel **RIGHT NOW**.

----Page Break----

Post-video BI V4
How satisfied do you feel about your appearance (the way you look), **RIGHT NOW**?

0 = Not at all Extremely = 100

0 10 20 30 40 50 60 70 80 90 100

----Page Break----

post-video mood V4

How happy do you feel, **RIGHT NOW**?

0 = Not at all	Extremely = 100

0 10 20 30 40 50 60 70 80 90 100

End of Block: Questions / Page Break

Start of Block: Reinforcers

Reinforcer intro

Our last task for you today is to complete four short activities that are related to the video you just watched.

----Page Break----

V4 #CTA

Here is the first activity:

When there's someone close to you, like a friend 👩🏽🤝👩🏾 or a family member 👩‍👩‍👧‍👦 who says something negative about your appearance, how would you respond? 💬

Share what you would say if someone made a negative comment about your appearance! 🗣️

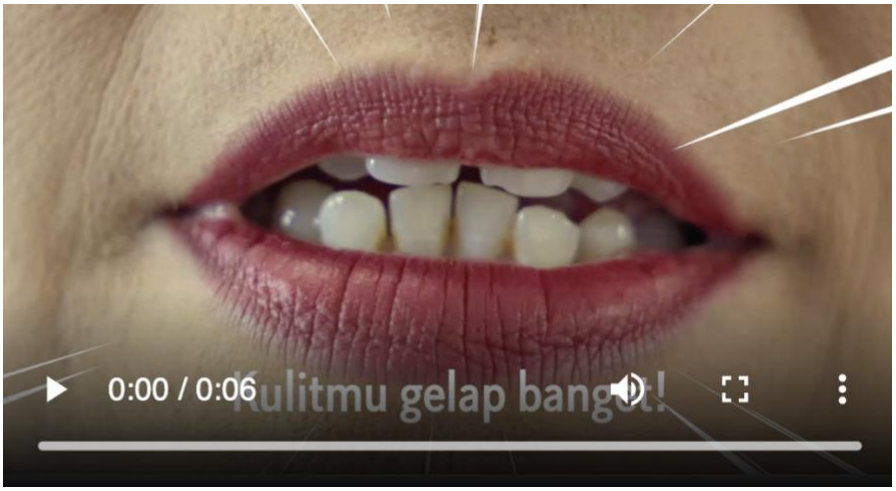

____

____

____

----Page Break----

V4 #2Bonus1

Here is the second activity:

Sometimes, we don't know what to say when someone makes a comment about our appearance! 😫

There are 10 hidden phrases in this puzzle that you can use to respond to appearance-related comments. Can you find them all? 👀🧠

Write your answers in the boxes listed below ⬇

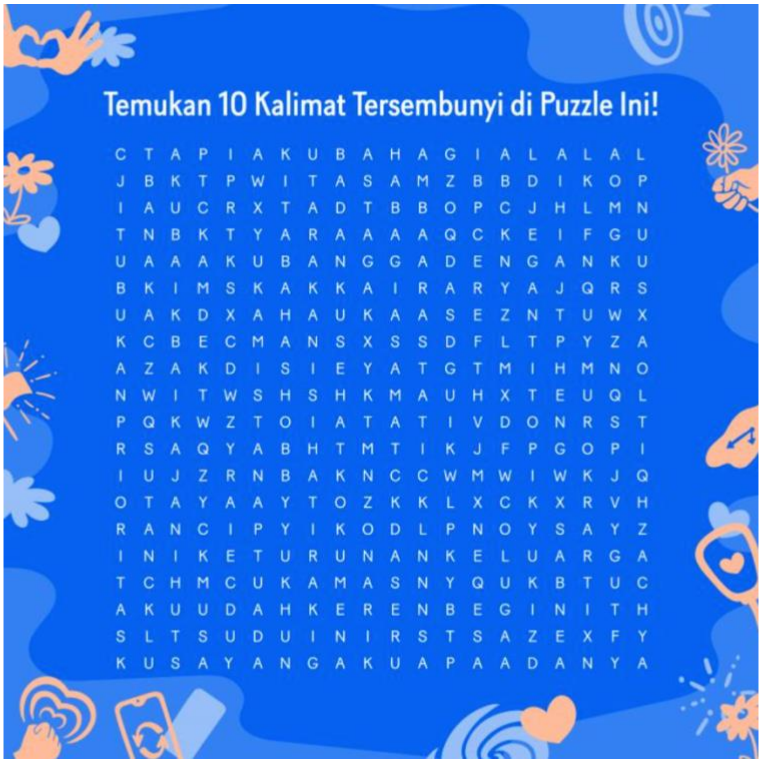

◯ First phrase ____

◯ Second phrase ____

◯ Third phrase ____

◯ Fourth phrase ____

◯ Fifth phrase ____

◯ Sixth phrase ____

◯ Seventh phrase ____

◯ Eighth phrase ____

◯ Ninth phrase ____

◯ Tenth phrase ____

----Page Break----

V4 #3Bonus2A

Here is the third activity:

What do you think Ratih, Riri, and Sekar said that made these boys bow for forgiveness? 🙇

Write down your version of their reply! ✍️

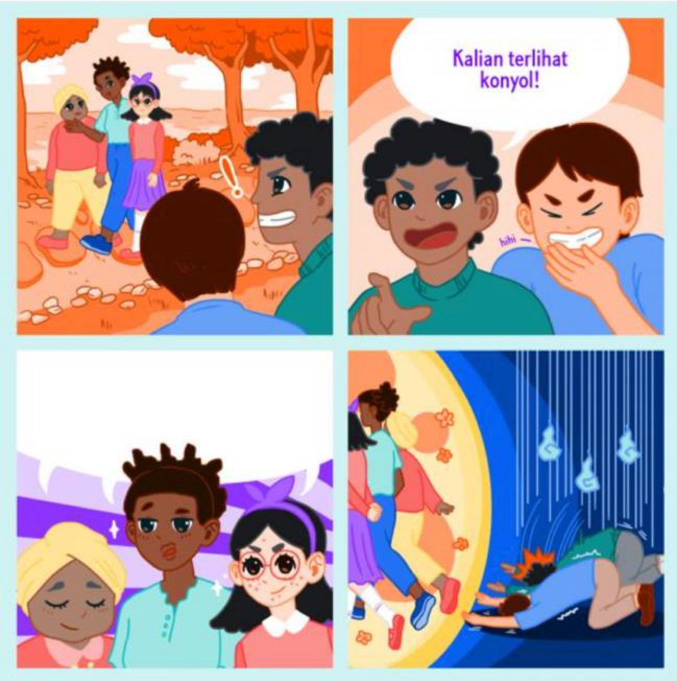

____
____
____
____
____

----Page Break----

V4 #4 YWC

Here is the fourth activity:

When there's someone close to you, like a friend or a family member who makes a negative comment about your appearance, how would you respond so as to stand up to appearance comments? 🤔

Share your answer in maximum of 250 words and get a chance to win 50k phone credit! 📝

Just like yesterday's 250-word activity, you'll be contacted in a few weeks if you give the BEST response from those participating in this research 🏆

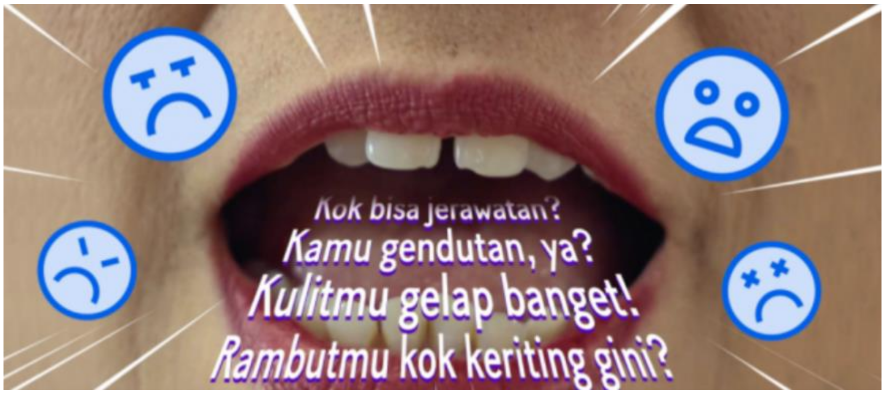

____
____
____
____
____

End of Block: Reinforcers / Page Break

Start of Block: Internet connection

Internet connection

You have completed your tasks for today – thank you!! ✏️

Before you go, can you please let us know what your internet connection was like whilst viewing the video?

- ◯ I had a good connection and was able to view the video easily.
- ◯ My connection was okay but I sometimes struggled to view the video.
- ◯ I had poor connection and struggled to watch the video.

Display This Question:
If You have completed your tasks for today – thank you!! ✏️ Before you go, can you please let us k... = My connection was okay but I sometimes struggled to view the video.
Or You have completed your tasks for today – thank you!! ✏️ Before you go, can you please let us k... = I had poor connection and struggled to watch the video.

Internet problems

Please describe the problem(s) you had.

____

____

____

____

End of Block: Internet connection / Page Break

Start of Block: Final msg

Thank you

Thank you for your participation! 🥰

We will share the link to the fifth video in the Warna-Warni Waktu series tomorrow. 📅

Many thanks again for helping us with this important work!

Please click "Next" to close the survey ➡️

End of Block: Final msg

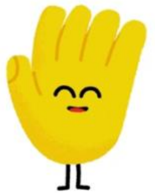

via GIPHY

Intervention and state measures questionnaire 5

Start of Block: Participant Number

Participant number
Please enter the Participant Number that the researcher gave you.

____

End of Block: Participant Number / Page Break

Start of Block: Consent

Consent
Welcome back! 👋🏾 Today we are sharing the fifth episode in our six-episode series.

Similar to the other videos you've watched, today's episode is less than 5 minutes long. If you can, please find a quiet spot to watch it.

Sound good?

○ Yes, let's go!

○ No, thanks!

Skip To: End of Block If Welcome back! 👋 Today we are sharing the fifth episode in our six-episode series. Similar to... = Yes, let's go!

Display This Question:
If Welcome back! 👋 Today we are sharing the fifth episode in our six-episode series. Similar to... = No, thanks!

Consent check
Are you sure? We really value your thoughts and opinions and would like very much for you to participate.

○ I am sure. I do not want to take part today.

○ I would like to take part in this study today.

Then Branch If:
If Are you sure? = I am sure. I do not want to take part today.
Then: End of Survey

End of Block: Consent / Page Break

Start of Block: UPC

UPC

Thank you! 🌻

Before we start, please complete the two questions below that together make up your **unique participant code**.

UPC first name

1. Write the first two letters of your first name.
(Example: if your name is ARIF, you would enter AR).

____

UPC month

2. Select the **MONTH** you were born in.

- ○ January
- ○ February
- ○ March
- ○ April
- ○ May
- ○ June
- ○ July
- ○ August
- ○ September
- ○ October
- ○ November
- ○ December

End of Block: UPC / Page Break

Start of Block: Instructions

Instructions

Our first task for you is to answer two quick questions about how you feel RIGHT NOW, IN THIS MOMENT.

Would you like a refresher on how to complete the questions in the survey?

◯ Yes, please!

◯ No, I remember what to do.

Skip To: End of Block If Our first task for you is to answer two quick questions about how you feel RIGHT NOW, IN THIS MOM... = No, I remember what to do.

Display This Question:

If Our first task for you is to answer two quick questions about how you feel RIGHT NOW, IN THIS MOM... = Yes, please!

1st example question

The example question is:

How excited do you feel RIGHT NOW?

Your answer depends on how you are feeling. For instance,

- If you are not excited at all, you might move keep the slider at '0'.
- If are feeling a little bit excited, you might move the slider to ‘25’.
- If you are feeling somewhat excited, you might move the slider to ’50’.
- If you are feeling excited but not extremely excited, you might move the slider to ’75’.
- If you are feeling extremely excited, you might move the slider to ‘100’.

Okay, now you give it a try. Use the slider below and share **how excited you feel RIGHT NOW**.

0 = Not at all   Extremely = 100

0 10 20 30 40 50 60 70 80 90 100

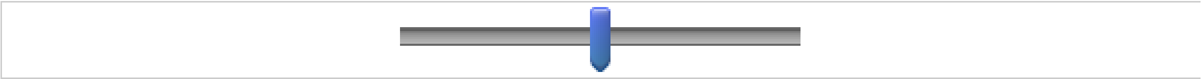

----Page Break----

1st eg Q check

Do you understand how to answer this type of question?

◯ Yes, I'm ready to start!

◯ No, I would like to practice with another question.

Skip To: End of Block If Do you understand how to answer this type of question? = Yes, I'm ready to start!

Display This Question:

If Do you understand how to answer this type of question? = No, I would like to practice with another question.

2nd example question

Let’s try another example question.

How tired do you feel RIGHT NOW?

- If you are not tired at all, you might move keep the slider at '0'.
- If are feeling a little bit tired, you might move the slider to ‘25’.
- If you are feeling somewhat tired, you might move the slider to ’50’.
- If you are feeling tired but not extremely tired you might move the slider to ’75’.
- If you are feeling extremely tired, you might move the slider to ‘100’.

Okay, now you give it a try.

0 = Not at all   Extremely = 100

0 10 20 30 40 50 60 70 80 90 100

----Page Break----

2nd ex Q check

Do you now understand how to complete this type of question?

◯ Yes, let me get started!

◯ No, I'm not sure.

Skip To: End of Block If Do you now understand how to complete this type of question? = Yes, let me get started!
Display This Question:
If Do you now understand how to complete this type of question? = No, I'm not sure.

Extra help required
Please WhatsApp the researcher for further explanation on how to complete the questions.

After speaking with the researcher and are clear on how to complete the questions, please click "Next" to begin. ➡

End of Block: Instructions / Page Break

Start of Block: Questions
Display This Question:
If Do you understand how to answer this type of question? = Yes, I'm ready to start!
Or Do you now understand how to complete this type of question? = Yes, let me get started!
Or Our first task for you is to answer two quick questions about how you feel RIGHT NOW, IN THIS MOM... = No, I remember what to do.

Start
Let's begin! 🚀 (And remember, the researcher will be available via WhatsApp in case you have any questions.)

----Page Break----

pre-video BI V5
How satisfied do you feel about your appearance (the way you look), **RIGHT NOW**?

0 = Not at all Extremely = 100

0 10 20 30 40 50 60 70 80 90 100

----Page Break----

pre-video mood V5
How happy do you feel, **RIGHT NOW**?

0 = Not at all Extremely = 100

0 10 20 30 40 50 60 70 80 90 100

----Page Break----

Episode 5 video

Now you are ready to watch episode five in the six-episode series, Warna-Warni Waktu.

Just a reminder that you might hear or see instructions that reference social media during this video.

Remember that you don’t need to take any action except for watching the video 👀 and completing the exercises after the video ✍🏽

Please press the "Play" arrow to begin ➡️

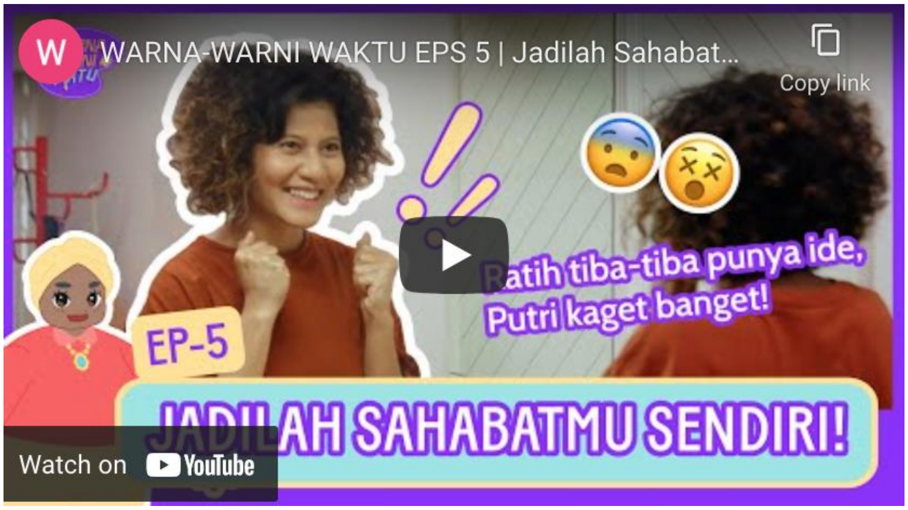

Timer 330 seconds (5:30 minutes)
Timing
First Click
Last Click
Page Submit
Click Count

----Page Break----

Intro post-video

Now, please answer two more quick questions about how you feel **RIGHT NOW**.

----Page Break----

Post-video BI V5
How satisfied do you feel about your appearance (the way you look), **RIGHT NOW**?

0 = Not at all Extremely = 100

0 10 20 30 40 50 60 70 80 90 100

----Page Break----

post-video mood V5
How happy do you feel, **RIGHT NOW**?

0 = Not at all Extremely = 100

0 10 20 30 40 50 60 70 80 90 100

End of Block: Questions / Page Break

Start of Block: Reinforcer

Reinforcer intro
Our last task for you today is to complete three short activities that are related to the video you just watched.

----Page Break----

V5 #1 CTA
Here is the first activity:

Do you have a mantra? ✨ A mantra is something that you can say to yourself to boost your confidence. 🥰

Laras' mantra is, "I'm resourceful and clever." How about you? What’s your mantra?

If you don’t have one yet, create one and write it down here! 😍

You can even stick your mantra in a place where you’ll see it every day, like the mantra above Putri’s desk! 💪🏽

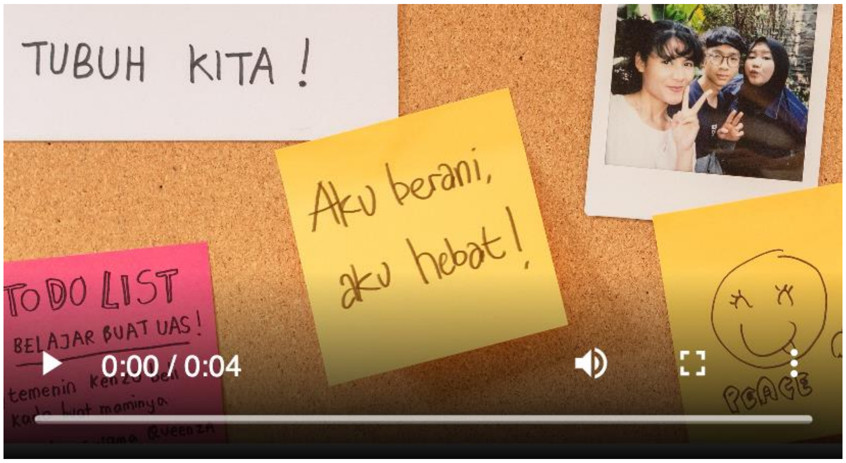

----Page Break----

V5 #2 Bonus

Here is the second activity:

Our bodies are so incredible and do so many things for us, 😍 like helping us to smell the garlic frying down the street 😋, falling over with laughter when your friends tell a great joke, or wrapping our arms around the people we love. 🥰 Putri's body helped her to succeed at sports!

What has your body helped you to do? Write your answer here!

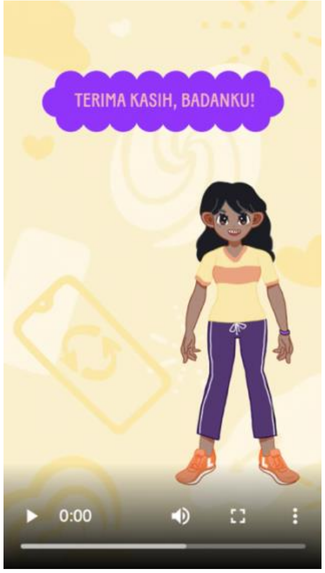

---Page Break----

V5 #3 YWC

Here is the third activity:

Hi, friends! ✨ Have you had a bad day where you see nothing but negative things about yourself? You're not alone. When Putri had a bad day, she created a mantra that said, "I can do it!" 💪🏿 and repeated it to herself to give herself the energy and confidence she needed to keep going.

Do you have a mantra? Share your mantra with us! What does it mean to you and how did you come up with it?

The most inspiring mantra will get a chance to win 50K of mobile phone credit! 😍 Just like yesterday's 250-word activity, you'll be contacted in a few weeks if you give the BEST response amongst those participating in this research! ✨

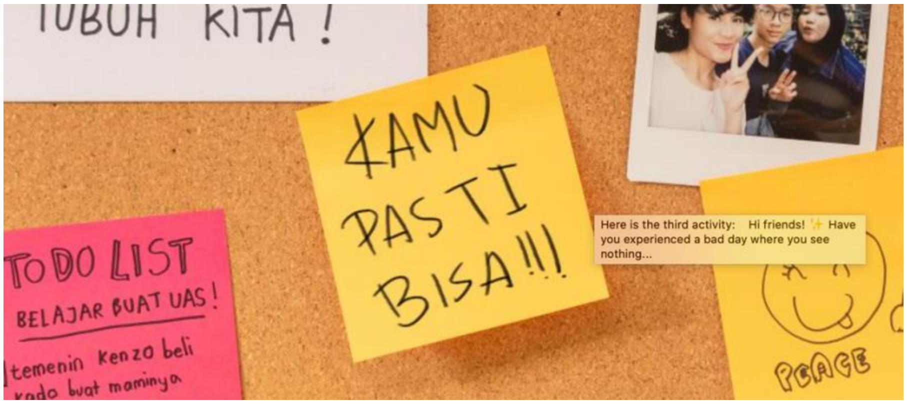

End of Block: Reinforcer / Page Break

Start of Block: Internet connection

Internet connection

You have completed your tasks for today – thank you!! 🌈

Before you go, can you please let us know what your internet connection was like whilst viewing the video?

- ◯ I had a good connection and was able to view the video easily.
- ◯ My connection was okay but I sometimes struggled to view the video.
- ◯ I had poor connection and struggled to watch the video.

Display This Question:

If You have completed your tasks for today – thank you!! 🌈 Before you go, can you please let us kn... = My connection was okay but I sometimes struggled to view the video.

Or You have completed your tasks for today – thank you!! 🌈 Before you go, can you please let us kn... = I had poor connection and struggled to watch the video.

Internet problems

Please describe the problem(s) you had.

____

____

____

____

End of Block: Internet connection / Page Break

Start of Block: Final msg

Thank you

Thank you for your participation! 😍

We will share the link to the sixth and final video in the Warna-Warni Waktu series tomorrow. 📅

Many thanks again for helping us with this important research! ✨

Please click "Next" to close the survey. ➡️

End of Block: Final msg

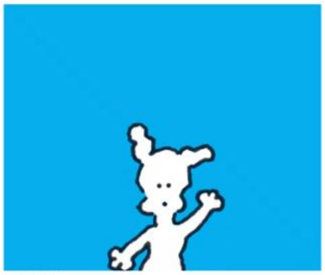

via GIPHY

Intervention and state measures questionnaire 6

Start of Block: Participant Number

Participant number
Please enter the Participant Number that the researcher gave you.

____

End of Block: Participant Number / Page Number

Start of Block: Consent

Consent
Great to have you back! 👋

Today we are sharing the sixth and final episode in our six-episode series.

Like yesterday's video, today's episode is less than 5 minutes long. Please try and watch this video in a quiet area free from distractions.

Sound good?

○ Yes, let's go!

○ No, thanks!

Skip To: End of Block If Great to have you back! 👋 Today we are sharing the sixth and final episode in our six-episode... = Yes, let's go!

Display This Question:
If Great to have you back! 👋 Today we are sharing the sixth and final episode in our six-episode... = No, thanks!

Consent check
Are you sure? We really value your thoughts and opinions and would like very much for you to participate.

○ I am sure. I do not want to take part today.

○ I would like to take part in this study today.

Then Branch If:
If Are you sure? We really .... = I am sure. I do not want to take part today.
Then: End of Survey

End of Block: Consent / Page Break

Start of Block: UPC

UPC

Thank you! 🌸

Before we start, please complete the two questions below that together make up your **unique participant code**.

UPC first name

1. Write the first two letters of your first name.
(Example: if your name is ARIF, you would enter AR).

____

UPC month

2. Select the **MONTH** you were born in.

- ○ January
- ○ February
- ○ March
- ○ April
- ○ May
- ○ June
- ○ July
- ○ August
- ○ September
- ○ October
- ○ November
- ○ December

End of Block: UPC / Page Break

Start of Block: Instructions

Instructions

Our first task for you is to answer two quick questions about how you feel RIGHT NOW, IN THIS MOMENT.   Would you like a refresher on how to complete the questions?

- ○ Yes, please!
- ○ No, I remember what to do.

Skip To: End of Block If Our first task for you is to answer two quick questions about how you feel RIGHT NOW, IN THIS MOM... = No, I remember what to do.

Display This Question:

If Our first task for you is to answer two quick questions about how you feel RIGHT NOW, IN THIS MOM... = Yes, please!

1st example question

The example question is

How thirsty do you feel RIGHT NOW?

Your answer depends on how you are feeling. For instance,

- If you are not feeling thirsty at all, you might keep the slider at '0'.
- If you are feeling a little thirsty, you might move the slider to '25'.
- If you are feeling somewhat thirsty, you might move the slider to the number '50'.
- If you are feeling thirsty but not extremely thirsty, you might move to the slider to '75'.
- If you are feeling extremely thirsty, you might move the slider to '100'.

Okay, now you give it a try. Use the slider below and share **how thirsty you feel RIGHT NOW**.

0 = Not at all Extremely = 100

0 10 20 30 40 50 60 70 80 90 100

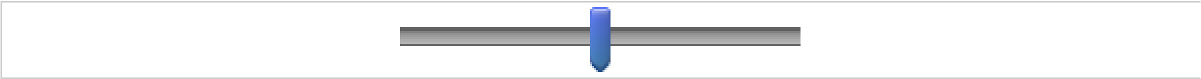

----Page Break----

1st eg Q check

Now are you ready to get started?

○ Yes, I'm ready to start!

○ No, I would like to practice with another question.

Skip To: End of Block If Now are you ready to get started? = Yes, I'm ready to start!

Display This Question:
If Now are you ready to get started? = No, I would like to practice with another question.

2nd example question
The example question is "How satisfied do you feel about your social life **RIGHT NOW**?

Your answer depends on how you are feeling. For instance,

- If you are not satisfied with your social life at all, you might move keep the slider at '0'.
- If are feeling a little bit satisfied with your social life, you might move the slider to ‘25’.
- If you are feeling somewhat satisfied with your social life, you might move the slider to ’50’.
- If you are feeling satisfied with your social life but not extremely satisfied, you might move the slider to ’75’.
- If you are feeling extremely satisfied with your social life, you might move the slider to ‘100’.

Okay, now you give it a try. Use the slider below and share how satisfied you feel about your social life RIGHT NOW.

0 = Not at all Extremely = 100

0 10 20 30 40 50 60 70 80 90 100

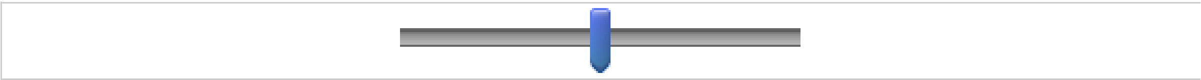

----Page Break----

2nd ex Q check
Are you ready to get started?

◯ Yes, let me get started!

◯ No, I'm not sure.

Skip To: End of Block If Are you ready to get started? = Yes, let me get started!
Display This Question:
If Are you ready to get started? = No, I'm not sure.

Extra help required

Please WhatsApp the researcher for further explanation on how to complete the questions.

After speaking with the researcher and are clear on how to complete the questions, please click "Next" to begin. ➡️

End of Block: Instructions / Page Break

Start of Block: Questions

Display This Question:
If Now are you ready to get started? = Yes, I'm ready to start!
Or Are you ready to get started? = Yes, let me get started!
Or Our first task for you is to answer two quick questions about how you feel RIGHT NOW, IN THIS MOM... = No, I remember what to do.

Start

Let's begin! 🚀 (And remember, the researcher will be available via WhatsApp in case you have any questions.)

----Page Break----

pre-video BI V6

How satisfied do you feel about your appearance (the way you look), **RIGHT NOW**?

0 = Not at all Extremely = 100

0 10 20 30 40 50 60 70 80 90 100

----Page Break----

pre-video mood V6

How happy do you feel**, RIGHT NOW**?

0 = Not at all Extremely = 100

0 10 20 30 40 50 60 70 80 90 100

----Page Break----

Episode 6 video

Now you are ready to watch the last episode in the six-episode series, Warna-Warni Waktu.

Just a reminder that you might hear or see instructions that reference social media during this video.

Remember that you don't need to take any action except for watching the video 👀 and completing the exercises after the video ✍🏽

Please press the "Play" arrow to begin ➡️

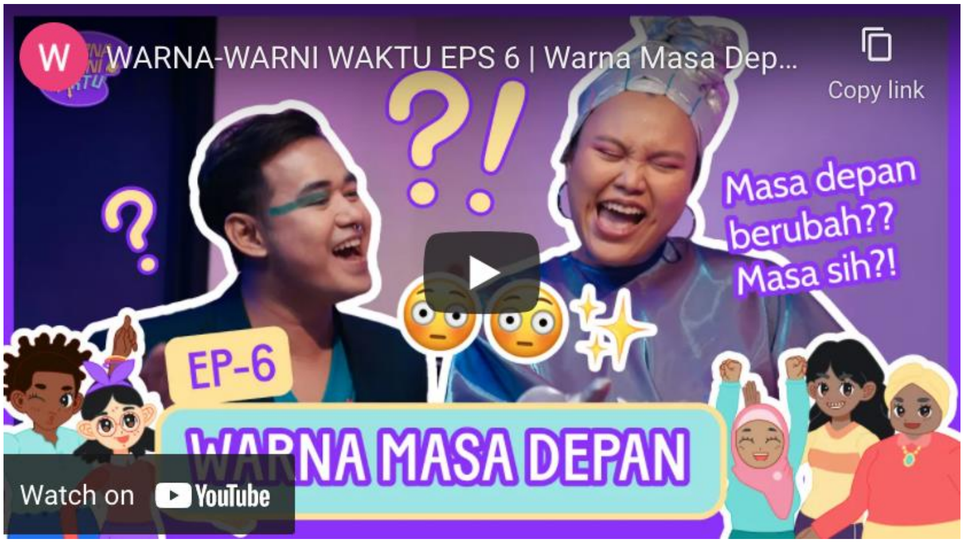

Timer 205 seconds (3:25 minutes)
Timing
First Click
Last Click
Page Submit

----Page Break----

Intro post-video Qs

Now, please answer two more quick questions about how you feel **RIGHT NOW**.

----Page Break----

Post-video BI v6
How satisfied do you feel about your appearance (the way you look), **RIGHT NOW**?

0 = Not at all Extremely = 100

0 10 20 30 40 50 60 70 80 90 100

----Page Break----

post-video mood v6
How happy do you feel, **RIGHT NOW**?

0 = Not at all Extremely = 100

0 10 20 30 40 50 60 70 80 90 100

End of Block: Questions / Page Break

Start of Block: Reinforcer

Reinforcer intro
Our last task for you today is a short activity that is related to the video you just watched.

----Page Break----

V6 #1 Bonus
In this short video, Putri shares the top four lessons she learned from those key moments in her life. 💜📝

Which of these lessons is most important to you and why? Do you have any other tips to add? 🤔 Write them down here! 🙌🏾✨

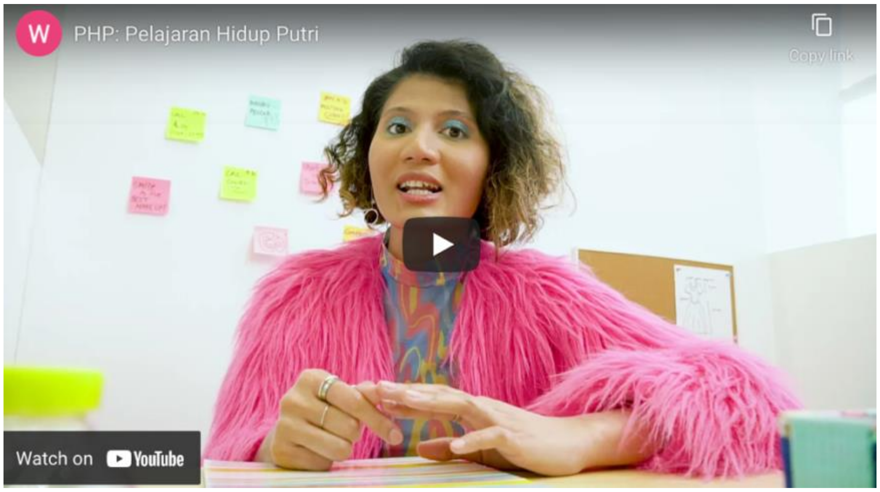

----Page Break----

V6 #2CTA

Putri has changed her world 💫 and you can change yours, too! 🙌

As a final task, we encourage you to share an unedited and unfiltered photo of yourself on social media, 🤳 a photo of you doing something that you love, like spending time with your family 👩‍👧‍👦 or hanging out with your friends! 👩🤝👩

Remember, there's no need to edit the photo or use any filters ✨... it's the REAL you that's special!

Do you commit to sharing an unedited photo of yourself on social media?

- ◯ YES - count me in 🙋
- ◯ No, it's great idea but I'm out✌

End of Block: Reinforcer / Page Break

Start of Block: Internet connection

Internet connection

You have completed your tasks for today – thank you!! 🙏

Before you go, can you please let us know what your internet connection was like whilst viewing the video?

- ◯ I had a good connection and was able to view the video easily.
- ◯ My connection was okay but I sometimes struggled to view the video.
- ◯ I had poor connection and struggled to watch the video.

----Page Break----

Display This Question:
If You have completed your tasks for today – thank you!! 🙏 Before you go, can you please let us k... = My connection was okay but I sometimes struggled to view the video.
Or You have completed your tasks for today – thank you!! 🙏 Before you go, can you please let us k... = I had poor connection and struggled to watch the video.

Internet problems

Please describe the problem(s) you had.

____
____
____
____

End of Block: Internet connection / Page Break

Start of Block: Final msg

Thank you

Many thanks for watching the Warna-Warni Waktu series and helping us with this important research! 💥

Please click "next" to close the survey ➡️

End of Block: Final msg

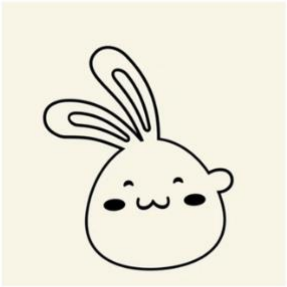

via GIPHY
